# Improvement of obesity-associated disorders by a small-molecule drug targeting mitochondria of adipose tissue macrophages

**DOI:** 10.1038/s41467-020-20315-9

**Published:** 2021-01-04

**Authors:** Yawei Wang, Binlin Tang, Lei Long, Peng Luo, Wei Xiang, Xueru Li, Huilan Wang, Qingzhi Jiang, Xu Tan, Shenglin Luo, Huijuan Li, Ziwen Wang, Zelin Chen, Yu Leng, Zhongyong Jiang, Yang Wang, Le Ma, Rui Wang, Chunyu Zeng, Zujuan Liu, Yu Wang, Hongming Miao, Chunmeng Shi

**Affiliations:** 1 Institute of Rocket Force Medicine, State Key Laboratory of Trauma, Burns and Combined Injury, Third Military Medical University, Chongqing, 400038 China; 2 Oncology Department, The General Hospital of Western Theater Command, Chengdu, Sichuan 610083 China; 3 Department of Biochemistry and Molecular Biology, Third Military Medical University, Chongqing, 400038 China; 4 Department of Clinical Medicine, Southwest Medical University, Luzhou, Sichuan 646000 China; 5 Department of Cardiology, Daping Hospital, Third Military Medical University, Chongqing, 400042 China

**Keywords:** Cell signalling, Drug delivery, Endocrine system and metabolic diseases, Biomaterials

## Abstract

Pro-inflammatory activation of adipose tissue macrophages (ATMs) is causally linked to obesity and obesity-associated disorders. A number of studies have demonstrated the crucial role of mitochondrial metabolism in macrophage activation. However, there is a lack of pharmaceutical agents to target the mitochondrial metabolism of ATMs for the treatment of obesity-related diseases. Here, we characterize a near-infrared fluorophore (IR-61) that preferentially accumulates in the mitochondria of ATMs and has a therapeutic effect on diet-induced obesity as well as obesity-associated insulin resistance and fatty liver. IR-61 inhibits the classical activation of ATMs by increasing mitochondrial complex levels and oxidative phosphorylation via the ROS/Akt/Acly pathway. Taken together, our findings indicate that specific enhancement of ATMs oxidative phosphorylation improves chronic inflammation and obesity-related disorders. IR-61 might be an anti-inflammatory agent useful for the treatment of obesity-related diseases by targeting the mitochondria of ATMs.

## Abstract (web summary)

Adipose tissue macrophages are central to controlling inflammation in the context of obesity. Here the authors present a new infrared dye (IR-61) that accumulates in the mitochondria of these cells resulting in anti-inflammatory effects that counter obesity-associated pathology in mice.

## Introduction

Obesity and obesity-associated metabolic syndromes represent a global health problem^1^. A growing number of findings have shown that chronic inflammation is an important feature of obesity and that persistent inflammation can also lead to obesity and obesity-related metabolic diseases^2,3^. Anti-inflammatory agents are considered as a treatment strategy for obesity-related disorders and have been tested in animal and human models for more than a decade^4–6^. However, this therapy has not shown significant progress in either model system^7^. Hypermedication and off-target effects have resulted in serious side effects, including Cushing’s-like syndromes, gastrointestinal bleeding, and immune suppression^8,9^. Moreover, a complicated biological network between inflammation and obesity often causes unpredictable outcomes and treatment failure^7,8,10^. The lack of more selective and effective anti-inflammatory agents for obesity treatment highlights the need to re-evaluate disease physiopathology in obesity to identify more effective therapeutic targets.

Local inflammation of adipose tissues can regulate obesity and systemic inflammation, and macrophage activation plays a decisive role in adipose tissue inflammation^11–13^. Macrophages account for 10–15% of the cell population in normal adipose tissue and mainly present with an anti-inflammatory phenotype (M2-like). However, in the state of obesity, macrophages constitute approximately 40–50% of adipose tissue cells and display a pro-inflammatory phenotype (M1-like)^14^. These pro-inflammatory adipose tissue macrophages (ATMs) secrete a range of pro-inflammatory cytokines, causing both local and systemic inflammation in an autocrine or paracrine manner^15,16^. Therefore, ATMs are considered important targets for the treatment of chronic inflammation and obesity-related metabolic diseases^17,18^. Thus far, there is a lack of pharmaceutical agents to target ATMs for obesity treatment.

Recent studies have suggested that mitochondrial metabolism plays an important role in maintaining and changing the inflammatory phenotype of macrophages^19–21^. M1 macrophages undergo metabolic changes that drive them toward glycolysis and exhibit minimal reliance on mitochondrial oxidative phosphorylation. In contrast, M2 macrophages mainly depend on oxidative phosphorylation and have increased oxygen consumption^22^. Previous research has shown that reduced oxidative phosphorylation of macrophages causes exacerbated inflammation and insulin resistance^23^. Therefore, targeting oxidative phosphorylation in macrophages hold promise for therapeutic treatment of obesity-related disorders. However, due to the lack of methodology or agents capable of simultaneously targeting ATMs and their oxidative phosphorylation, treatment is still challenged by potential off-target effects^24^. Furthermore, it is not clear if potentiating the oxidative function of ATMs would improve obesity-related disorders.

To obtain therapeutic agents targeting ATMs, current strategies mainly explore chemical drugs conjugated to various functional nanocarriers that are recognized by macrophage receptors^17,25^. However, use of these targeting nanocarriers is usually limited by high cost, instability, toxic effects, and so on^26,27^. Therefore, it is of great significance to design and develop structure-inherent small molecules targeting the mitochondria of ATMs for obesity treatment. A class of lipophilic cationic NIR fluorescence heptamethine cyanine dyes has been identified with the structure-inherent characteristic of mitochondria targeting^28–30^.

Here, we screen the dyes and characterize a small-molecule infrared dye termed IR-61, which preferentially accumulates in the mitochondria of ATMs via intraperitoneal administration, potentiates mitochondrial oxidative phosphorylation, and further improves obesity and its associated metabolic syndromes such that it represents a possible treatment strategy for obesity-related metabolic diseases.

## Results

### IR-61 targets ATMs and locates to macrophage mitochondria

**Fig. 1:**
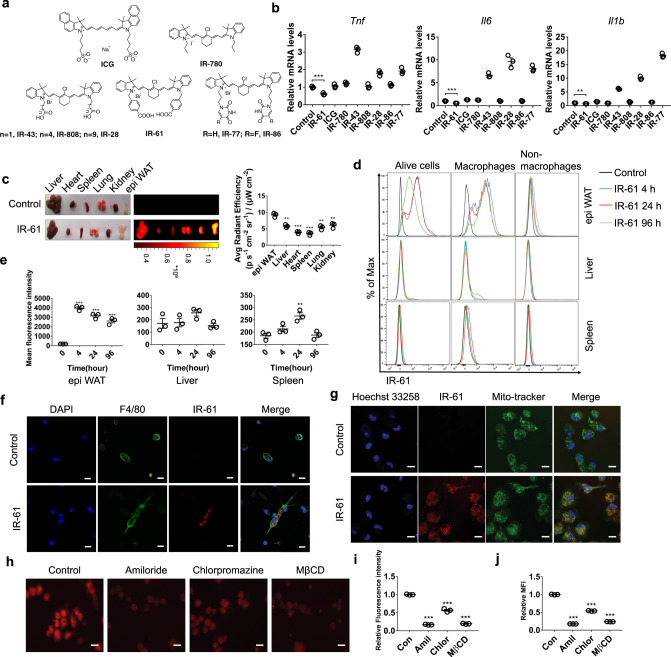
IR-61 targets ATMs and locates to macrophage mitochondria. **a** Chemical structures of ICG, IR-780, IR-43, IR-808, IR-28, IR-77, IR-86, and IR-61. **b** BMDMs were treated with different small molecules for 24 h and the gene expression of pro-inflammatory molecules was determined by qPCR. **c** Mice organs were imaged using an NIR imaging system at 24 h after intraperitoneally administered IR-61 at 2 mg kg^−1^. Average radiant efficiency was shown. **d** IR-61 level stain in total alive cells, macrophages and nonmacrophage immune cells isolated from epi WAT, liver, and spleen was determined by flow cytometry. **e** In vivo uptake of IR-61 by the macrophages was measured by mean fluorescence intensity using flow cytometry. **f** Confocal microscopy shows F4/80 (green) and IR-61 (red) in isolated SVFs from epi WAT of mice 24 h after being intraperitoneally injected with IR-61. Nuclei were stained with DAPI (blue). Representative images are displayed (scale bars, 10 μm). **g** Colocalization of IR-61 with Mito-Tracker Green in PMs imaged with a confocal microscope. Nuclei were stained with Hoechst 33258 (scale bars, 10 μm). **h** PMs were pretreated with 50 μM amiloride, 12.5 μM chlorpromazine or 7.5 mM MβCD for 1 h and then incubated with 10 μM IR-61 for 30 min prior to observation with the NIR fluorescence microscope (scale bars, 10 μm). **i** Fluorescence intensities of the samples are presented as in (**h**). **j** Mean fluorescence intensities (MFI) of PMs with the same treatment method for sample shown in (**h**) were determined by flow cytometry. Data are representative of three independent experiments. Results are presented as the mean ± SEM (*p < 0.05, **p < 0.01, ***p < 0.001, *n* = 3; two-sided Student’s *t*-test). Exact *p*-values are given in the Source Data file. Source data are provided as a Source Data file.

Our group has established a library that features newly synthesized mitochondria-targeting NIR fluorescent heptamethine cyanine dyes. Among the different group of NIR fluorescent dyes, we selected eight representative small molecules to explore their effects on macrophage inflammatory phenotype (Fig. 1a). The synthesis method of these small molecules referred to the articles previously reported^29,31^ and were also described in Supplementary Fig. 1. Of these, IR-61 was identified as a small molecule that suppressed expression of pro-inflammatory genes in bone marrow-derived macrophages (BMDMs) (Fig. 1b). IR-61 (Ex 782 nm and Em 805 nm) is an NIR fluorophore with lipophilicity and positive charge, which can self-assemble in an aqueous solution and form spherical nanoparticles with a diameter of 90 ± 5 nm. DLS measurement results indicated that the hydrodynamic diameter of IR-61 was approximately 119 nm (Supplementary Fig. 2a, b). In the present study, IR-61 was injected into age-matched C57BL/6J mice via intraperitoneal administration, and the IR-61 distribution in tissues was measured using a Kodak In-Vivo FX Professional Imaging System (Fig. 1c). We demonstrated that IR-61 predominantly accumulated in the visceral fat after intraperitoneal injection (Fig. 1c). Next, to determine the profile of cells in the epididymal fat (epi WAT), liver, and spleen that internalize IR-61, we performed flow cytometry analysis at 4, 24, and 96 h after intraperitoneal injection. We found that IR-61 was highly internalized by CD45^+^F4/80^+^ macrophages at any time point, but rarely by other immune cells in epi WAT (Fig. 1d, e). We also detected the distribution of IR-61 in liver and spleen that contain abundant macrophages in sinusoid. Results showed that the macrophages in liver and spleen exhibited only a negligible uptake of IR-61 at 24 h but IR-61 signal cannot be detected at 4 and 96 h, which, due to subtle IR-61, was internalized by macrophages nonspecifically and then quickly cleared during the metabolic process (Fig. 1d, e). Using fluorescence microscopy, we further confirmed that IR-61 preferentially accumulated in F4/80^+^ cells from the stromal vascular fraction (SVF) (Fig. 1f). Furthermore, we found that M1-like peritoneal macrophages (PMs) showed a greater uptake of IR-61 than did M2-like or unstimulated primary PMs (Supplementary Fig. 3b). Consistently, M1-like ATMs (F4/80^+^CD11C^+^) internalized more IR-61 than non-M1-like ATMs (F4/80^+^CD11C^−^) (Supplementary Fig. 3c). Taken together, these results indicated that IR-61 preferentially accumulated in ATMs. The characteristics of IR-61 targeting ATMs involved its physico-chemical properties, the advantages of intraperitoneal injection, and the high phagocytic properties of macrophages (see Discussion below).

To confirm the subcellular localization of IR-61, we stained macrophages with mitochondria-specific fluorescence probes (Mito-Tracker Green) and found that IR-61 exclusively accumulated in the mitochondria of (Fig. 1g). Lipophilic cationic property of NIR fluorescent dye is necessary for targeting mitochondria, which explains mitochondria-preferential accumulation of IR-61^32–34^. Concurrently, we confirmed that IR-61 was distributed particularly in the mitochondria of ATMs (Supplementary Fig. 3d). Multiple pathways mediated the internalization of these particles and include phagocytosis, micropinocytosis, and clathrin-mediated and caveolae-mediated endocytosis. To determine the specific endocytotic pathway involved in IR-61 internalization, we performed a series of IR-61 uptake assays in the presence of biochemical inhibitors to block specific pathways. We found that macrophages pretreated with amiloride (Amil, micropinocytosis inhibitor), chlorpromazine (Chlor, clathrin-mediated endocytosis inhibitor), and methyl-β-cyclodextrin (MβCD, caveolae-mediated endocytosis inhibitor) all inhibited IR-61 uptake (Fig. 1h–j). These results indicated that IR-61 was internalized via macropinocytosis and clathrin-dependent or caveolae-dependent pathways.

### IR-61 suppresses M1 macrophage activation through mitochondria

**Fig. 2:**
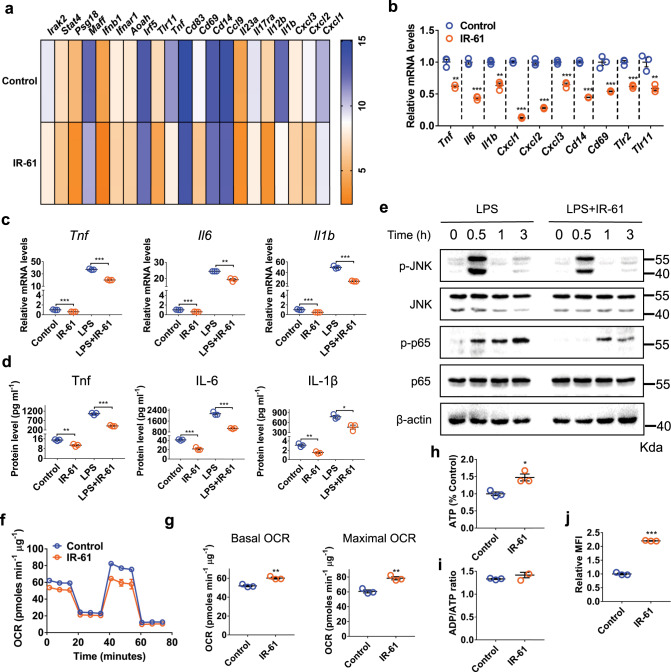
IR-61 suppresses M1 macrophage activation through mitochondria. **a** The RAW264.7 cell line was treated with an additional 10 μM IR-61 for 24 h, and then the gene microarray was analyzed. mRNA levels of pro-inflammatory molecules in IR-61-treated macrophages were presented as fold changes relative to those of untreated macrophages. **b** IR-61 reduced the gene expression of pro-inflammatory molecules as determined by qPCR. **c** IR-61 suppressed LPS-induced gene expression. BMDMs were treated with or without additional 10 μM IR-61 for 24 h, and then treated with or without 100 ng ml^-1^ LPS for another 24 h. **d** Cytokine secretion level was measured by ELISA in BMDMs treated as in (**c**). **e** BMDMs were treated with or without additional 10 μM IR-61 for 24 h prior to LPS (100 ng ml^-1^) treatment for 0.5, 1, or 3 h and then subjected to western blotting. β-actin was used as a loading control. **f, g** Oxygen consumption rates (OCR) and mitochondrial function of the BMDMs treated with IR-61 or vehicle control for 24 h. **h** ATP levels in BMDMs. **i** ADP/ATP ratio in BMDMs. **(j)** Mitochondrial membrane potential of BMDMs. Data are representative of three independent experiments. Results are presented as the mean ± SEM (*p < 0.05, **p < 0.01, ***p < 0.001, *n* = 3; two-sided Student’s *t*-test). Exact *p*-values are given in the Source Data file. Source data are provided as a Source Data file.

To investigate the role of IR-61 in macrophage activation, we treated RAW264.7 cells with IR-61 and performed gene microarray analysis. We demonstrated that IR-61 reduced the expression of several pro-inflammatory factors, including cytokines (*Tnf* and *Il1b*), chemokines (*Cxcl1, Cxcl2* and *Cxcl3*), and cell surface receptors (*Cd14, Cd69, Tlr2* and *Tlr11*) (Fig. 2a). The inhibitory effect of IR-61 on the pro-inflammatory genes described above was also demonstrated by quantitative RT-PCR in Fig. 2b. We next explored whether IR-61 affected M1 activation. Our results showed that IR-61 reduced LPS-induced mRNA and protein secretion of pro-inflammatory cytokines including *Tnf, Il6*, and *Il1b* in macrophages (Fig. 2c, d). We also determined the effect of IR-61 on IL-4-induced M2 activation. The result showed that IR-61 induced the expression of a panel of signature genes: *Arg1, Mrc1, Fizz1*, and *Ym1*, which indicated that IR-61 promoted M2 activation of macrophages (Supplementary Fig. 4a). Moreover, IR-61 did not induce cytotoxicity or cell apoptosis/necrosis at any concentration tested, which excluded cell death as an explanation for the effects observed on inflammatory-related gene expression (Supplementary Fig. 4b). Further, we found that IR-61 treatment reduced LPS-stimulated phosphorylation of p65 and JNK (Fig. 2e). Taken together, these data suggested that IR-61 suppressed the pro-inflammatory activation of macrophages.

Considering the colocation of IR-61 with mitochondria and the regulatory effect of oxidative phosphorylation on macrophage activation, we speculated that IR-61 might modulate mitochondria-dependent macrophage activation. As expected, IR-61 treatment significantly increased the basal and maximal respiratory capacity of macrophages (Fig. 2f, g). Moreover, IR-61-treated cells generated more mitochondrial ATP and displayed higher mitochondrial membrane potential (Fig. 2h, j). The ADP/ATP ratio of IR-61-treated BMDMs was comparable to that of the vehicle control BMDMs (Fig. 2i). In addition, IR-61 had no interference in ATP assay and assessment of mitochondrial membrane potential (Supplementary Fig. 5a, b). Furthermore, we pretreated macrophages with carbonyl-cyanide-p-trifluoro-methoxy-phenylhydrazone (FCCP), a potent uncoupler of mitochondrial oxidative phosphorylation. We demonstrated that the IR-61-induced reduction in pro-inflammatory cytokine in macrophages was prevented by FCCP (Supplementary Fig. 6). These findings indicated that IR-61 prevented macrophage M1 activation depending on mitochondrial function.

### IR-61 regulates mitochondrial complex content and activity

**Fig. 3:**
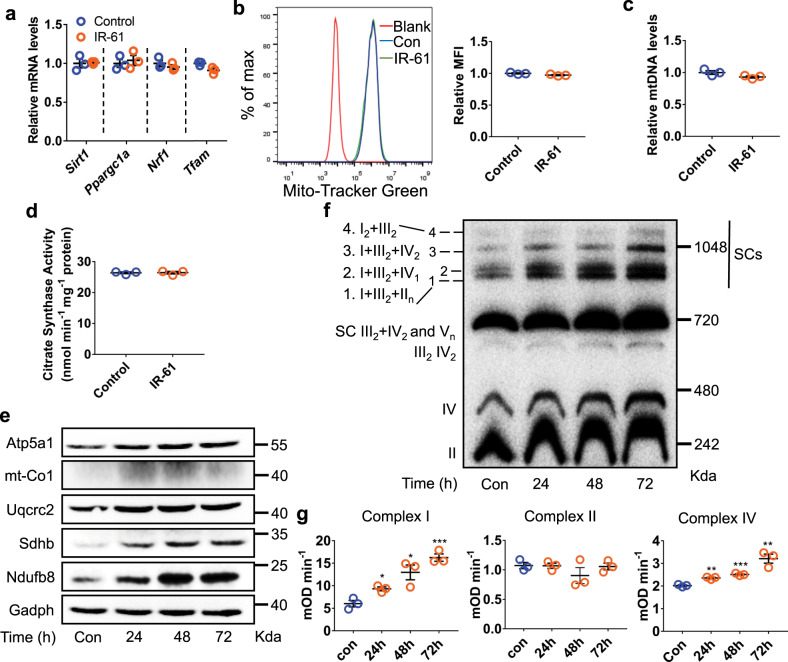
IR-61 regulates mitochondrial complex content and activity. **a** Relative mRNA levels of genes associated with mitochondrial biogenesis in the BMDMs. **b** BMDMs were treated with IR-61 or vehicle control for 24 h, and stained with MitoTracker Green, and then the mitochondrial content was determined by flow cytometry. **c** DNA levels of mitochondrial (mt) gene encoding cytochrome b, which were normalized to those of the nuclear gene Ppia in BMDMs treated with IR-61 or vehicle control for 24 h. **d** Citrate synthase activity of BMDMs treated with IR-61 or vehicle for 24 h. **e** Immunoblots of the respiratory chain subunits in the BMDMs treated with IR-61 for 24, 48, or 72 h. Gadph was used as the loading control. **f** BN-PAGE immunoblots of mitochondrial complex and supercomplex content in the BMDMs treated with IR-61 for 24, 48, or 72 h. The supercomplexes were visualized by antibodies against subunits of complex I (Ndufa9), complex II (Sdha), complex III (Uqcrc2), complex IV (Cox4a) and complex V (Atp5a1). **g** Complex I, II, and IV activity was measured by ELISA in the BMDMs treated with IR-61 for 24, 48, or 72 h. Activity is expressed as the change in absorbance per minute (mOD min^−1^) per 200 μg of cell lysate. Data are representative of three independent experiments. Results are presented as the mean ± SEM (*p < 0.05, **p < 0.01, ***p < 0.001, *n* = 3; two-sided Student’s *t*-test). Exact *p*-values are given in the Source Data file. Source data are provided as a Source Data file.

We further explored the molecular mechanisms of IR-61 in potentiating mitochondrial oxidative phosphorylation. To analyze whether IR-61 increased mitochondrial biogenesis, we measured the mRNA levels of the biogenesis-related factors *Sirt1*, *Ppargc1a*, *Nrf1*, and *Tfam* in the macrophages. These data showed that the expression levels of these genes in IR-61-treated BMDMs were comparable to those of the vehicle control macrophages (Fig. 3a). Moreover, the IR-61 treatment did not affect mitochondrial mass significantly according to the results of staining with MitoTracker Green (Fig. 3b), and IR-61 did not interfere with MitoTracker Green (Supplementary Fig. 5c). The mitochondrial DNA of IR-61-treated macrophages had no significant change after normalizing to nuclear DNA compared with vehicle-treated macrophages (Fig. 3c). In addition, we also assessed citrate synthase activity and found that IR-61 had no effect on it (Fig. 3d). Therefore, mitochondrial biogenesis was not responsible for the IR-61-regulated mitochondrial function. Moreover, AMPK activity was not affected by IR-61 and we did not find any obvious change in the genes associated with fatty acid oxidation (Supplementary Fig. 7a, b). ATP production in mitochondria relies on the electron transport chain and oxidative phosphorylation. This process involves five multisubunit respiratory complexes (I–V). Therefore, we analyzed the transcription and protein levels of specific components of these mitochondrial complexes by qPCR and immunoblotting. We found that the mRNA and protein levels of Ndufb8 (complex I), Sdhb (complex II), Uqcrc2 (complex III), mt-Co1 (complex IV), and Atp5a1 (complex V) were markedly increased in response to IR-61 treatment (Supplementary Fig. 7c and Fig. 3e). Supercomplex assembly in macrophages was assessed by blue native polyacrylamide gel electrophoresis (BN-PAGE). These results showed that the overall content of supercomplex was increased after IR-61 treatment in BMDMs, as evidenced in immunoblots using an OXPHOS antibody cocktail (Fig. 3f). Further, we also demonstrated that IR-61 induced the activity of complex I and IV at 24, 48, and 72 h after IR-61 treatment (Fig. 3g). Altogether, these results indicated that IR-61 potentiated mitochondrial function by enhancing the content and activity of the mitochondrial complexes.

### IR-61 stimulates mitochondrial function through ROS-Akt-Acly signaling

**Fig. 4:**
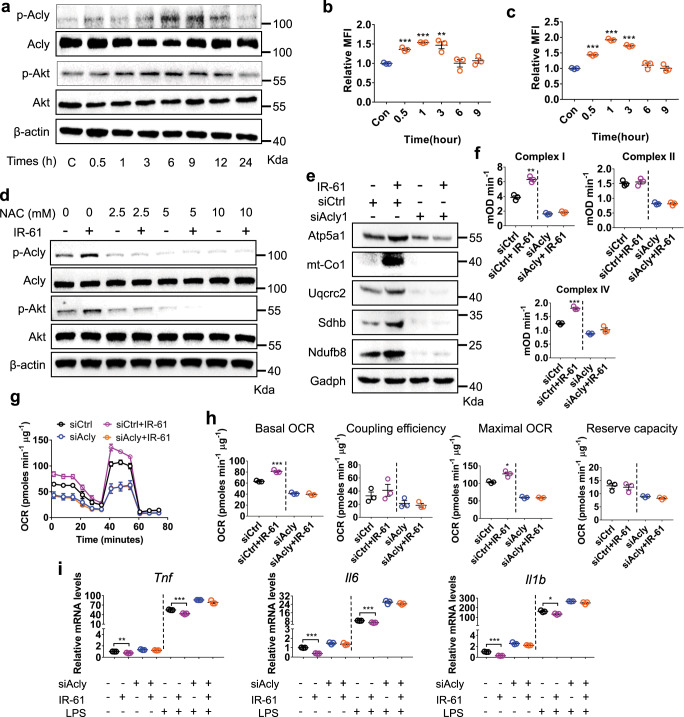
IR-61 stimulates mitochondrial function through ROS-Akt-Acly signaling. **a** Immunoblots of Acly, p-Acly, Akt, and p-Akt in whole-cell lysates from the IR-61-treated BMDMs for the indicated times. β-Actin was used as the loading control. **b** Mitochondrial superoxide variation at 0–9 h after IR-61 treatment (*n* = 3). **c** Intracellular ROS variation at 0–9 h after IR-61 treatment (*n* = 3). **d** Immunoblots of Acly, p-Acly, Akt, and p-Akt in whole-cell lysates from the BMDMs treated with the vehicle control, NAC (2.5, 5, and 10 mM), or IR-61 with or without NAC for 6 h. β-actin was used as the loading control. **e** Immunoblots of respiratory chain subunits in whole-cell lysates from the IR-61-treated BMDMs for 72 h after transfection with siRNA control (siCtrl) or Acly siRNA1 (siAcly1). Gadph was used as the loading control. **f** Complex I, II, and IV activity was measured by ELISA of the BMDMs treated with IR-61 for 72 h after transfection with siCtrl or siAcly. **g, h** OCR and mitochondrial function of the BMDMs treated with siCtrl or siAcly for 48 h and then treated with IR-61 or vehicle control for another 24 h. **i** Pro-inflammatory mRNA levels of the IR-61-treated BMDMs transfected with siCtrl or siAcly for 48 h, and then treated with LPS for another 24 h. Data are representative of three independent experiments. Results are presented as the mean ± SEM (*p < 0.05, **p < 0.01, ***p < 0.001, *n* = 3; two-sided Student’s *t*-test). Exact *p*-values are given in the Source Data file. Source data are provided as a Source Data file.

Next, we explored the upstream components that mediated the increased content and activity of mitochondrial complexes in IR-61-treated macrophages. We found that IR-61 promoted the phosphorylation of Akt and Acly (Fig. 4a). Since both Akt and Pka could regulate phosphorylation of Acly at S^455^, Akt inhibitor and Pka inhibitor were used to determine which kinase was involved in IR-61-induced phosphorylation of Acly. We found that IR-61 promoted phosphorylation of Acly at S^455^ through Akt (Supplementary Fig. 8a, b). Moreover, IR-61 transiently stimulated cytosolic and mitochondrial ROS levels (Fig. 4b, c) and 10 μM IR-61 make the mitoROS production reached its peak value in BMDMs (Supplementary Fig. 9a). In addition, IR-61 did not interfere with Mitosox and DCFH-DA (Supplementary Fig. 5d, e). ROS were responsible for IR-61 enhancement of Akt/Acly phosphorylation when anti-ROS agents, including N-acetyl-cysteine (NAC), SS-31, and MnTMPYP, were used to treat macrophages (Fig. 4d and Supplementary Fig. 9b, c). Consistently, IR-61 promoted Akt/Acly phosphorylation and provoked a transient increase of mitochondrial and cytosolic superoxide in ATMs (Supplementary Fig. 9d, e). Moreover, NAC, SS-31, and MnTMPYP treatment all abrogated the effect of IR-61 on promoting the phosphorylation of Akt and Acly in ATMs (Supplementary Fig. 9e–g). Considering the crucial role of Acly in regulating mitochondria complexes, we explored whether IR-61 depended on Acly to regulate mitochondrial function and inflammation in macrophages. To avoid the off-target effect of siRNA knockdown, we screened two efficient siRNAs to silence Acly expression and found that both siRNAs prevented IR-61 from promoting expression of the mitochondrial complexes (Fig. 4e, Supplementary Fig. 10a, b). Moreover, Acly silencing also abrogated the function of IR-61 increasing activity of the mitochondrial complexes (Fig. 4f). Consistently, we found that IR-61 increased mitochondrial complex content in ATMs, and Acly was responsible for IR-61 regulation of mitochondrial complex content when Acly inhibitor (BMS-303141) was used in vivo (Supplementary Fig. 10c). Moreover, the IR-61-stimulated oxygen consumption rate was diminished by Acly knockdown (Fig. 4g, h). Since Acly knockdown and BMS-303141 treatment reduced the content of mitochondrial complex in macrophages, we conducted Annexin/PI assay to determine whether the mitochondrial metabolism decline was due to Acly knockdown or BMS-303141 administration impairing cell viability significantly. As expected, neither of them impaired viability of macrophages significantly in the presence or absence of IR-61 (Supplementary Fig. 10d, e). Next, we measured the content of cardiolipin, a phospholipid regulated by Acly and was vital for the stability of mitochondrial complex^35–37^. IR-61 increased the content of mitochondrial cardiolipin and Acly knockdown abrogated the effect of IR-61 on cardiolipin (Supplementary Fig. 10f). Moreover, BMS-303141 suppressed cardiolipin production by inhibiting the activity of Acly in macrophages (Supplementary Fig. 10f). Furthermore, Acly downregulation by siRNA could rescue the inhibitory effect of IR-61 on the expression of pro-inflammatory cytokines in macrophages (Fig. 4i). Altogether, these results indicated that IR-61 enhanced the content and activity of the mitochondrial complexes through the ROS-Akt-Acly pathway and thus inhibited macrophage M1 activation.

### IR-61 suppresses chronic inflammation in vivo

**Fig. 5:**
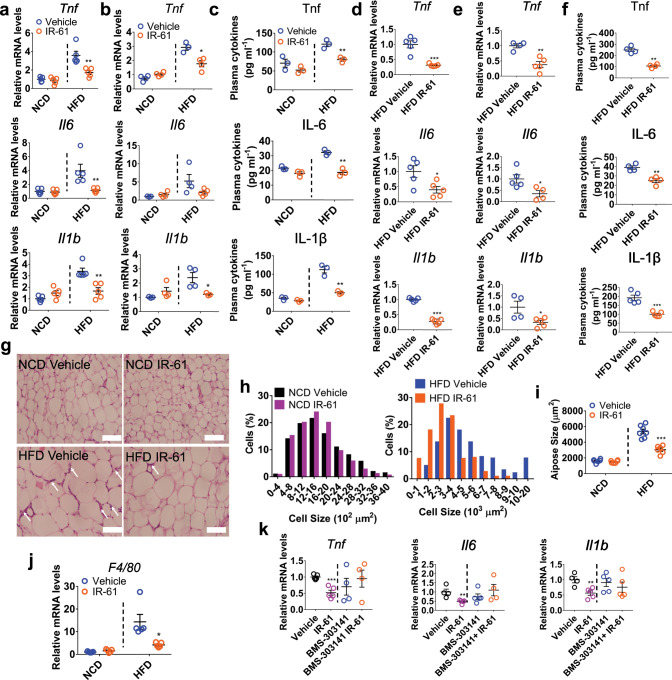
IR-61 suppresses chronic inflammation in vivo. **a–c** Relative mRNA levels of the inflammatory cytokines in the epi WAT (**a**) and liver (**b**), plasma concentrations of cytokines (**c**) of NCD-fed and HFD-fed mice treated preventatively with IR-61 or the vehicle control. **d**–**f** Relative mRNA levels of the inflammatory cytokines in epi WAT (**d**) and liver (**e**), plasma concentrations of the cytokines (**f**) of the mice with established obesity treated with IR-61 or vehicle control. **g** Representative images of H&E stained sections of VAT from NCD-fed or HFD-fed mice treated preventatively with IR-61 or vehicle control (Scale bars, 100 μm). White arrows show crown-like structures. **h, i** Adipocyte size distribution (left panel) and the average size of adipocytes (right panel) were assessed on H&E-stained slices in (**g**). **j** Relative mRNA levels of the macrophage marker in the epi WAT of NCD-fed and HFD-fed mice treated with IR-61 or vehicle control. **k** Relative mRNA levels of the inflammatory cytokines in epi WAT of the mice with established obesity treated with vehicle, IR-61, BMS-303141, and BMS-303141+IR-61. Sample sizes are (**a**) *n* = 5/5/5/5 mice, (**b**) *Tnf n* = 4/4/3/4 mice, *Il6 n* = 3/4/4/5 mice, *Il1b n* = 3/4/4/3 mice, (**c**) *n* = 3/3 mice, (**d**) *n* = 5/5 mice, (**e**) *Tnf n* = 4/5 mice, *Il6 n* = 5/4 mice, *Il1b n* = 4/5 mice, (**f**) Tnf *n* = 4/4 mice, IL-6 *n* = 4/4 mice, IL-1β *n* = 5/5 mice, (**g-i**) *n* = 6/6/7/6 mice, (**j**) *n* = 5/5/5/5 mice, (**k**) *Tnf n* = 5/5/4/4 mice, *Il6 n* = 4/5/5/4 mice, *Il1b n* = 4/5/5/5 mice. Data are presented as the mean ± SEM (*p < 0.05, **p < 0.01, ***p < 0.001; two-sided Student’s *t*-test). Exact *p*-values are given in the Source Data file. Source data are provided as a Source Data file.

To determine whether IR-61 could modulate obesity-associated chronic inflammation, we fed age-matched C57BL/6 mice a normal chow diet (NCD) or a high-fat diet (HFD) and injected the mice with IR-61 or a vehicle control via concurrent intraperitoneal administration. These results showed that IR-61 significantly inhibited the expression of pro-inflammatory factors in visceral fat and liver as well as the levels of pro-inflammatory cytokines in plasma of obese mice, but not in lean mice (Fig. 5a–c). In the established obese mice, the expression of pro-inflammatory genes in adipose tissue, liver, and blood was also markedly reduced by IR-61 treatment (Fig. 5d–f). H&E staining of the adipose tissue showed that IR-61 treatment significantly reduced the size of the adipocytes (Fig. 5g). Quantification of adipocyte size revealed that in HFD-fed mice, IR-61 caused a greater frequency of small adipocytes and a lower frequency of middle to large adipocytes, resulting in a 30% reduction in area of epi WAT adipocytes (Fig. 5h, i). However, IR-61 had no effect on NCD-fed mice (Fig. 5h, i). Moreover, the crown-like structures and infiltration of macrophages into the adipose tissues that were induced by the HFD decreased markedly in the IR-61-treated mice (Fig. 5g, j). As our findings revealed that IR-61 regulated Acly-dependent macrophage activation in vitro, we further explored whether the IR-61-Acly axis was functional in ATM activation in vivo. We treated the HFD-induced obese mice with IR-61 and BMS-303141 via intraperitoneal administration. The dose of the inhibitor we used could effectively inhibit Acly phosphorylation in ATMs (Supplementary Fig. 11). Moreover, we demonstrated that the anti-inflammatory effect of IR-61 on visceral adipose tissues was abrogated by the Acly inhibitor (Fig. 5k).

### IR-61 triggers weight loss and improves insulin sensitivity in obese mice

**Fig. 6:**
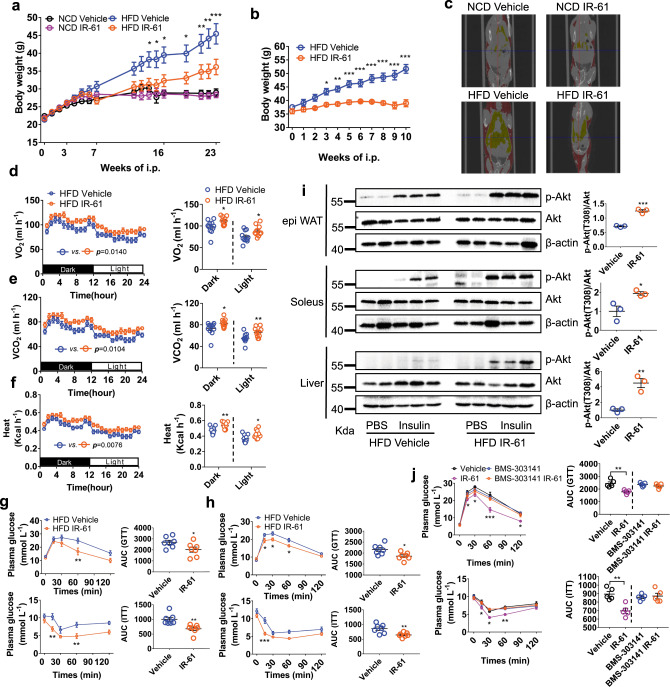
IR-61 triggers weight loss and improves insulin sensitivity in obese mice. **a** Body weight of mice on NCD or HFD concurrently treated with IR-61 or vehicle control. **b** Body weight of the mice with established obesity treated with IR-61 or vehicle control. **c** Fat distribution was detected by μCT. Yellow indicates visceral fat and red indicates subcutaneous fat. **d–f** VO_2_, VCO_2_, and heat production over a 24-h period of obese mice treated with IR-61 or vehicle control for 2 weeks were obtained by indirect calorimetry. The average VO_2_, VCO_2_, and heat production of each mouse represented as scatterplots. **g** GTT and ITT were performed in HFD-fed mice treated preventatively with IR-61 or vehicle control for 15 weeks. Area under the curve (AUC) was determined for each individual animal for GTT and ITT. **h** GTT and ITT on established obesity mice treated with IR-61 or vehicle control for 6 weeks. The AUC was determined for each individual animal for GTT and ITT. **i** Acute insulin signaling in the epi WAT, soleus and liver of mice on HFD. **j** GTT and ITT on the established obesity mice treated with vehicle, IR-61, BMS-303141, and BMS-303141+IR-61 for 2 weeks. The AUC was determined for each individual animal for GTT and ITT. Sample sizes are (**a**) *n* = 7/7/7/7 mice, (**b**) *n* = 7/7 mice, (**d**–**f**) *n* = 11/11 mice, (**g, h**) *n* = 7/7 mice, (**i**) representative western blot of *n* = 2 vehicle + PBS, *n* = 3 vehicle + insulin, *n* = 2 IR-61 + PBS, *n* = 3 IR-61 + insulin mice and quantification of p-Akt/Akt in *n* = 3 vehicle + insulin and IR-61 + insulin mice, (**j**) *n* = 5/5/5/5 mice. Data are presented as the mean ± SEM (*p < 0.05, **p < 0.01, ***p < 0.001; two-way ANOVA with Bonferroni post hoc test; longitudinal data in panels (**a, b, d–h, j**)) or two-sided Student’s *t*-test (plot graphs in panels (**d–j**)). Exact *p*-values are given in the Source Data file. Source data are provided as a Source Data file.

Considering of the properties of IR-61 targeting ATMs and suppressing macrophage M1 activation, we speculated that IR-61 might regulate the progression of obesity and obesity-related diseases. To test this idea, we simultaneously monitored the body weight of mice fed a HFD or a NCD and treated with IR-61 or vehicle control. Interestingly, we found that IR-61 could prevent weight gain in mice fed a HFD (Fig. 6a). However, in the NCD-fed group, the body weight of the IR-61-treated mice was comparable to that of the vehicle control mice (Fig. 6a). In parallel, IR-61 could significantly reduce the weight of visceral fats in mice fed a HFD but not in those fed a NCD (Supplementary Fig. 12a, b). In the established obese mice, the IR-61 treatment could also reduce the weight of the whole mouse body and visceral fat (Fig. 6b and Supplementary Fig. 12c). Analytical microCT (μCT) imaging revealed a decrease in fat mass throughout the bodies of the IR-61-treated obese mice (Fig. 6c). Furthermore, IR-61 treatment reduced the levels of plasma triglyceride and cholesterol in the mice fed a HFD but not the mice fed a NCD (Supplementary Fig. 12d, e). In addition, IR-61 had no effect on the weight of fat-free organs in the mice fed with NCD, nor did it affect the weight of fat-free organs except liver in the mice fed a HFD (Supplementary Fig. 12a–c). More importantly, IR-61 treatment caused no pathological, blood routine and serum biochemical parameters abnormalities in NCD-fed mice, which indicated that IR-61 had conferred no obvious toxicity in the animals (Supplementary Figs. 13 and  14). As IR-61 had no effect on food intake and intestinal lipid absorption (Supplementary Fig. 15a, b), we speculated that weight loss was the result of enhanced energy expenditure. As expected, 2-week treatment of obese mice induced by 12-week HFD with IR-61 resulted in higher oxygen consumption rate (VO_2_), carbon dioxide production rate (VCO_2_), and heat production after normalization of body mass or adjustment for covariate body mass using regression-based analysis of covariance (ANCOVA) (Supplementary Fig. 16a–c). Considering lean and fat tissue expend energy at different rates^38^, the optimal analytical method for measuring metabolic rate of mice with difference in adiposity is adjusted for covariate lean mass and fat mass using ANCOVA^38,39^. As we did not collect lean and fat mass data, we further analyzed the metabolic data of each mouse without normalization to body mass to facilitate a comprehensive evaluation of the effect of IR-61 on energy expenditure as described in the published literature^40^ (Fig. 6d–f). Moreover, IR-61 had no effect on NCD-fed mice (Supplementary Fig. 16d–f) and we found no significant difference in total activity of both NCD-fed and HFD-fed mice (Supplementary Fig. 16g, h). In addition, energy expenditure and total activity of HFD-fed mice treated with IR-61 for 1 day were comparable to those of the vehicle control mice (Supplementary Fig. 17a–d), indicating that IR-61 had no direct effect on energy expenditure in obese mice. Since infiltration of pro-inflammatory M1 macrophages inhibits beige adipogenesis in obesity^41–43^, we asked whether IR-61 promoted the browning/beiging of WAT or enhanced brown adipose tissue (BAT) activity. Our results showed that IR-61 promoted Ucp1 expression of inguinal white adipose tissue (iWAT) in obese mice (Supplementary Fig. 17e). Moreover, IR-61-treated obese mice exhibited higher expression of brown/beige fat thermogenic genes in epi WAT, including *Ppargc1a*, *Ucp1*, *Prdm16*, *Cd137*, *Tbx1*, and *Cd40* (Supplementary Fig. 17f). But the genes associated with activity of BAT, including *Ppargc1a*, *Ucp1*, *Cox7a1*, *Cox8b*, *Cidea*, *Tfam*, and *Nrf1* were not affected by IR-61 (Supplementary Fig. 17g). Taken together, these results demonstrated that IR-61 reversed the inhibition of inflammation on beige adipogenesis, increased energy expenditure and prevented weight gain of HFD-fed mice.

Obesity is often associated with insulin resistance. Therefore, we further assessed the regulatory effect of IR-61 on insulin sensitivity. For the glucose tolerance tests (GTT) and insulin tolerance tests (ITT), we observed that mice treated with IR-61 concurrently with a HFD or after obesity establishment had improved both glucose tolerance and insulin tolerance, which revealed that IR-61 treatment significantly improved insulin sensitivity in the obese mice (Fig. 6g, h). However, IR-61 treatment had no effect on glucose tolerance in NCD-fed mice (Supplementary Fig. 18a, b). Finally, we examined acute insulin signal in mice on a HFD. We demonstrated that insulin-stimulated Akt (Thr308) phosphorylation was increased by 1.7-fold, 1.9-fold, and 4.5-fold in epi WAT, soleus, and liver, respectively, in the IR-61-treated mice (Fig. 6i). In summary, we found that IR-61 treatment could enhance insulin signal transduction and alleviate insulin resistance in the obese mice. Finally, we showed that the effect of IR-61 on improved insulin resistance was reversed when Acly was inhibited in vivo by BMS-330241 (Fig. 6j).

### IR-61 inhibits inflammation prior to body weight change

**Fig. 7:**
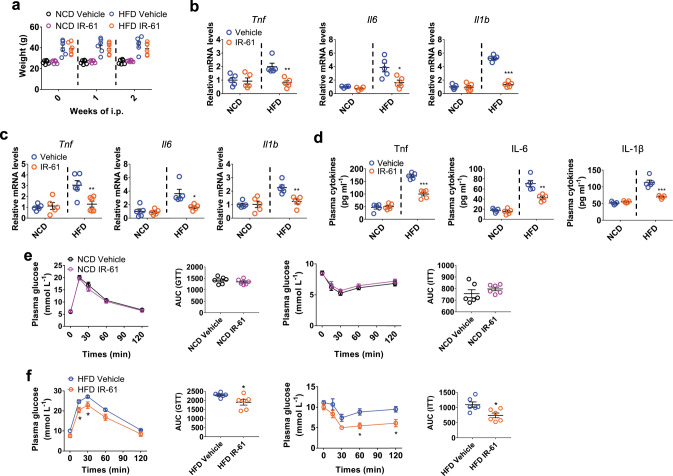
IR-61 inhibits inflammation before body weight differences occur. Mice were fed with HFD for 12 weeks to induce obesity. **a** Body weight of mice on NCD or HFD concurrently treated with IR-61 or vehicle control. **b, c** Expression of the inflammatory cytokines in the epi WAT and liver of NCD-fed and HFD-fed mice treated with IR-61 or the vehicle control. **d** Plasma concentrations of cytokines in NCD-fed or HFD-fed mice treated preventatively with IR-61 or vehicle control. **e, f** GTT and ITT on NCD-fed or HFD-fed mice treated with IR-61 or vehicle control for 2 weeks. The AUC was determined for each individual animal for GTT and ITT. Sample sizes are (**a**) *n* = 6/6/6/6 mice, (**b**) *Tnf n* = 5/5/5/5 mice, *Il6 n* = 4/4/5/5 mice, *Il1b n* = 5/5/5/5 mice, (**c**) *Tnf n* = 5/5/6/6 mice, *Il6 n* = 5/5/5/5 mice, *Il1b n* = 5/5/5/5 mice, (**d**) *n* = 5/5/5/5 mice, (**e**) GTT *n* = 6/6 mice, ITT *n* = 6/6 mice, (**f**) GTT *n* = 6/6 mice, ITT *n* = 6/6 mice. Results are presented as the mean ± SEM (*p < 0.05, **p < 0.01, ***p < 0.001; two-way ANOVA with Bonferroni post hoc test (longitudinal data in panels (**e, f**)) and two-sided Student’s *t*-test (plot graphs in panels (**a–f**))). Exact *p*-values are given in the Source Data file. Source data are provided as a Source Data file.

In order to determine whether the reduction in systemic and local inflammation was in causative relation with IR-61 treatment or whether it was merely a result of weight loss, we characterized HFD-fed mice at 2 weeks after IR-61 treatment, when there was no difference in body weight (Fig. 7a). At this time, IR-61-treated obese mice showed a significant reduction in the expression of pro-inflammatory factors in visceral fat and liver as well as the levels of pro-inflammatory cytokines in plasma (Fig. 7b–d). Inflammation can directly cause insulin resistance^44,45^. To illuminate whether the improved insulin sensitivity observed in IR-61-treated obese mice was linked to the direct anti-inflammatory effect of IR-61 in vivo, or the reduction of inflammation caused by weight loss, we performed GTT/ITT prior to body weight change. We found that short-time treatment of IR-61 increased insulin sensitivity in obese mice, but not in lean mice (Fig. 7e, f). Together, these experiments indicated that IR-61 treatment alleviated HFD-induced tissue inflammation and insulin resistance prior to weight change, implying the effects observed after IR-61 treatment were of primary cause and not secondary to the weight loss.

### IR-61 treatment reverses hepatic steatosis in mice

**Fig. 8:**
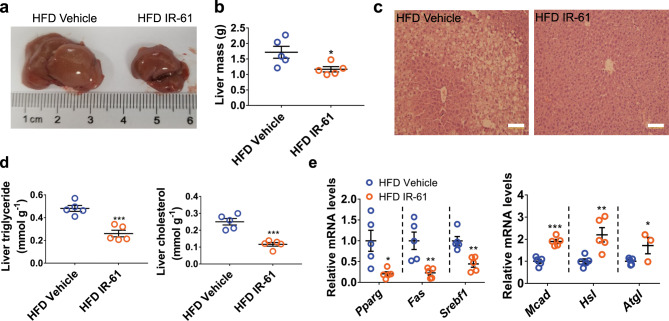
IR-61 treatment reverses hepatic steatosis. **a** Representative microscopic pictures of the livers from the established obesity mice treated with IR-61 or vehicle control. **b** Total liver weight of the two groups of mice**. c** Representative images of H&E stained livers in the indicated groups (scale bars, 100 μm). **d** Liver triglyceride and cholesterol content of the two groups of mice. **e** mRNA abundance of lipid synthesis genes and lipolysis genes in the livers of mice in each treatment group. Sample sizes are (**b**) *n* = 5/5 mice, (**c**) *n* = 5/5 mice, (**d**) *n* = 5/5 mice, (**e**) *Mcad n* = 5/5 mice, *Hsl n* = 5/5 mice, *Atgl n* = 5/3 mice. Data are presented as the mean ± SEM (*p < 0.05, **p < 0.01, ***p < 0.001; two-sided Student’s *t*-test). Exact *p*-values are given in the Source Data file. Source data are provided as a Source Data file.

It is known that obesity is often accompanied by hepatic steatosis. Therefore, we treated the established obese mice with IR-61 or vehicle control for 10 weeks and then examined liver pathology. The hepatomegaly observed in the HFD-fed mice was not apparent in the IR-61-treated group, and the liver weight was reduced by approximately 30% (Fig. 8a, b). Moreover, H&E staining revealed that severe lipid deposition in the liver of HFD-fed mice was markedly alleviated by IR-61 (Fig. 8c). The triglyceride and cholesterol content in the liver was more than 40% lower in the IR-61-treated mice than in the control mice (Fig. 8d). Compared to control mice, the IR-61-treated mice had concurrently lower expression of several lipogenic genes, including *Pparg*, *Fas*, and *Srebf1*, as well as higher expression of lipolysis genes, including *Mcad*, *Hsl*, and *Atgl* (Fig. 8e). Therefore, IR-61 treatment reversed obesity-induced hepatic steatosis in the mice.

## Discussion

Anti-inflammation is an important strategy in the treatment of obesity and obesity-related metabolic syndromes^46,47^. However, traditional anti-inflammatory agents have poor targeting ability and cause severe side effects^48^. ATMs have been considered as therapeutic targets for their crucial role in obesity-related inflammation and metabolic disorders, but are still limited by problems with traditional macrophage-targeting nanomedicines, such as high cost and instability^25,26,49^. Many studies have identified that complex changes in mitochondrial metabolism are characteristics and controllers of macrophage activation^50,51^. However, current metabolic regulatory agents have been limited by off-target effect, and no study has actually shown a direct link between the oxidative function of ATMs and the systemic inflammation and metabolic disorders due to the lack of an experimental oxidative function-related gene modification that can restrict ATMs^24,52^. Therefore, administration of multifunctional agents targeting ATMs while simultaneously enhancing oxidative phosphorylation would be of benefit for precise metabolite-targeting therapy. In the present study, we characterized a small-molecule dye, IR-61, which preferentially accumulates in ATMs via i.p. injection and suppresses local and systemic inflammation to treat obesity and obesity-related disorders. Mechanistic studies showed that IR-61 targets the mitochondria of ATMs and enhances oxidative phosphorylation by increasing the expression and activity of mitochondrial complexes, thereby preventing ATMs from diverting to the M1 pro-inflammatory phenotype under obesity.

The property of IR-61 targeting the oxidative phosphorylation of ATMs is the highlight in this study. The precise mechanism by which IR-61 targets ATMs might originate from the drug delivery route and its physico-chemical property. Peritoneal administration enables the direct physical delivery of the administered dose to visceral adipose tissue, and the fewer blood vessels and slow flow in adipose tissue are expected to cause the administered dose to stay longer in the visceral adipose tissue^53^. IR-61 is a small molecule with lipophilic cationic property and transmission electron microscopy (TEM) revealed that IR-61 formed self-assembly spherical nanoparticles with a diameter of 90 ± 5 nm in an aqueous solution (Supplementary Fig. 2a). One of the basic and special properties of many lipophilic agents is their bioaccumulation ability in adipose tissue^54^. Moreover, based on the current understanding of passive targeting of macrophages, physico-chemical properties of particles, such as shape, charge, and hydrophilic/lipophilic character, are closely related to its interactions and subsequent uptake with macrophages: (i) spherical particles are more efficiently internalized by macrophages compared with other geometric shapes^55–57^, (ii) positively charged particles exhibit higher targeting efficiency due to their superior interaction with negatively charged cell membranes^58,59^, and (iii) lipophilic particles were taken up more efficiency than particles with higher hydrophilicity^60,61^. Thus, IR-61 represents an ideal ATMs-targeting small molecule with intrinsic structure-inherent targeting properties.

Mitochondria generate ATP through the electron transport chain and ATP synthase, a multi-protein system organized by five multisubunit respiratory complexes (I–V) embedded within the inner membrane of mitochondria. Defects in any of the complexes impair mitochondrial function and trigger other biological functions in macrophages^62^. For example, mice deficient in NDUFS4, a subunit of complex I, exhibit diminished mitochondrial function and enhanced M1 polarization, indicating an important role for respiratory complexes in macrophage polarization^63^. Our current study also demonstrates that IR-61 regulates mitochondrial complex-dependent oxidative phosphorylation and subsequent macrophage activation. Acly is a cytosolic enzyme that is primarily responsible for catalyzing the reaction of mitochondria-derived citrate into oxaloacetate and acetyl-CoA^64^. Here, we demonstrate that IR-61 promotes phosphorylation of Acly through the ROS-Akt pathway, thereby potentiating macrophage oxidative phosphorylation by increasing the content and activity of mitochondrial complexes. The regulatory effect of Acly on mitochondrial function has also been demonstrated in skeletal muscle cells^35^. However, it should be noted that Acly knockdown reduces the content and activity of mitochondrial complexes, which implies that Acly may be not the direct requirement for the IR-61 function.

**Fig. 9:**
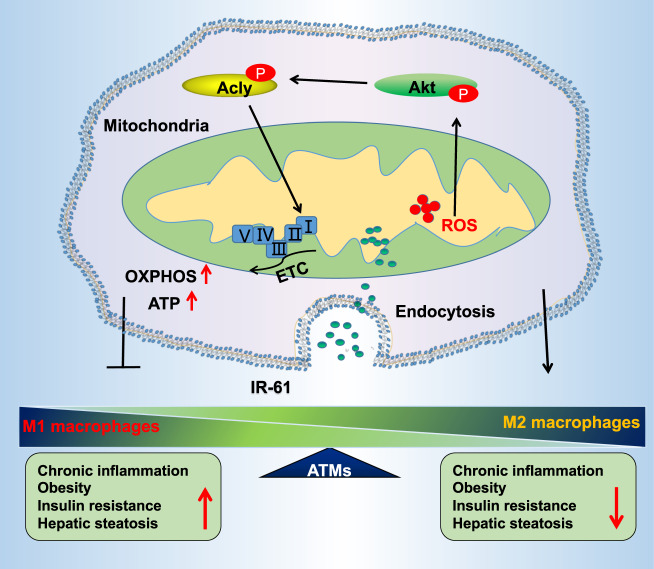
Summary of ATMs mitochondria-targeting IR-61. IR-61 selectively targets ATMs via the endocytosis pathway and preferentially accumulates in the mitochondria. IR-61 induces transiently increased mitochondrial ROS and promotes phosphorylation of Akt and Acly, which further potentiates mitochondrial oxidative phosphorylation by increasing the content and activity of the mitochondrial complexes to inhibit pro-inflammatory macrophage (M1) activation, and improve obesity and associated disorders, such as insulin resistance and hepatic steatosis.

In summary, we have identified an anti-inflammatory small-molecule dye IR-61 which preferentially accumulates in the mitochondria of ATMs, suppresses macrophage activation, and prevents HFD-induced chronic inflammation, weight gain, and metabolic disorders. Mechanistic studies show that IR-61 enhances oxidative phosphorylation by activating ROS-Akt-Acly pathways, which mediate increasing expression and activity of the mitochondrial complexes in macrophages (Fig. 9). This multifunctional small-molecule agent holds great promise for further development of ATM-targeted therapeutic treatment and clinical applications.

## Methods

### Mice

C57BL/6J male mice were purchased from the Laboratory Animal Center of the Third Military Medical University. Mice were fed a normal chow diet (#1025, fat content 4% by calorie, HFKbio) or high-fat diet (D12492, fat content 60% by calorie, Research Diets) starting at 6 weeks of age for 10–24 weeks. IR-61 or BMS-303141(#SML0784, Sigma) was administered by intraperitoneal injection at 2 mg kg−1 body weight weekly. For the prevention groups, IR-61 was administered concurrently with the NCD or HFD feeding at 6 weeks of age. For the treatment groups, IR-61 was administered by i.p. injection starting at 18 weeks of age (after 12 weeks of HFD feeding). For the IR-61 + Acly inhibitor group, after receiving HFD for 8 weeks, mice were administered the treatment compounds for another 2 weeks. The environmental conditions in the mouse facility were: 12 h light and 12 h dark cycle, temperature range of 21–23 °C, humidity range of 40–50%, and free access to food and water. All animal use was approved by the Ethics Committee and performed in accordance with the Animal Care and Use Committee Guidelines of the Third Military Medical University.

### Indirect calorimetry

Oxygen consumption, carbon dioxide production, and heat production were obtained using the CLAMS (Columbus Instruments) open-circuit indirect calorimetry system. Animals were trained to acclimatize to the cages for at least 24 h before data acquisition and maintained in a 12-h light/dark cycle in individual chambers. Food and water were provided ad libitum. Flow rates of air is 0.5 L min^−1^ and cage size is 320 × 215 × 170 mm. Monitoring was for 24 h.

### Fat distribution and IR-61 biodistribution

Fat distribution in the NCD-fed and HFD-fed mice treated with IR-61 or a vehicle control for 10 weeks was detected by microCT. IR-61 was injected into mice (6 weeks of age, 2 mg kg^−1^) by intraperitoneal administration. After 24 h, mice were sacrificed, and epididymal adipose tissue, liver, spleen, heart, lung, and kidney were harvested for analysis ex vivo. NIR fluorescence images were captured using a Kodak In-Vivo FX Professional Imaging System equipped with fluorescence filter sets (excitation/emission 770/830 nm).

### IR-61 uptake by macrophages in mice

IR-61 was injected intraperitoneally into mice at 2 mg kg-1 body weight. After 24 h, mice were sacrificed and epi WAT, liver and spleen were harvested for analysis ex vivo. Adipose tissue was incubated with collagenase I for 30 min at 37 °C to isolate the SVFs. After centrifugation at 300*g* for 5 min, SVF cells were separated. Then SVFs, splenocytes and liver cells were stained with anti-mouse CD45 (#103131, Biolegend; dilution: 1:200), anti-mouse F4/80 (#123131, Biolegend; dilution: 1:200) and anti-mouse CD11C (#117310, Biolegend; dilution: 1:200), and then analyzed using flow cytometry. Flow cytometry analysis was performed using the LSRFortessaTM cell analyzer (BD) and subsequent analysis was performed using FlowJo V10.1.

### Mitochondrial function assay

Analysis of macrophage mitochondrial oxygen consumption rates (OCRs) was performed under different treatments in 8-well plates by using a XFp Cell Mito Stress Test Kit (#103010-100, Seahorse) on an XFp Flux Analyzer. In brief, native BMDMs were seeded into XFp cell culture microplates (#103022-100, Seahorse) and subjected to different treatments. Then, cells were transferred to a CO_2_-free incubator and maintained at 37 °C for 1 h after adding a final volume of 175 μl buffer-free assay medium to each well. Following instrument calibration, the cells were transferred to the XFp Analyzer to record cellular oxygen consumption rates. Basal cellular OCRs were recorded without any inhibitors and uncouplers. For the mitochondrial stress test, ATP synthase was inhibited by adding 1 μM oligomycin, followed by 2 μM FCCP-induced mitochondrial uncoupling to achieve maximal OCRs. Finally, nonmitochondrial respiration was determined after injection of a rotenone/antimycin A (1 μM each) combination. Once the XF experiment was completed, cell proteins were extracted to determine the protein concentration for normalization and the OCR was displayed as pmol min^−1^ μg^−1^ protein.

### Analysis of mitochondrial complexes/supercomplexes

Mitochondrial complexes and supercomplexes were analyzed according to published protocol^65^. A total of 50 μg isolated mitochondria were solubilized with digitonin, and the digitonin/protein ratio was 8 g/g. Solubilized mitochondria were incubated on ice for 20 min and then the supernatant was loaded into 10-well 3–12% BN gels (#BN1001BOX, Invitrogen) in a SureLock Xcell tank (Invitrogen), run, and transferred. Total OXPHOS Blue Native WB Antibody Cocktail (#ab110412, abcam; dilution: 1:250) was used to detect CI-CV simultaneously.

### Measurements of Complex I, II, and IV activity

Measurements of complex I activity (Complex I Enzyme Activity Microplate Assay Kit, #ab109721, Abcam), complex II activity (Complex II Enzyme Activity Microplate Assay Kit, #ab109908, Abcam), and complex IV activity (Complex IV Enzyme Activity Microplate Assay Kit, #ab109911, Abcam) in macrophages were taken according to the manufacturer’s instructions. BMDMs were treated with IR-61 for 24, 48, and 72 h, then cells were harvested and loaded onto the plate for 3 h. Then 200 μl assay solution was added and optical density was measured in kinetic mode for indicated time. Activity is expressed as the change in absorbance per minute (mOD min^−1^) per 200 μg of cell lysate.

### PMs and BMDMs preparation

Then, 6–10 weeks old C57BL/6J male mice were injected intraperitoneally with 3 ml of 3% thioglycollate. Three days following injection, the peritoneal cavity was washed with PBS and centrifuged. The pellet was plated in DMEM medium containing 10% fetal bovine serum (FBS) and 1% streptomycin/penicillin. After 2 h culturing, the floating cells were washed with PBS for 3 times. The remaining attached cells were considered as PMs to conduct the experiment. Bone marrow cells were isolated from femur and tibia of mice. BMDMs were generated in DMEM medium containing 10% FBS and recombinant murine M-CSF (#315-02, Peprotech) at 37 °C/5% CO_2_ for 7 days. Then the macrophages were collected for the experiments.

### ATMs’ isolation

ATMs (F4/80^+^ cells) were isolated from SVF cells using magnetic immunoaffinity isolation with anti-F4/80 antibodies conjugated to magnetic beads. Cells were isolated using positive selection columns (MACS) prior to preparation of whole-cell lysates for western blots.

### Microscopy

Fixed SVF cells from mice intraperitoneally administered IR-61 for 24 h were incubated with rat anti-mouse F4/80 antibody (#123101, Biolegend; dilution: 1:200) overnight at 4 °C followed by goat anti-rat Alexafluor 488 secondary antibody (#A-11006, Invitrogen; dilution: 1:500). Nuclei were stained using DAPI. Cells were imaged using a confocal fluorescence microscope (Leica TCS SP5). For IR-61 subcellular localization, PMs were incubated with 10 μM IR-61 for 30 min and then stained with Mito-tracker Green (#C1048, Beyotime) for another 30 min. The nuclei were stained by Hoechst33258 for 5 min then imaged by a confocal fluorescence microscope (Leica TCS SP5).

### Histology and adipocyte size

For histopathology, tissues were fixed in 10% neutral formalin and then embedded in paraffin. Tissues were sectioned at 5-μm thickness and placed on the slides, deparaffinized and stained with H&E. Tissue images were captured by a microscope (Olympus BX51). We invited a histopathologist to evaluate the H&E sections carefully without knowing the sample grouping. The adipocyte area was measured using image J software (version 1.52). Almost 200 adipocytes were measured from each group.

### Internalization assay

PMs were seeded in 6-well plates at 3 × 10^6^ cells per well. For inhibition experiments, macrophages were preincubated with 50 μM amiloride, 12.5 μM chlorpromazine, and 7.5 mM MβCD for 30 min and then incubated with 10 μM IR-61 for another 30 min. Cells were then washed and treated with 1 mM EDTA for 5 min, gently harvested, and transferred to microcentrifuge tubes on ice. After washing for 3 times and resuspension in flow cytometry buffer, at least 10,000 cells per sample were analyzed using a BD LSRFortessa cell analyzer (BD Biosciences). For fluorescence microscopy analysis, the nuclei of PMs were stained using Hoechst33258 after treating as described above. Fluorescent images were captured by an NIR fluorescent microscope (Leica, excitation/emission: 770/830). The mean fluorescent intensity was assessed using the Leica LAS AF Lite software. For flow cytometric analysis of IR-61 uptake in macrophages with different activation state, PMs were seeded in 6-well plates (3 × 10^6^ cells per well) and then added 100 ng ml^−1^ LPS (#L8880, Solarbio) or 20 ng ml^−1^ IL-4 (#96-214-14-5, Peprotech), respectively, for 24 h to induce M1 or M2 polarization. IR-61 was added and incubated with cells for another 2 h, then macrophages were harvested and analyzed using a BD LSRFortessaTM cell analyzer (BD Biosciences).

### Gene microarray

RAW264.7 cells were treated with vehicle control or IR-61 for 24 h and then RNA was extracted using Trizol reagent (#15596018, Invitrogen), RNA quantification and quality assurance were checked by NanoDrop ND-1000 (Thermo). Then the RNA was labeled and hybridized, placed on the slide. The slide was washed and scanned with Agilent Microarray Scanner (Agilent p/n G2565BA) and data were extracted using Agilent Feature Extraction Software (version 10.5.1.1). The expression of key genes involved in macrophage pro-inflammatory activation were displayed in a heat map. Original data of the sequencing were deposited in GEO under the accession codes GSE130718.

### Quantitative real-time reverse transcript PCR

After total RNA was extracted, cDNA synthesis was carried out with RevertAid First Strand cDNA Synthesis Kit (#K1622, Invitrogen) according to manufacturer’s instructions. Real-time PCR was performed using a SYBR green qPCR master mix (Takara). The relative expression of the selected genes was measured using CFX Connect Real-Time System (Biorad). The premiers for the qRT-PCR are listed in Supplementary Table 1.

### Enzyme-linked immunosorbent assay

The concentrations of mice inflammatory cytokines, TNF, IL-6, and IL-1β, from the cultured macrophage supernatant or mice plasma samples were directly quantified by TNF (#CSB-E04741m), IL-6 (#CSB-E04639m), IL-1β (#CSB-E08054m) ELISA kits from CUSABIO. BMDMs were treated with 10 μM IR-61 or vehicle control for 24 h prior to treatment with 100 ng ml^−1^ LPS for 24 h, and then collected the medium and subjected to ELISA according to the manufacturer’s instructions. For measurement of mice plasma samples, whole blood in mice were stood for 2 h at room temperature before centrifuge at 1500 rpm for 20 min and then the supernatants were subjected to ELISA according to the manufacturer’s instructions.

### Western blot analysis

Tissues and cell proteins were extracted with a cell lysis buffer for western and IP (Beyotime) added with 1× Cocktails 1, 2, and 3 containing protease and phosphatase inhibitors (Sigma). The concentrations were determined using a BCA kit (Beyotime). The extracted proteins were separated by 10% or 12% SDS-polyacrylamide gel electrophoresis (SDS-PAGE), and then transfered to a polyvinylidene difluoride membrane. The membrane was blocked with blocking buffer and incubated with a primary antibody [Anti-AKT (#4685, Cell Signaling; dilution: 1:1000), Anti-Phospho-AKT (Thr308) (#2965, Cell Signaling; dilution: 1:1000), Anti-NF-ĸb p65 (#8242, Cell Signaling; dilution: 1:1000), Anti-Phospho-p65 (#3033, Cell Signaling; dilution: 1:1000), Anti-SAPK/JNK (#9252, Cell Signaling; dilution: 1:1000), Anti-Phospho-SAPK/JNK (Thr183/Tyr185) (#4668, Cell Signaling; dilution: 1:1000), Anti-total OXPHOS cocktail (#ab110413, Abcam; dilution: 1:200), Anti-ATP citrate lyase (#ab40793, Abcam; dilution: 1:1000), Anti-Phospho-ATP citrate lyase (Ser455) (#4331, Cell Signaling; dilution: 1:1000), Anti-AMPK (#5831, Cell Signaling; dilution: 1:1000), Anti-Phospho-AMPK (#2535, Cell Signaling; dilution: 1:1000), Anti-ACC (#3676, Cell Signaling; dilution: 1:1000), Anti-Phospho-ACC (#11818, Cell Signaling; dilution: 1:1000), Anti-UCP1 (#23673-1-AP, Proteintech; dilution: 1:1000), Anti-β-Actin (#AF0003, Beyotime; dilution: 1:2000), Anti-GADPH (#1E6D9, Proteintech; dilution: 1:1000)] at 4 °C overnight. Horseradish peroxidase-conjugated secondary antibodies (Beyotime) were applied for 1 h at room temperature. The membrane was incubated with Enhanced Chemiluminescence Substrate for 1 min and the signals were captured using an enhanced chemiluminescence detection system (LabImage version 3.0 (BioRad)).

### GTT and ITT

For GTT, after a 12-h fast, basal blood glucose levels of mice in each group were measured by cutting tail veins. Then the mice were administered glucose solution at 1.5 g kg^−1^ body weight by intraperitoneal injection, and blood glucose levels were measured at 15, 30, 60, and 120 min after glucose injection. For ITT, after a 4-h fast, we measured the basal blood glucose of each group of mice. Then the mice were given an intraperitoneal injection of insulin (#P3376, Beyotime) (1.0 and 1.5 IU kg^−1^ body weight for NCD-fed and HFD-fed mice, respectively). The blood glucose levels were measured at 15, 30, 60, and 120 min after insulin administration.

### Acute tissue insulin signaling tests

For examination of insulin signaling in vivo, overnight-fasted mice on HFD for 15 weeks were anesthetized and injected with human insulin (0.5 U kg^−1^ body weight) or vehicle saline. Liver, soleus muscle, and epididymal fat were collected 5 min later, flash-frozen in liquid nitrogen and stored at −80 °C until further experiments to determine phosphorylation of Akt (Thr308), total Akt and β-actin by western blot.

### Measurements of lipids in liver and plasma

Almost 100 mg liver tissues were placed in 1.5 ml EP tubes containing 900 μl 100% ethanol, then pulverized mechanically in ice-water bath. The tube was centrifuged at 2500 rpm for 10 min and the supernatant was collected to lipid measurements. Manufacturer’s protocols were followed to measure the levels of triglycerides (Triglyceride assay kit, #A110-2, Nanjing Jiancheng) and total cholesterol (Total cholesterol assay kit, #A111-2, Nanjing Jiancheng) in lipid extracts and plasma samples.

### Fecal lipid analysis

Feces was collected daily, dried, and weighted. Then the dry fecal sample was added into the mixture of deionized water and concentrated hydrochloric acid, put in 80 °C water bath for 50 min and then mixed with anhydrous ethanol. This mixture was transferred into separating funnel after cooling, washed the fat attached to the opening of the separating funnel with ether several times, pluged and shaked for 30 s, made to stand for 15 min, and then sucked out the supernatant and put it into a flask at the constant weight. The flask was dried in an oven at 105 °C for 1 h and cooled in a desiccator, weighed after 20 min, and repeatedly dried and cooled to constant weight.

### Cytosolic and mitochondrial ROS

BMDMs were seeded in 6-well plates and treated with the fluorescent dyes DCFH-DA (#S0033, Beyotime) and MitoSOX Red (#M36008, Invitrogen) to measure the intracellular ROS and mitochondrial superoxide levels, respectively. Mito-Tracker Green was used to detect mitochondrial content. BMDMs were incubated with Mito-Tracker Green (#C1048, Beyotime) at 37 °C for 30 min and then analyzed by flow cytometry, using the Accuri C6 (BD) and subsequent analysis was performed using CFlow Plus.

### Mitochondrial content

BMDMs were incubated with Mito-Tracker Green (#C1048, Beyotime) at 37 °C for 30 min and then analyzed using flow cytometry. Mitochondrial DNA copy number was determined by real-time PCR using specific probes against CytB and Ppia.

### Mitochondrial membrane potential

BMDMs were treated with IR-61 (10 μM) or vehicle control for 24 h, then incubated with 100 nM Tetramethylrhodamine, methyl ester (TMRM) (#T668, Invitrogen) at 37 °C for 30 min, and analyzed using flow cytometry. Mitochondrial membrane potential was estimated by measuring the fluorescence intensity of TMRM.

### Citrate synthase activity

Citrate synthase activity was determined using a Citrate Synthase Activity Assay Kit (#ab239712, Abcam). BMDMs were treated with IR-61 (10 μM) or vehicle control for 24 h and then incubated with ice cold CS assay buffer for 10 min. After centrifugation at 10,000*g* for 5 min, the supernatant was collected and mixed with reaction buffer and then absorbance (OD412 nm) was measured immediately in kinetic mode at 25 °C for 40 min. Protein concentration was determined for normalization and the citrate synthase activity was displayed as nmol min^−1^ mg^−1^ protein.

### Cardiolipin assay

Cardiolipin of isolated mitochondria was determined using a Cardiolipin Assay Kit (#ab241036, Abcam). Control and Acly knockdown BMDMs treated with IR-61 in the absence or with BMS-303141, then mitochondria were isolated and suspended in CL assay buffer. After centrifugation at 10,000*g* for 5 min, the supernatant was collected and mixed with CL probe. The fluorescence at Ex/Em 340/480 nm was recorded after incubating at room temperature for 10 min. Protein concentration was determined for normalization and the mitochondrial cardiolipin content was displayed as nmol mg^−1^ protein.

### Apoptosis analysis

BMDMs were incubated with various concentrations of IR-61 for 72 h. Then apoptosis was detected by flow cytometry using an Annexin V/PI staining kit (BD Biosciences), according to the manufacturer’s instructions. The cells were harvested and washed, resuspended in 1× annexin-binding buffer, and incubated with annexin-V and PI for 15 min at room temperature in the dark. Cells were then analyzed using flow cytometry.

### Measurements of cellular ATP levels and ADP/ATP ratio

Cellular ATP levels were determined in macrophages by an ATP Assay Kit (#S0027, Beyotime) following manufacturer’s protocol. The value in IR-61-treated macrophages was normalized to that in vehicle control, which was defined as 100%. ADP/ATP ratio in macrophages was measured by an ADP/ATP Ratio Assay Kit (#ab65313, abcam). BMDMs were treated with IR-61 (10 μM) or vehicle control for 24 h, washed and harvested after mixing with nucleotide releasing buffer and incubated for 5 min at room temperature with gentle shaking. Then, 100 μl prepared reaction mix was added in control wells and the background luminescence was read (Data A), then 50 μl sample was added and after 2 min the luminescence was read (Data B). To measure ADP levels in the cells, the samples were read again (Data C), then added 10 µL of 1× ADP converting enzyme and read the samples again after approximately 2 min (Data D). ADP/ATP ratio = [Data D − Data C] / [Data B − Data A].

### siRNA transfection

siRNA targeted to mouse Acly and scramble-siRNA were purchased from GenePharma Biotechnology. BMDMs were transfected with siRNA using Lipofectamine 3000 (#L3000008, Invitrogen) in OptiMEM (#31985062, Gibco) according to the manufacturer’s protocol. After transfection, fresh medium was replaced for culture at 6 h, and other experiments were conducted 24 h later.

### Statistics

Statistical analyses were performed using statistical tools in GraphPad Prism 8.4.3. Data are expressed as the mean ± SEM. Statistical analyses were applied using two-sided Student’s *t*-test and two-way analysis of variance (ANOVA) with post hoc analysis to determine statistical significance (*p* < 0.05). When comparing VO_2_, VCO_2_, and heat production in IR-61-treated and vehicle-treated obese mice, adjustment was made for the covariate body weight using multiple linear regression analysis (ANCOVA), which were carried out in SPSS 26.0.0.

### Reporting summary

Further information on research design is available in the Nature Research Reporting Summary linked to this article.

## Supplementary information

Supplementary Information

Peer Review File

Reporting Summary

## Data Availability

The authors declare that data supporting the findings of this study are available within the article and its Supplementary Information files, and from the authors upon reasonable request. Microarray data have been deposited in GEO under the accession code GSE130718. Source data are provided with this paper.

## Source data

Source Data
