# Supplementary material for: Improvement of obesity-associated disorders by a small-molecule drug targeting mitochondria of adipose tissue macrophages
Source: Nat Commun. 2021 Jan 4;12:102. doi: 10.1038/s41467-020-20315-9 (PMC7782823; doi:10.1038/s41467-020-20315-9)
Supplement: Supplementary file 1 — Supplementary Information [file 41467_2020_20315_MOESM1_ESM.pdf]

Supplementary Information

Improvement of obesity-associated disorders by a small-molecule drug targeting mitochondria of adipose tissue macrophages

Wang *et al.*

Supplementary note: Chemistry

Synthetic procedures

1. General Methods and Materials

All used reagents and solvents were purchased reagent grade or higher from commercial sources (Aladdin, Sigma-Aldrich, Thermo Fisher Scientific Inc., Merck KGaA, Acros Chemicals) and were used without further purification. Technical grade solvents were always distilled prior to use. Ultrapure water was produced using a Milli-Q Integral 5 system and used in all experiments. All reactions were conducted using oven dried glassware whereas reactions containing oxygen or water sensitive reagents were carried under argon atmosphere.

For reaction control, analytical thin-layer chromatography (TLC) was carried out on Merck silica-gel 60 F254 plates, using short wave UV light (λ=254 nm and 366 nm). Flash chromatography was carried out using silica gel (300-400 mesh).

^{1}H NMR, ^{13}C NMR, spectra were recorded on a Bruker 400 MHz spectrometer at room temperature with $CDCl_3$ or DMSO-*d*6 as solvents and referenced to the residual proton signal of the corresponding deuterated solvent ($CDCl_3$: δ = 7.26 ppm, DMSO-*d*6: δ = 2.50 ppm). Chemical shifts are reported in parts per million (ppm). Tetramethylsilane (TMS) was used as the internal standard. High Resolution Mass Spectrometry (HRMS) was performed in a Bruker BioTOF IIIQ.

High resolution mass spectra were recorded using an *LTQ-FT Ultra* (*Thermo Fisher Scientific Inc.*) coupled with a Dionex UltiMate 3000 HPLC system and an ESI or APCI ion source.

2. Synthesis

In this work, six NIR fluorescent heptamethine cyanine dyes (IR-43, IR-28, IR-77, IR-86, IR-808 and IR-61) were synthesized as shown in the following **Supplementary Figure 1**, and their side chains contained carboxy or hexagonal ring structures, respectively.

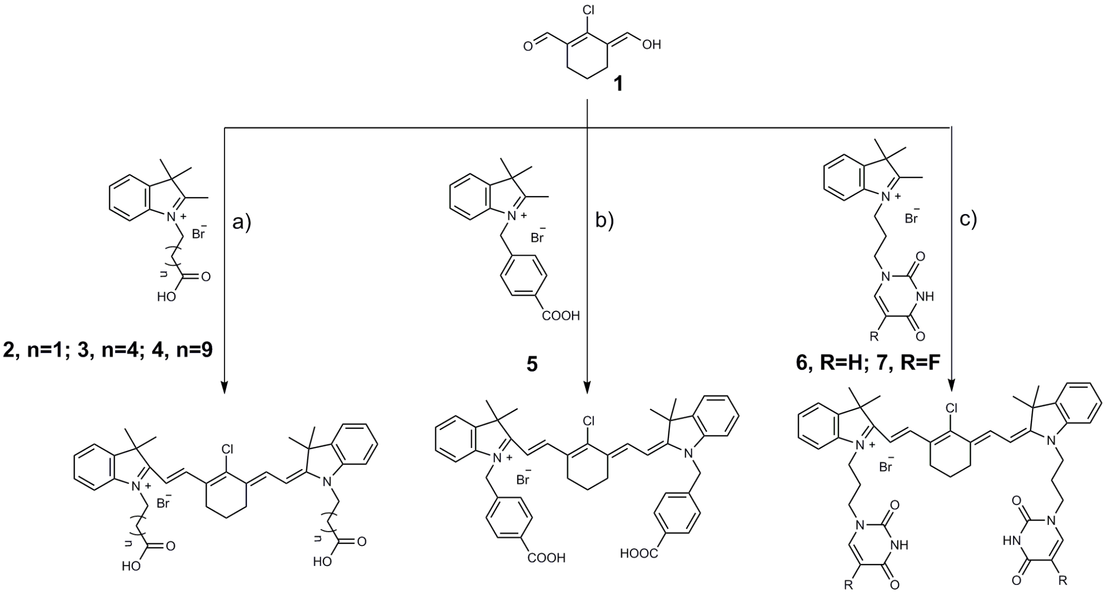

n=1, IR-43; n=4, IR-808; n=9, IR-28 **IR-61** **R=H, IR-77; R=F, IR-86**

Supplementary Figure 1. The synthetic routes for preparing heptamethine cyanine dyes (IR-43, IR-28, IR-77, IR-86, IR-808 and IR-61). a) NaOAc, EtOH, 80 °C, 1-5 h; b) n-butyl alcohol/toluene (7/3, v/v), reflux, 4 h.

2-chloro-3-(hydroxymethylene)cyclohex-1-ene-1-carbaldehyde(1). **1** was synthesized according to our previously reported protocol. 40 mL DMF and 40 mL dichloromethane were added to the 250 mL reaction flask, the internal temperature was cooled to -5 °C in ice salt bath, 37 mL phosphorus oxychloride was slowly dripped with constant pressure drop funnel, and the internal temperature was controlled not more than 5 °C. Then 10 g cyclohexanone was dripped to make the internal temperature lower than 10 °C. After dripping, the temperature was heated to 80 °C and stirring reaction. After 3.5 h, stop the reaction and cool to room temperature, reduce pressure to remove dichloromethane, got brownish yellow oily viscous liquid. Then, slowly add this liquid to 200 g ice, and stir overnight at room temperature. Brinell funnel filter, filter cake with ice water washing 3 times to get yellow solid. The obtained solid was recrystallized with acetone, and the yellow crystal was 10.4 g with a yield of 59.8%. Melting point: 128-129 °C.

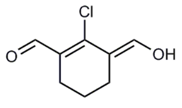

1

1-(n-carboxyethyl)-2,3,3-trimethyl-3H-indol-1-ium bromide(2-4). 2,2,3-trimethyl -3H-indolenine (30 mmol) and bromosubstituted compounds with short alkyl chains (30 mmol) were mixed in 1,2-dichlorobenzene (15 ml) and stirred at 110 °C under argon protection for 12 h. The reaction mixture was cooled to room temperature and added to acetone. The brown precipitate or oil was collected and washed with acetone. Indolenine quaternary ammonium salts **2-4** were afforded and straightforward used in this next reaction without further purification.

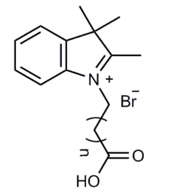

2, n=1; 3, n=4; 4, n=9

1-(4-carboxybenzyl)-2,3,3-trimethyl-3H-indol-1-ium bromide(5). 2,3,3-trimethyl-3H-indole (4.90 g, 0.031 mol) and 4-(bromomethyl)benzoic acid (6.70 g, 0.031 mol) was added to three-mouth flask(50 mL), and then o-dichlorobenzene(15 mL) was added to reaction in 110 °C for about 8 h. After that, the reaction liquid was cooled to room temperature, add isopropyl ether (30 mL), stir and mix well, and filter with reduced pressure. The filter cake was washed with 30 mL isopropyl ether, filtered under reduced pressure, and dried by vacuum at 40 °C to obtain purple red solid with yield of 109.1%. The product is directly used for the next reaction without purification.

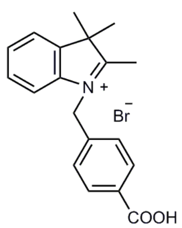

5

1-(3-(5-hydro/fluoro-2,4-dioxo-3,4-dihydropyrimidin-1(2H)-yl)propyl)-2,3,3-trimethyl-3H-indol-1-ium bromide(6, 7). Uracil or 5-Fluorouracil (12.0 mmol) was heated to dissolve in 1,1,1,3,3,3 -hexamethyldisilazane (48.0 mmol) under argon. Then chlorotrimethylsilane (250 uL) was added and the solution was stirred for 4.5 h at 126 °C. The reaction mixture was cooled and the excess solvent was removed under reduced pressure to yield yellow green oil. This oil was immediately used to react with dried 1, 3-dibromoalkane (10 mL) under 105 °C for 3 h. The reaction mixture was cooled to the room temperature, and the dichloromethane was extracted as the organic phase from water, and the organic phase was dried with anhydrous sodium sulfate, filtered, and concentrated under reduced pressure to obtain the crude product. The crude product was then recrystallized from the ethyl acetate/petroleum ether system to give a pale yellow solid. A reaction mixture containing 2,3,3-trimethyl-3H indole (3.67 mmol), the above brominated substances (1.83 mmol) was reacted in 1,2-dichlorobenzene at 110 °C for 10 h. After the reaction, the reaction mixture was washed with acetone and recrystallized in isopropyl alcohol to obtain the quaternary ammonium salt of indolium. No further purification was required.

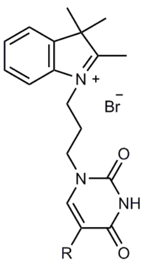

6, R=H; 7, R=F

1-(2/5/10-carboxyethyl)-2-((E)-2-((E)-3-(2-((E)-1-(2-carboxyethyl)-3,3-dimethylindolin-2-ylidene)ethylidene)-2-chlorocyclohex-1-en-1-yl)vinyl)-3,3-dimethyl-3H-indol-1-ium bromide(IR-34, IR-808 and IR-28). A mixture of quaternary ammonium salt 2-4 (7.1 mmol), 1 (3.3 mmol), anhydrous sodium acetate (7.1 mmol) was mixed in anhydrous ethanol (15 ml) was shaken at 80 °C for 1-4 h. After completion of the reaction, the ethanol was evaporated under reduced pressure and the green residue was purified by flash chromatography to obtain pure green product.

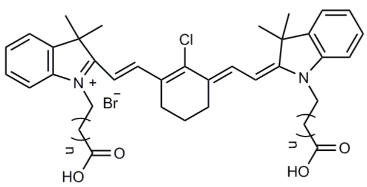

n=1, IR-43; n=4, IR-808; n=9, IR-28

IR-43: ^{1}H NMR(400Hz, DMSO-d6) δ: 12.60(s, 2H), 8.25(d, J=14.0Hz, 2H), 7.63(d,J=6.4Hz,2H), 7.46-7.40(m,4H), 7.30-7.26(m,2H), 6.43(d, J=14.0Hz,2H), 4.44(t, J=6.4Hz,4H), 2.81-2.72 (m,8H), 1.85(s,2H), 1.66(s,12H); HRMS[M-Br]$^+$: calc. 599.2671, measured 599.2587.

IR-808: ^{1}H NMR (400 MHz, $CDCl_3$) δ: 1.557(m, 4H), 1.706(s, 12H), 1.766(m, 4H,), 1.856(m, 4H), 2.008(s, 2H), 2.484 (s, 4H), 2.738(s, 4H), 4.120(t, 4H), 6.216(d, 2H), 7.169-7.413 (m, 8H), 8.325(d, 2H); ^{13}C NMR(100 MHz, DMSO-d6) δ: 174.223, 172.180, 171.925, 147.948, 147.930, 142.004, 141.021, 128.598, 126.156, 125.134, 122.478, 111.489, 101.573, 48.945, 43.629, 33.402, 27.434, 26.680, 25.788, 25.597, 24.123, 20.971, 20.339. HRMS[M-Br]$^+$: calc. 683.3616, measured 683.3610.

IR-28: ^{1}HNMR(400Hz, DMSO-d6): 7.64(d, J=7.2Hz, 2H), 7.47-7.41(m, 4H), 7.29(t, J=7.6Hz, 2H), 1.67(s,12H), 6.33(d, J=14.4Hz, 2H), 8.26(d, J=14.4Hz, 2H); 2.71(s, 4H); 1.86(s, 2H); 4.22(t, J=6.4Hz, 4H); 1.73-1.71(m, 4H); 1.36-1.24(m, 20H); 1.46(t, J=6.4Hz,4H); 2.07(t, J=7.6Hz, 4H); 11.9(s, 2H); HRMS[M-Br]+: calc. 795.4698, measured 795.4768

1-(4-carboxybenzyl)-2-((E)-2-((E)-3-(2-((E)-1-(4-carboxybenzyl)-3,3-dimethylindolin-2-ylidene)ethylidene)-2-chlorocyclohex-1-en-1-yl)vinyl)-3,3-dimethyl-3H-indol-1-ium bromide(IR-61). Bisaldehyde **1** (0.57 g, 3.31 mmol), Compound **5** (2.55 g, 7.1 mmol), anhydrous sodium acetate (0.58 g, 7.1 mmol) and anhydrous ethanol were added to the 25 mL reaction flask. The reaction was heated for 1.5 h under 80 °C. After that, the reaction solution was cooled to room temperature, and the solvent was removed by vacuum concentration, and the residue was dissolved in 30 mL dichloromethane. Add deionized water (15 mL) was added to oscillate and mix, static, separate liquid to take dichloromethane phase, vacuum concentration and steam drying. The residue was dissolved with a small amount of dichloromethane, and isopropyl ether was slowly dripped into it at room temperature. A large number of solids were precipitated and attached to the bottle wall at room temperature. There were almost no products in the liquid detected by TLC, and the mixed solvent of dichloromethane and isopropyl ether was removed. Repeat this operation to get dark green products. Purity 99.3%; Melting point: 202-208 °C.

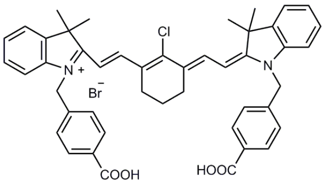

IR-61

IR-61: ^{1}H NMR (400 Hz, DMSO)-d6) δ: 8.26 (d, J = 14.4 Hz, 2H), 7.94 (d, J = 8.0 Hz, 4H), 7.69 (d, J = 7.6 Hz, 2H), 7.40 (t, J = 7.2 Hz, 8H), 7.33–7.28 (m, 2H), 6.38 (d, J = 14.0 Hz, 2H), 5.63 (s, 4H), 2.53–2.50 (m, 4H), 1.73 (s, 14H); ^{13}C NMR (100 MHz, DMSO-d6) δ: 172.901, 167.239, 148.461, 143.525, 142.331, 140.939, 129.925, 129.664, 129.502, 128.749, 127.023, 126.575, 125.393, 111.638, 102.334, 99.592,49.160, 48.591, 46.979, 45.218, 27.858, 27.604, 25.700, 19.904; HRMS [M-Br]+: calc. 723.2984, measured 723.2882.

2-((E)-2-((E)-2-chloro-3-(2-((E)-1-(3-(5-fluoro-2,4-dioxo-3,4-dihydropyrimidin-1(2H)-yl)propyl)-3,3-dimethylindolin-2-ylidene)ethylidene)cyclohex-1-en-1-yl)vinyl)-1-(3-(5-hydro/fluoro-2,4-dioxo-3,4-dihydropyrimidin-1(2H)-yl)propyl)-3,3-dimethyl-3H-indol-1-ium bromide(IR-77, IR-86). A reaction mixture containing 6 or 7 (0.71 mmol) and bisaldehyde **1** (0.31 mmol) in a refluxed solution of toluene and n-butyl alcohol (10 mL, 7:3) was allowed to react for 12 h where upon the color of the solution changed from red to green. After the reaction, the solvent was removed under reduced pressure and the residue was purified by silica gel column chromatography to achieve the target compounds.

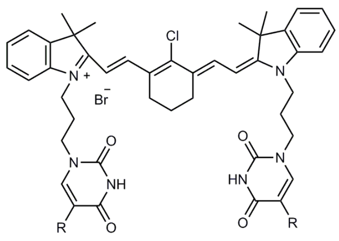

R=H, IR-77; R=F, IR-86

IR-77: ^{1}H NMR (400 MHz, DMSO-d) δ 11.27(s, 2H), 8.24-8.22(d, J=12Hz, 2H), 7.69-7.68(d, J=6Hz, 2H), 7.62-7.61(d, J=6Hz, 2H), 7.46-7.40(m, 4H), 7.28-7.26(t, J=6Hz, 2H), 6.23-6.21(d, J=12Hz, 2H), 5.57-5.56(s, J-6Hz, 2H), 4.23(m, 4H), 3.82-3.80(t, J=6Hz, 4H), 2.64(m, 4H), 2.03(m, 2H), 1.84-1.82(m, 2H), 1.65(s, 12H). ^{13}C NMR(100 MHz, DMSO-d6): δ 172.611, 164.144, 151.451, 148.630, 145.902, 143.509, 142.374, 141.508, 129.055, 126.807, 125.684, 123.023, 111.820, 101.971, 101.636, 49.495, 45.476, 41.673, 27.861, 27.076, 26.308, and 20.747; HRMS[M-Br]$^{+}$: calc. 759.3254, measured 759.3268.

IR-86: ^{1}H NMR (400 MHz, DMSO-d): δ 8.70-8.66(d, J=16Hz, 2H), 8.58-8.56(d, J=8Hz, 2H), 8.07-8.05(d, J=8Hz,2H), 7.92-7.84(m, 4H), 7.74-7.70(t, J=8Hz, 2H), 6.69-6.66(d, J=12Hz, 2H), 4.70-4.66(m, 4H), 4.24-4.21(t, J=6Hz, 4H), 3.82(s, 2H), 3.09(s, 6H), 2.10(s, 12H). ^{13}C NMR(100 MHz, DMSO-d6): δ 174.223, 172.180, 171.925, 147.948, 147.930, 142.004, 141.021, 128.598, 126.156, 125.134, 122.478, 111.489, 101.573, 48.945, 43.629, 33.402, 27.434, 26.680, 25.788, 25.597, 24.123, 20.971, and 20.339. HRMS$[M-Br]^+$: calc. 795.3217, measured 795.3228.

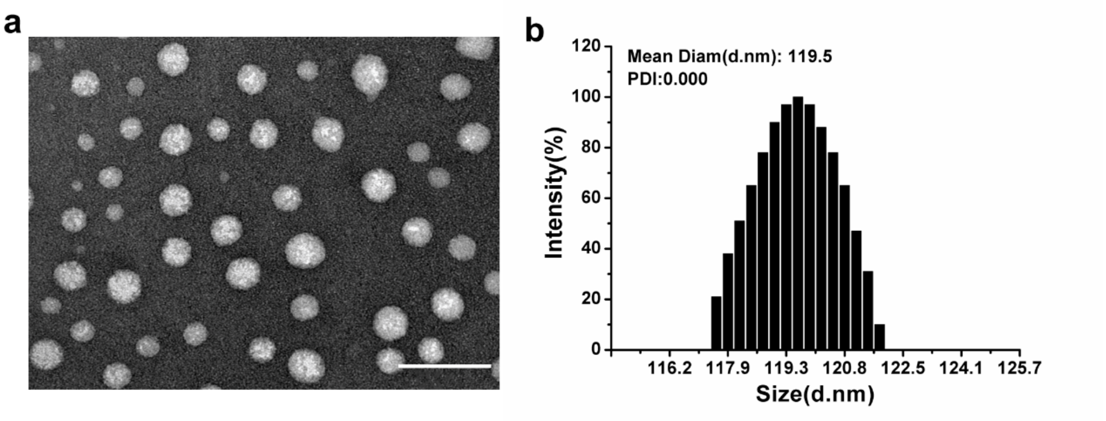

Supplementary Figure 2. (a) TEM images of IR-61 in aqueous solution, scale bar: 200 nm. **(b)** Hydrodynamic diameters of IR-61 measured by DLS.

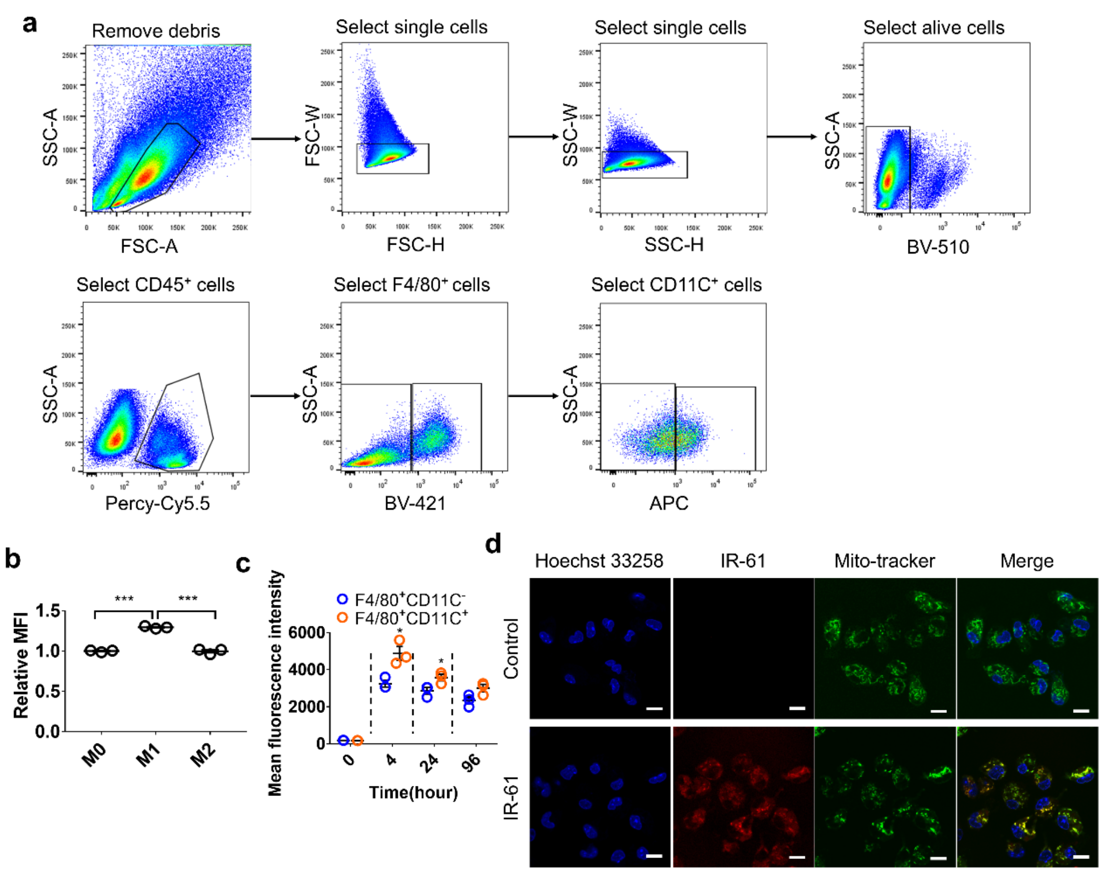

Supplementary Figure 3. IR-61 preferentially accumulates in M1-like macrophages. (a) Flow cytometry gating scheme for determining IR-61 content of the cells from adipose tissue, liver and spleen in mice. **(b)** In vitro cell uptake of IR-61 by

M1-like, M2-like and unstimulated primary PMs was measured as mean fluorescence intensity via flow cytometry. **(c)** Mean fluorescence intensity of M1-like ATMs (F4/80$^+$CD11C$^+$) and non-M1-like ATMs (F4/80$^+$CD11C$^-$). **(d)** ATMs were isolated from SVF cells at 0.5 h after mice being intraperitoneally injected with IR-61 (2 mg/kg). Localization of IR-61 in ATMs imaged with a confocal microscope after being stained with MitoTracker Green and Hoechst 33258. Representative images are displayed. (Scale bars, 10 μm). Data are representative result of three independent experiments. Results are presented as the mean ± SEM (**p*<0.05, ***p*<0.01, ****p*<0.001, *n*=3; two-sided Student's *t*-test). Exact *p*-values are **(b)** M0 vs M1 *p*<0.0001, M2 vs M1 *p*=0.0001, **(c)** 4 h *p*=0.0170, 24 h *p*=0.0446.

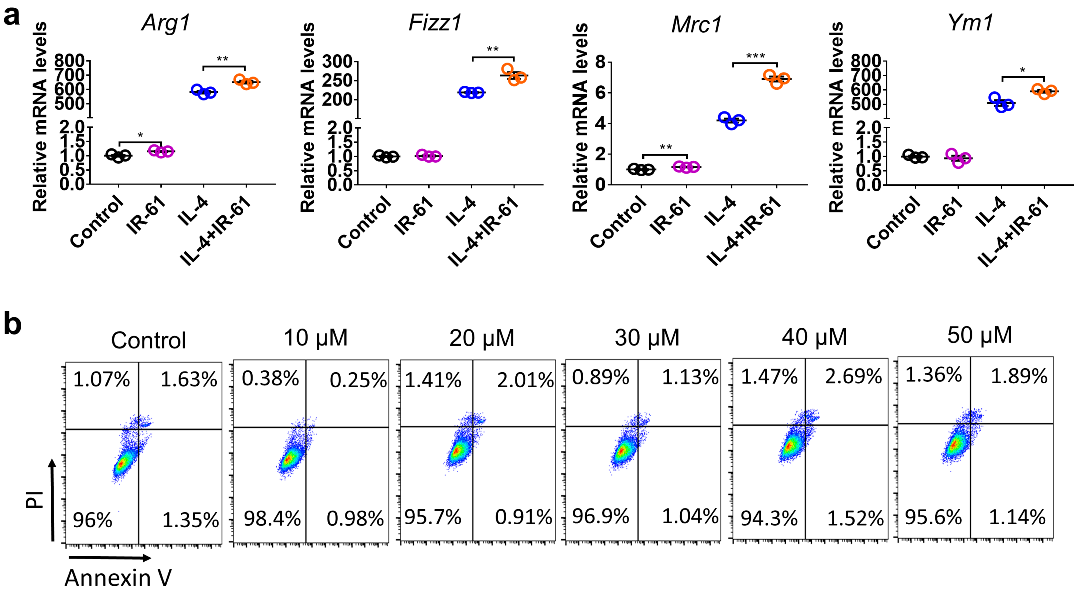

Supplementary Figure 4. IR-61 promotes macrophage M2 activation and has no cytotoxicity effect. (a) BMDMs were treated with or without additional 10 μM IR-61 for 24 h and then treated with or without 10 ng/ml IL-4 for another 24 h. Relative expression levels of the signature genes associated with M2 activation were determined by qPCR. **(b)** Cytotoxicity effect of IR-61 on BMDMs after 72 h treatment. BMDMs were incubated with various concentrations of IR-61 (0, 10 μM, 20 μM, 30 μM, 40 μM and 50 μM) for 72 h and then stained with Annexin-V/PI to detect the mode of death. Data are representative result of three independent experiments. Results are presented as the mean ± SEM (**p*<0.05, ***p*<0.01, ****p*<0.001, *n*=3; two-sided Student's *t*-test). Exact *p*-values are **(a)** *Arg1* *p*=0.0157, *p*=0.0065, *Fizz1* *p*=0.5528, *p*=0.0078, *Mrc1* *p*=0.0066, *p*=0.0002, *Ym1* *p*=0.5619, *p*=0.0213.

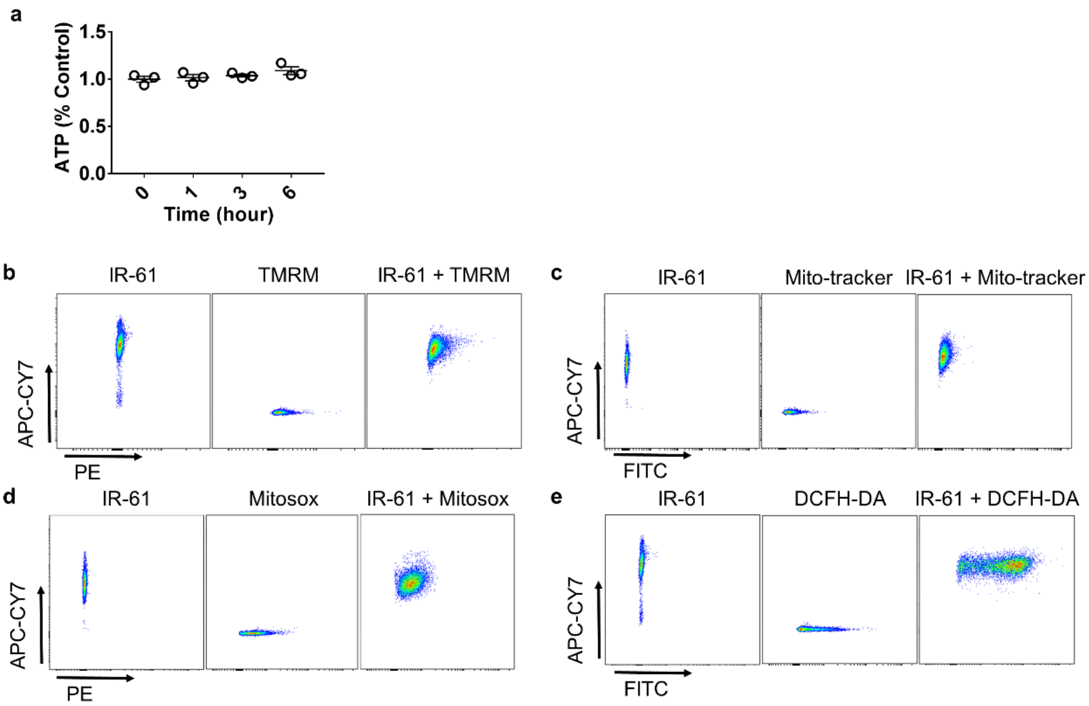

Supplementary Figure 5. IR-61 has no interference with ATP assay or fluorescent dyes. (a) BMDMs were treated with 10 μM IR-61 or vehicle for 1 h, 3h and 6h, then ATP levels were measured (n=3). **(b-e)** BMDMs were co-stained with IR-61 and TMRM, Mito-tracker Green, Mitosox or DCFH-DA, respectively, and then analyzed with a FACS system. Data are representative result of three independent experiments. Results are presented as the mean ± SEM (two-sided Student's t-test). Exact p-values **(a)** 0 vs 1, p=0.7311 0 vs 3, p= 0.3340, 0 vs 6 p=0.1553.

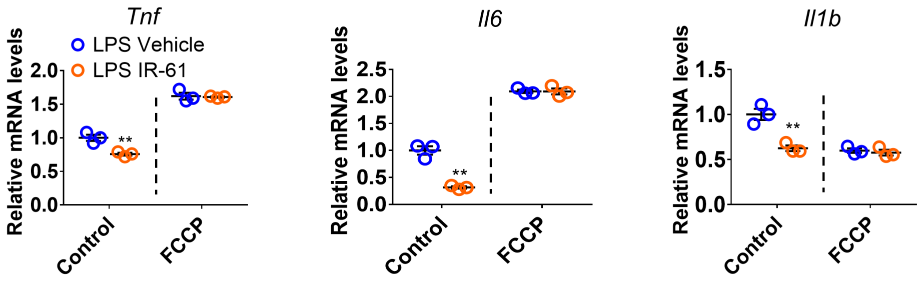

Supplementary Figure 6. Mitochondria oxidative function involves anti-inflammatory function of IR-61. Data are representative result of three independent experiments. Results are presented as the mean ± SEM (*p<0.05, **p<0.01, n=3; two-sided Student's t-test). Exact p-values are *Tnf* p=0.0085, *Il6* p=0.0011, *Il1b* p=0.0057.

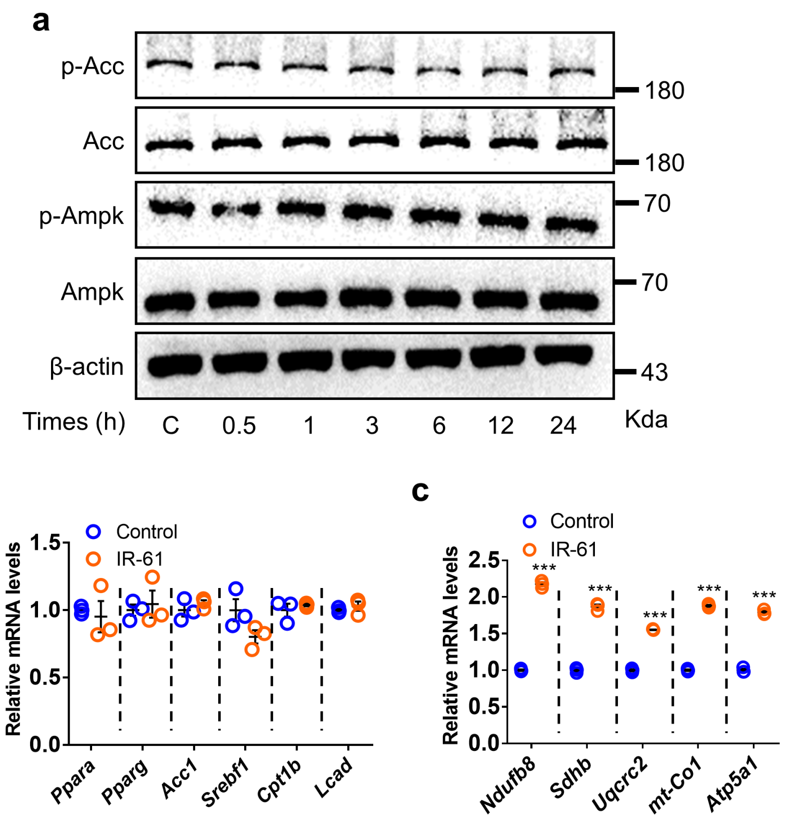

Supplementary Figure 7. The effect of IR-61 on metabolism-related genes and AMPK activity. (a) Immunoblots of Ampk, p-Ampk, Acc and p-Acc in whole cell lysates from the IR-61-treated BMDMs for the indicated times. β-actin was used as the loading control. **(b)** Relative mRNA levels of the genes associated with fatty acid oxidation in the BMDMs. **(c)** Relative mRNA levels of mitochondrial respiratory chain subunits in the BMDMs treated by IR-61 or vehicle control for 24 h. Data are representative result of three independent experiments. Results are presented as the mean ± SEM (***p<0.001, n=3; two-sided Student's t-test). Exact p-values are **(b)** *Ppara* p=0.7047, *Pparg* *p=0.7008, Acc1* p=0.3685, *Srebf1* p=0.1079, *Cpt1b* p=0.4729, *Lcad* p=0.4495, **(c)** *Ndufb8* p<0.0001, *Sdhb* p<0.0001, *Uqcrc2* p<0.0001, *mt-Co1* p<0.0001, *Atp5a1* p<0.0001.

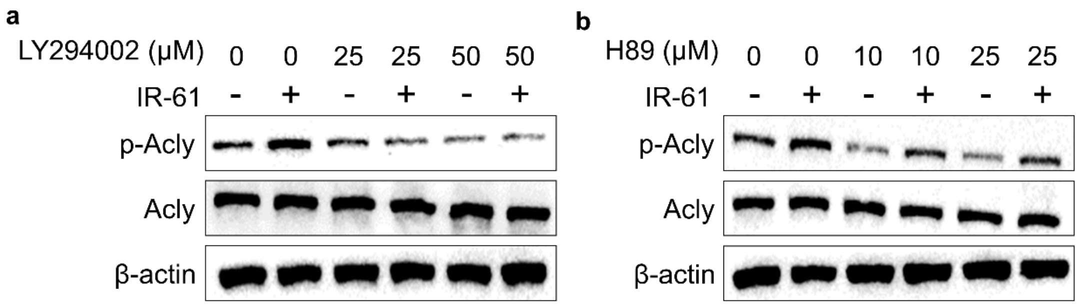

Supplementary Figure 8. IR-61 promoted phosphorylation of Acly at S455 through Akt. (a, b) Western blot analysis of Acly and p-Acly levels from BMDMs

being treated with LY294002 or H89 in the presence or absence IR-61 for 6 h. Panels are representative result of three independent experiments.

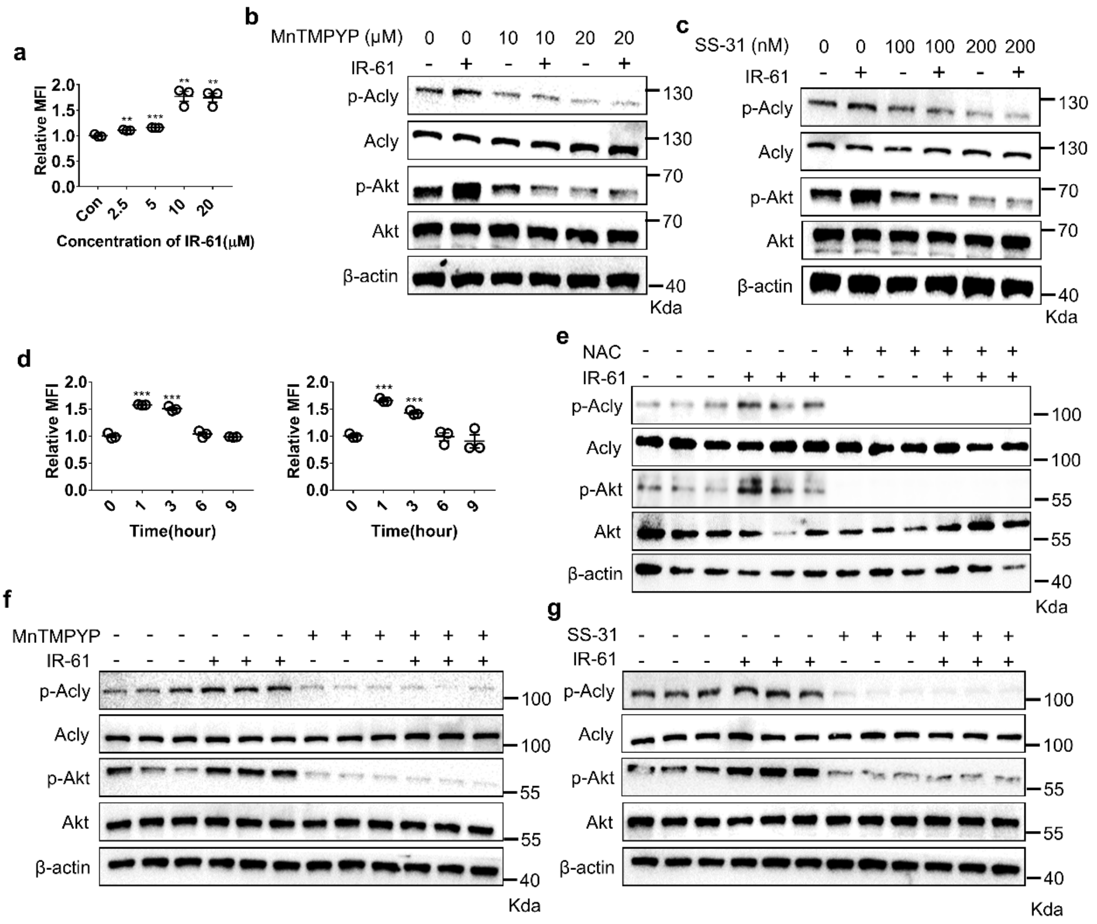

Supplementary Figure 9. IR-61 promotes Akt/Acly phosphorylation depending on mtROS. (a) Mitochondrial superoxide variation at 0.5 h after various concentrations of IR-61 (0, 2.5 μM, 5 μM,10 μM and 20 μM) treatment. **(b, c)** Western blot analysis of Acly, p-Acly, Akt, and p-Akt levels from BMDMs being treated with MnTMPYP or SS-31 in the presence or absence IR-61 for 6 h. **(d)** Mitochondrial superoxide and intracellular ROS variation at 0-9 h in ATMs after IR-61 injection. **(e-g)** Mice were intraperitoneally injected with IR-61 or vehicle control at 2 h after intraperitoneally administered NAC, MnTMPYP or SS-31. ATMs were isolated from SVF cells 6 h later. Immunoblots of Acly, p-Acly, Akt and p-Akt in ATMs. β-actin was used as the loading control. Data are representative result of three independent experiments. Results are presented as the mean ± SEM (**p<0.01, ***p<0.001, n=3; two-sided Student's t-test). Exact p-values are **(a)** con vs 2.5 p=0.0028, con vs 5 p=0.0005, con vs 10 p=0.0017, con vs 20 p=0.0012, **(d)** left: con vs 1 p<0.0001, con vs 3 p=0.0002, con vs 6 p=0.4717, con vs 9 p=0.6181, right: con vs 1 p<0.0001, con vs 3 p=0.0002, con vs 6 p=0.8863, con vs 9 p=0.4795.

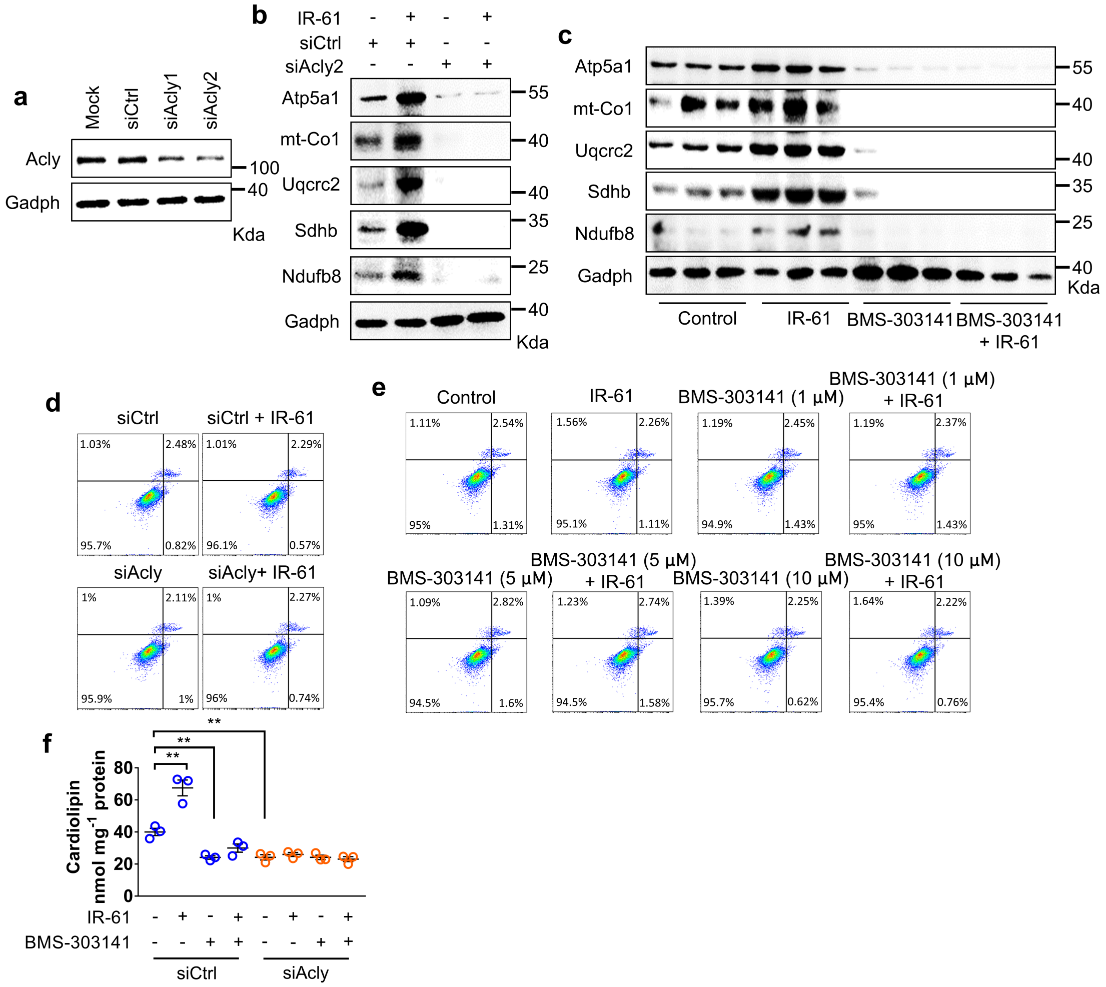

Supplementary Figure 10. Acly mediates the regulation of IR-61 on mitochondrial function. (a) Western blots of Acly levels in BMDMs transfected with siRNA control (siCtrl), Acly siRNA1 (siAcly1), and Acly siRNA2 (siAcly2). **(b)** Immunoblots of the respiratory chain subunits in whole cell lysates from the IR-61-treated BMDMs for 72 h after transfection with or without siAcly2. **(c)** Mice were intraperitoneally injected with vehicle, IR-61, BMS-303141, and BMS-303141 + IR-61. ATMs were isolated from SVF cells 2 days later and then mitochondrial complex content was detected by Western blots. **(d)** BMDMs were treated with IR-61 for 48 h after transfection with siCtrl or siAcly and then stained with Annexin-V/PI to detect the mode of death. **(e)** BMDMs were incubated with 10 μM IR-61 and various concentrations of BMS-303141 (0, 1 μM, 5 μM and 10μM) for 72 h and then stained with Annexin-V/PI to detect the mode of death. **(f)** Mitochondrial cardiolipin content of control and Acly knockdown BMDMs treated with IR-61 in the absence or with BMS-303141. Data are representative result of three independent experiments. Results are presented as the mean ± SEM (**p<0.01, n=3; two-sided Student's t-test). Exact p-values are **(f)** p=0.0071, p=0.0033, p=0.0049.

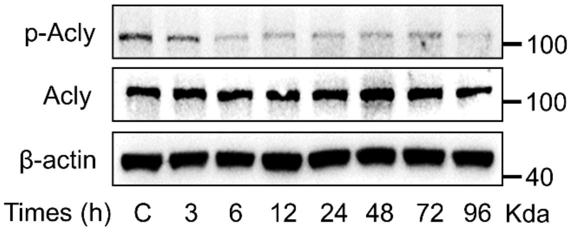

Supplementary Figure 11. Mice were intraperitoneally injected with BMS-303141. ATMs were isolated from SVF cells at the indicated time and then levels of Acly and p-Acly were detected by Western blots. Panels are representative result of three independent experiments.

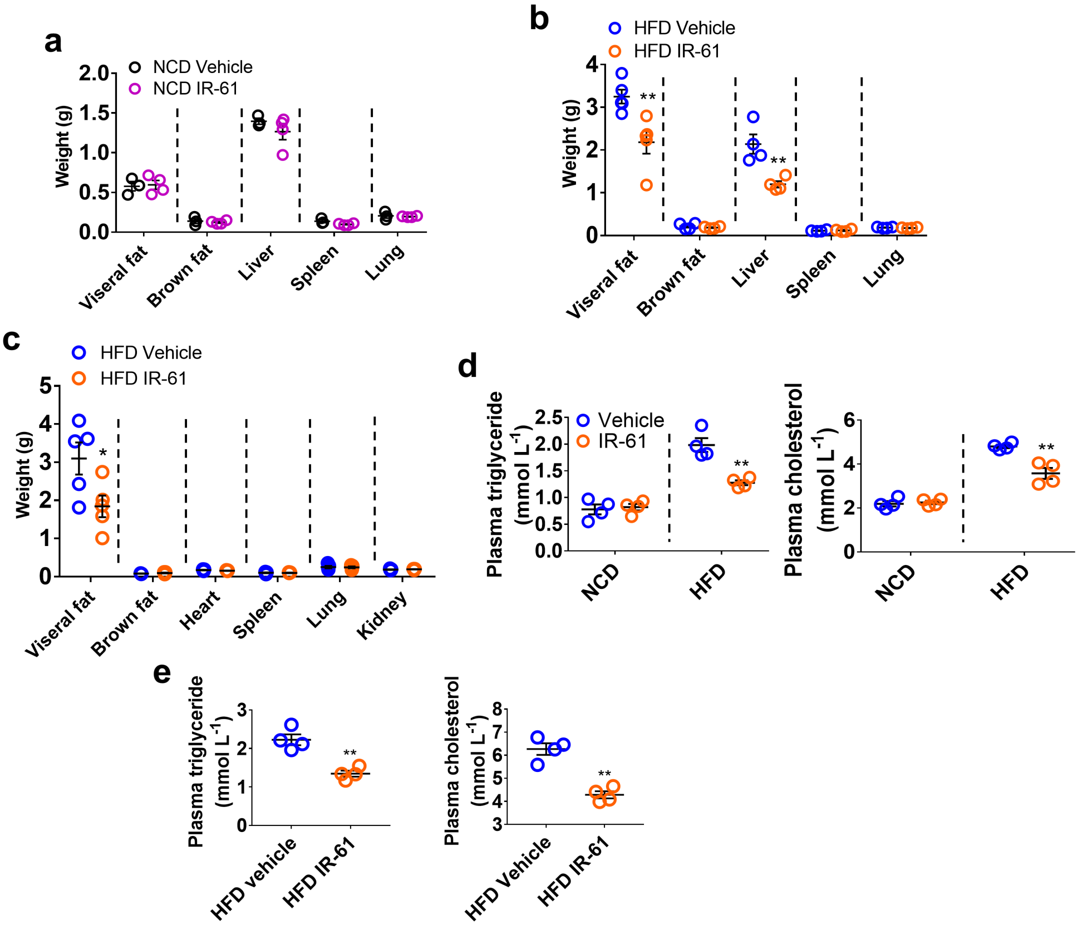

Supplementary Figure 12. The effect of IR-61 on organs weight and lipid metabolism of mice. (a) Organs weight of NCD-fed mice treated with IR-61 or vehicle control. **(b)** Organs weight of HFD-fed mice treated with IR-61 or vehicle control preventatively. **(c)** Organs weight of the mice with established obesity. **(d)** Measurement of serum triglyceride and cholesterol of mice on NCD or HFD concurrently treated with IR-61 or vehicle control. **(e)** Measurement of serum triglyceride and cholesterol in the mice with established obesity treated by IR-61 or vehicle control. Sample sizes are **(a)** *n*=3/4 mice, **(b)** visceral fat *n*=5/5 mice, brown fat

n=4/4 mice, liver *n*=4/4 mice, spleen *n*=4/4 mice, lung *n*=4/4 mice, **(c)** *n*=5/5 mice, **(d)** *n*=4/4 mice, **(e)** *n*=4/4 mice. Results are presented as the mean ± SEM (*$p<0.05$, **$p<0.01$, ***$p<0.001$; two-sided Student's *t*-test). Exact *p*-values are **(b)** visceral fat p=0.0092, brown fat p=0.3785, liver p=0.0077, spleen p=0.7569, lung p=0.3269, **(c)** visceral fat p=0.0386, brown fat p=0.2726, heart p=0.0982, spleen p=0.8342, lung p=0.8956, kidney p=0.4690, **(d)** left: p=0.7205, p=0.0020, right: p=0.6851, p=0.0031, **(e)** p=0.0015, p=0.0005.

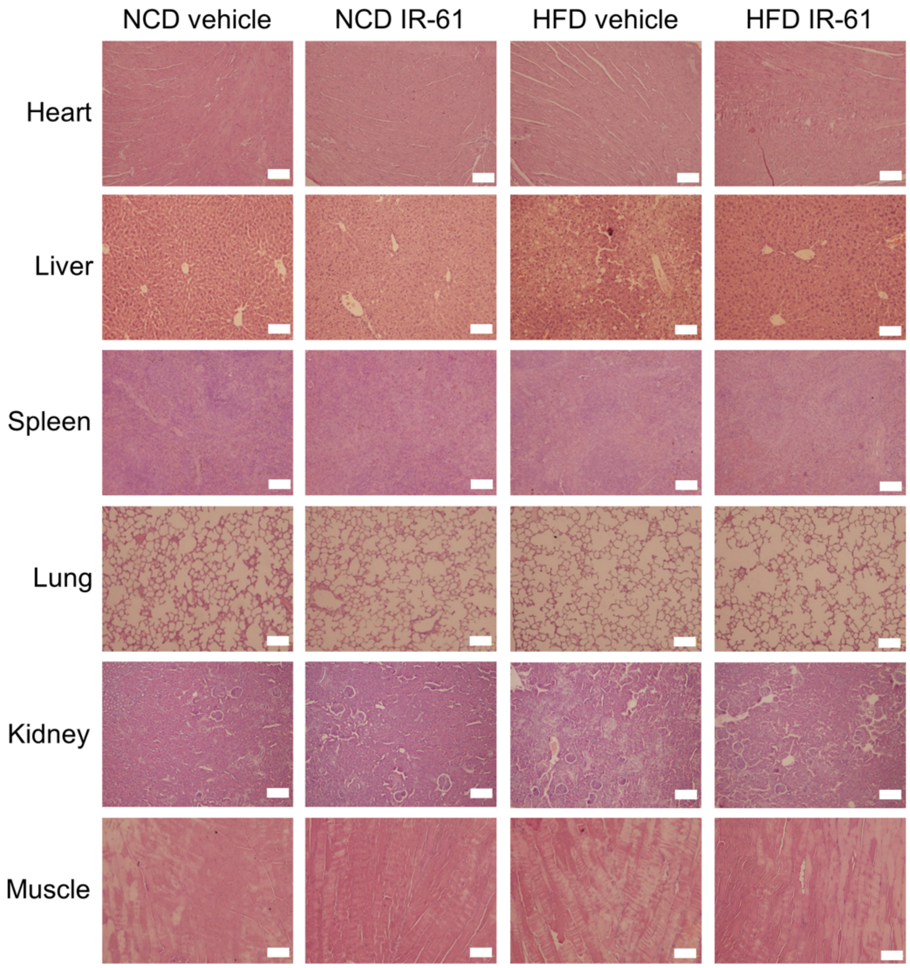

Supplementary Figure 13. Histopathologic analysis of vital organs in mice treated with IR-61 (Scale bars, 100 μm). *n*=3 per group.

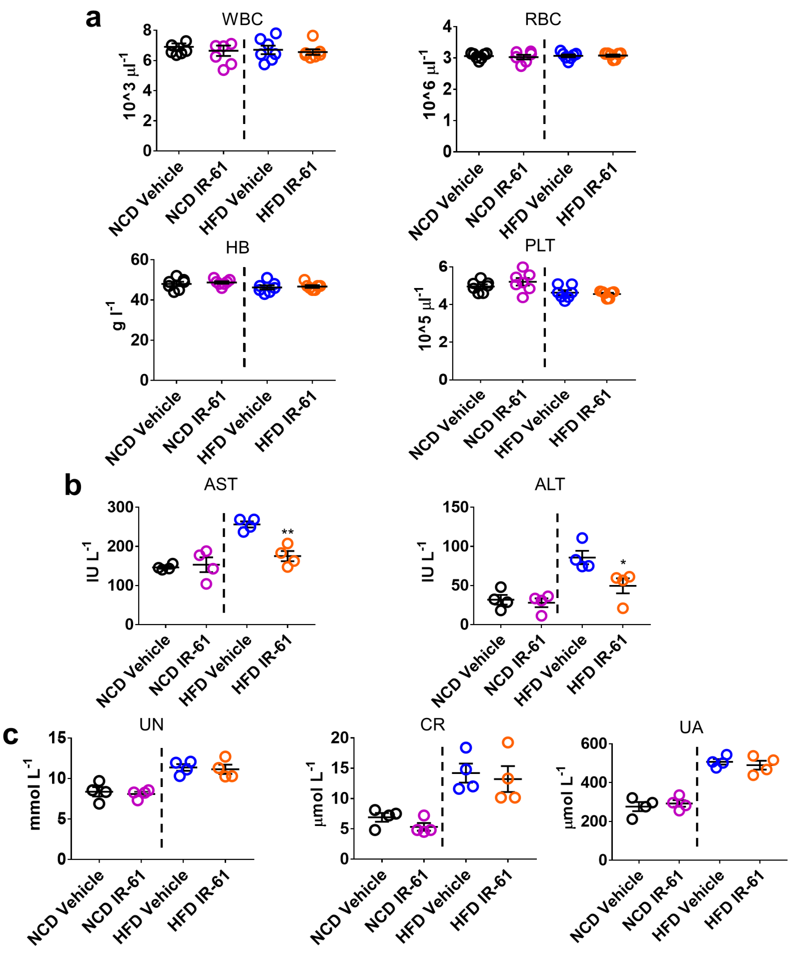

Supplementary Figure 14. Routine blood and serum biochemical parameters of the mice. (a) IR-61 had no effect on levels of WBC, RBC, HB and PLT in mice after 6 weeks treatment. **(b, c)** Serum biochemical parameters measurements for liver/kidney functions. Sample sizes are **(a)** WBC, HB, PLT *n*=7/7/7/7 mice, RBC *n*=7/6/7/7 mice, **(b)** *n*=4/4/4/4 mice, **(c)** *n*=4/4/4/4 mice. Results are presented as the mean ± SEM (**p*<0.05, ***p*<0.01; two-sided Student's *t*-test). Exact *p*-values are **(b)** AST *p*=0.7346, *p*=0.0018, ALT *p*=0.6633, *p*=0.0308.

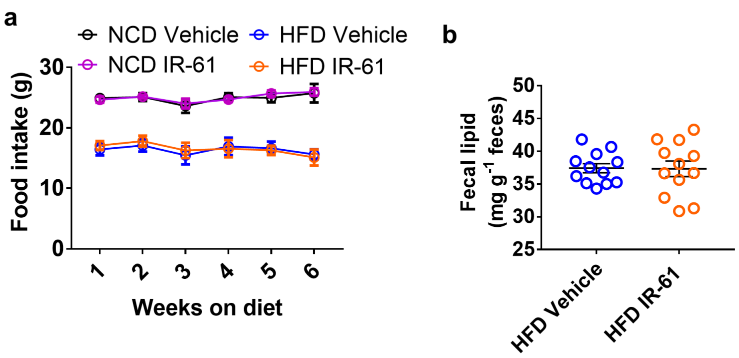

Supplementary Figure 15. IR-61 has no effect on food intake and intestinal lipid absorption. (a) Food intake of NCD-fed mice and HFD-fed mice treated with IR-61 or vehicle control. Food intake of each mouse was weighed once a week for six consecutive weeks. *n*=4 per group. **(b)** Levels of fecal lipid of HFD-fed mice treated with IR-61 or vehicle. Data were collected daily from 4 mice per group for 3 consecutive days. The ratios of the amount of daily total fecal lipid to daily feces weight of HFD-fed mice treated by IR-61 or vehicle were shown. Results are presented as the mean ± SEM (2-way ANOVA with Bonferroni post hoc test **(a)** or two-sided Student's *t*-test **(b)**). Exact *p*-values are **(a)** NCD vehicle vs NCD IR-61 1w $p>0.9999$, 2w $p>0.9999$, 3w $p>0.9999$, 4w $p>0.9999$,5w $p>0.9999$, 6w $p>0.9999$; HFD vehicle vs HFD IR-61 1w $p>0.9999$, 2w $p>0.9999$, 3w $p>0.9999$, 4w $p>0.9999$,5w $p>0.9999$, **(b)** $p=0.9485$.

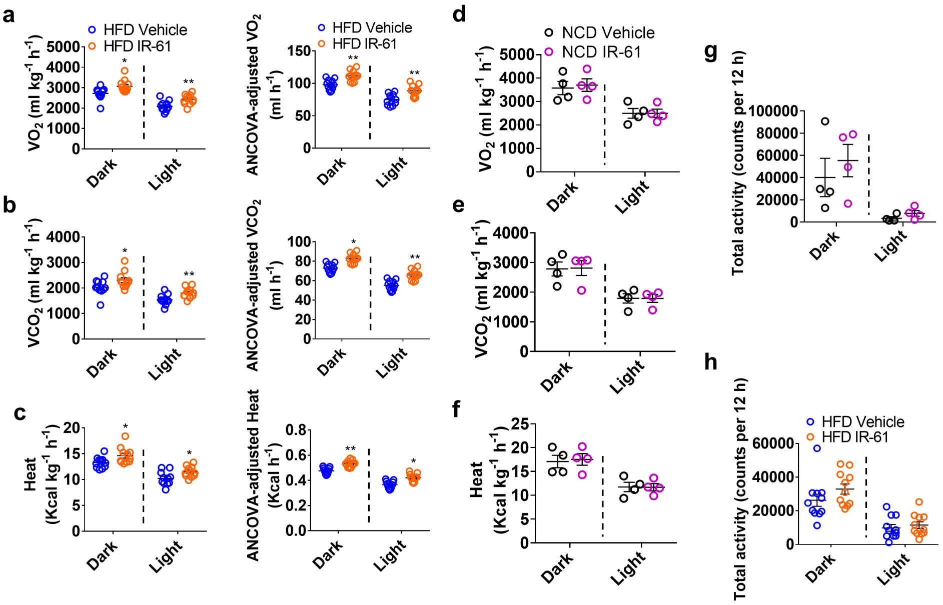

Supplementary Figure 16. The effect of IR-61 on energy expenditure and activity of mice. NCD-fed and HFD-fed mice were treated with IR-61 or vehicle control for 2 weeks and then the energy expenditure and total activity were monitored. **(a-c)** VO_2, VCO_2 and heat production in IR-61-treated or vehicle-treated HFD-fed mice were normalized by diving body weight or adjusted for variation in body weight using ANCOVA. **(d-f)** Average day and night VO_2, VCO_2 and heat production in IR-61-treated or vehicle-treated NCD-fed mice were obtained by indirect calorimetry. **(g)** Total activity in day and night of NCD-fed mice. **(h)** Total activity in day and night of HFD-fed mice. Sample sizes are **(a-c)** *n*=11/11 mice, **(d-g)** *n*=4/4 mice, **(h)** *n*=11/11 mice. Results are presented as the mean ± SEM (*$p<0.05$, **$p<0.01$, ***$p<0.001$; ANCOVA **(a-c)** or two-sided Student's *t*-test **(a-h)**). Exact *p*-values are **(a)** left: $p=0.0141$, $p=0.0054$, right $p=0.008$, $p=0.007$, **(b)** left: $p=0.0472$, $p=0.0051$, right $p=0.026$, $p=0.004$, **(c)** left: $p=0.0299$, $p=0.0193$, right $p=0.005$, $p=0.013$.

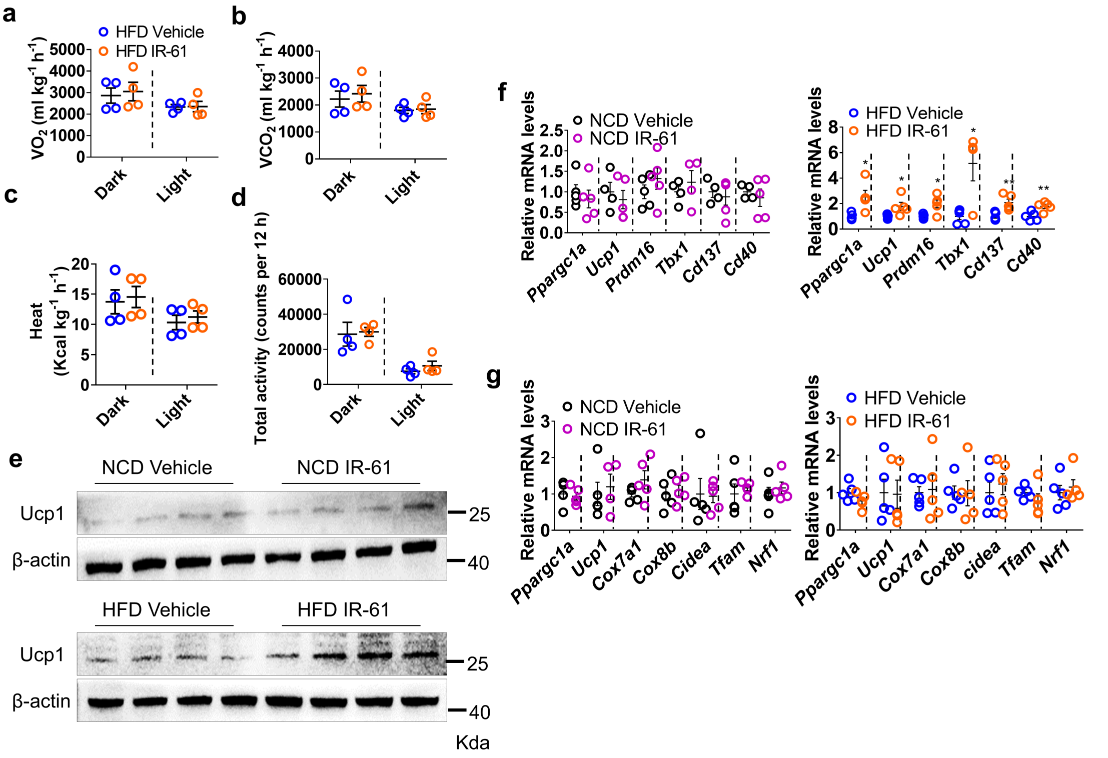

Supplementary Figure 17. The mechanism of IR-61 promoting energy expenditure in mice. (a-c) Average day and night VO_2, VCO_2 and heat production in HFD-fed mice treated with IR-61 or vehicle control for 1 day. **(d)** Total activity in day and night of HFD-fed mice. **(e)** immunoblot analysis of Ucp1 and β-actin in the iWAT of NCD-fed and HFD-fed mice treated with IR-61 or vehicle control for 2 weeks. **(f)** Expression of the thermogenic genes and beiging marker genes in the epi WAT of NCD-fed and HFD-fed mice treated with IR-61 or vehicle control for 2 weeks. **(g)** Expression of BAT activity genes in the BAT of NCD-fed and HFD-fed mice treated with IR-61 or vehicle control for 2 weeks. Sample sizes are **(a-d)** *n*=4/4 mice, **(e)** representative western blot of *n*=4 mice in each group, **(f)** left: *Ppargc1a n=5/5 mice, Ucp1 n*=4/5 mice, *Prdm16 n*=5/5 mice, *Tbx1 n*=4/4 mice, *Cd137 n*=4/5 mice, *Cd40 n*=4/5 mice; right *Ppargc1a n*=5/5 mice, *Ucp1 n*=5/5 mice, *Prdm16 n*=5/5 mice, *Tbx1 n*=5/4 mice, *Cd137 n*=5/5 mice, *Cd40 n*=5/5 mice, **(g)** left: *Ppargc1a n=5/5 mice, Ucp1 n*=5/4 mice, *Cox7a1 n*=5/5 mice, *Cox8b n*=5/5 mice, *Cidea n*=5/5 mice, *Tfam n*=5/5 mice, *Nrf1 n*=5/5 mice; right: *Ppargc1a n*=5/5 mice, *Ucp1 n*=5/5 mice, *Cox7a1 n*=5/5 mice, *Cox8b n*=5/5 mice, *Cidea n*=5/5 mice, *Tfam n*=5/5 mice, *Nrf1 n*=5/5 mice. Results are presented as the mean ± SEM (**p*<0.05, ***p*<0.01; two-sided Student's *t*-test). Exact *p*-values are **(f)** left: *Ppargc1a p=0.5428, Ucp1 p*=0.5757, *Prdm16 p*=0.3298, *Tbx1 p*=0.4894, *Cd137 p*=0.6565, *Cd40 p*=0.5840; right *Ppargc1a p*=0.0148, *Ucp1 p*=0.0405, *Prdm16 p*=0.0146, *Tbx1 p*=0.0119, *Cd137 p*=0.0073, *Cd40 p*=0.0081.

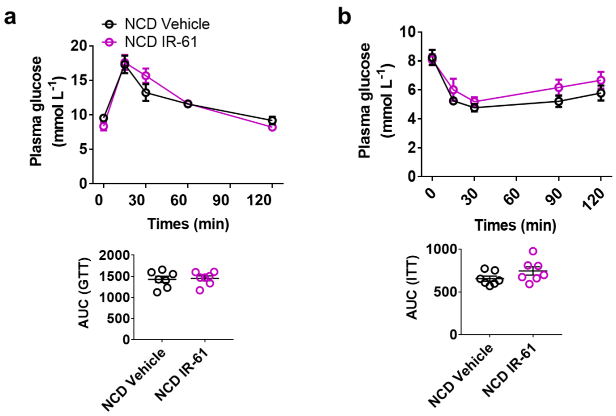

Supplementary Figure 18. IR-61 does not affect insulin sensitivity of the mice fed on NCD (a) GTT and **(b)** ITT on NCD-fed mice treated with IR-61 or vehicle control for 15 weeks. *n*=7 per group. Data are presented as the mean ± SEM (2-way ANOVA with Bonferroni post hoc test or two-sided Student's *t*-test).

Supplementary Table 1: Primers used for real time PCR

Gene	Sense	Anti-sense
Tnf	TTCTCATTCCTGCTTGTG	TTGGTGGTTTGCTACG
Il6	AGTCAATTCCAGAAACCGCTATGA	TAGGGAAGGCCGTGGTTGT
Il1b	TGCCACCTTTTGACAGTGATG	ATGTGCTGCTGCGAGATTTG
Cxcl1	CAGAGCTTGAAGGTGTTGC	AGTGTGGCTATGACTTCGG
Cxcl2	TCAATGCCTGAAGACCCTG	AGAGTGGCTATGACTTCTGTC
Cxcl3	ACAGAAGTCATAGCCACTCTC	CTTGATGATTATCTGAAGCCTGG
Cd14	AATCTACCGACCATGGAGC	ACTTTCCTCGTCTAGCTCG
Cd69	TCTCATTGCCTTAAATGTGGG	GTAGCAACATGGTGGTCAG
Tlr2	AGACAAAGCGTCAAATCTCAG	GCTGAAGAGGACTGTTATGG
Tlr11	GCTCAAAGAATCGATGCCA	CTCACCAGAGTCAATAGTATCAG
Arg1	CCACAGTCTGGCAGTTGGAA	GCATCCACCCAAATGACACA
Mrc1	AATACCTTGAACCCATTTATCATTC C	GCATAGGGCCACCACTGATT
Fizz1	CGTGGAGAATAAGGTCAAGGAACT	CACTAGTGCAAGAGAGAGTCTT CGTT
Ym1	TCCTACTGGAAGGACCATGGAGCA	TCCTGGTGGGCCAGTACTAATT GT
Ndufb8	ATGCGAGTGGAAGACTACG	TCCCAGTGTATCGGTTCAC
Sdhb	ACCCCTTCTCTGTCTACCG	AATGCTCGCTTCTCCTTGTAG
Uqcrc2	GTGGGAAAGGGCAACTGCTA	GTCCCATGCTCACCGAGAAG
mt-Co1	ACTATACTACTAACAGACCG	GGTTCTTTTTTTCCGGAGTA
Atp5a1	CATTGGTGATGGTATTGCGC	TCCCAAACACGACAACTCC
Ppara	AGAGGGCTGAGCGTAGGTAA	CCTCCGATCACCCCCATTTC

Pparg	GCCCTTTGGTGACTTTATGGA	GCAGCAAGGTTGTCTTGGATG
Fasn	GGAGGTGGTGATAGCCGGTAT-	TGGGTAATCCATAGAGCCCAG
Mcad	GAACCAGACCTACAGTCGCA	GCTCCACTAGCAGCTTTCCA
HSL	TGCACTCTACCACTGCCTTC	GCTGGGAAAACACACTCCCT
Atgl	CCACTCACATCTACGGAGCC	ACAGCAGACAGTAGGCAACC
Acc1	GACAGACTGATCGCAGAGAAAG	TGGAGAGCCCCACACACA
Srebf1	CTGGCACTAAGTGCCCTCAAC	GCCACATAGATCTCTGCCAGTGT
Cpt1b	AAGAGACCCCGTAGCCATCAT	GACCCAAAACAGTATCCCAATCA
Lcad	CAAACGTCTGGACTCCGGTT	ACGTAAGCTTTTGCAATCGGG
Sirt1	ACGGTATCTATGCTCGCCTTG	GACACAGAGACGGCTGGAAC
Ppargc1a	TCTGAGTCTGTATGGAGTGACAT	CCAAGTCGTTCACATCTAGTTCA
Nrf1	ACAGATAGTCCTGTCTGGGGAAA	TGGTACATGCTCACAGGGATCT
Ucp-1	TCAACACTTTGGAAAGGGAC	GAGGTCATATGTTACCAGCTC
Cidea	TTCTTTCAGACCTTAAGGGAC	GACATACTTACTACCCGGTG
Prdm16	CCATACAGGTGCAAGTACTG	TTTGTTGTGGATGTTCCTCAC
Tbx1	GTGGATGAAACAGATTGTGTC	GGAGTTGAGAATAATATGGCCA
Cd137	GTGTGCAGGCTATTTCAGG	CAATGGAATCCTTCAATGCAC
Cd40	CACTGTGAACCCAATCAAGG	TCCTTCCTTACAGGTACAGAC
Cox7a1	GACAACGTCCTGTACAGAC	ATAAGCAGTAGGCAGTGCC
Cox8b	AAGTTCACAGTGGTTCCCA	CGACTATGGCTGAGATCCC
Tfam	AAGACCTCGTTCAGCATATAACATT	TTTTCCAAGCCTCATTTACAAGC
β-actin	CTGTCCCTGTATGCCTCTG	ATGTCACGCACGATTTCC
