# Supplementary material for: Improvement of obesity-associated disorders by a small-molecule drug targeting mitochondria of adipose tissue macrophages
Source: Nat Commun. 2021 Jan 4;12:102. doi: 10.1038/s41467-020-20315-9 (PMC7782823; doi:10.1038/s41467-020-20315-9)
Supplement: Supplementary file 3 — Reporting Summary [file 41467_2020_20315_MOESM3_ESM.pdf]

nature research

Corresponding author(s): Chunmeng Shi, Hongming Miao and Yu Wang

Last updated by author(s): 22/10/20

Reporting Summary

Nature Research wishes to improve the reproducibility of the work that we publish. This form provides structure for consistency and transparency in reporting. For further information on Nature Research policies, see our Editorial Policies and the Editorial Policy Checklist.

Statistics

For all statistical analyses, confirm that the following items are present in the figure legend, table legend, main text, or Methods section.

n/a	Confirmed	
☐	☒	The exact sample size (n) for each experimental group/condition, given as a discrete number and unit of measurement
☐	☒	A statement on whether measurements were taken from distinct samples or whether the same sample was measured repeatedly
☐	☒	The statistical test(s) used AND whether they are one- or two-sided<br>*Only common tests should be described solely by name; describe more complex techniques in the Methods section.*
☒	☐	A description of all covariates tested
☒	☐	A description of any assumptions or corrections, such as tests of normality and adjustment for multiple comparisons
☐	☒	A full description of the statistical parameters including central tendency (e.g. means) or other basic estimates (e.g. regression coefficient) AND variation (e.g. standard deviation) or associated estimates of uncertainty (e.g. confidence intervals)
☐	☒	For null hypothesis testing, the test statistic (e.g. F, t, r) with confidence intervals, effect sizes, degrees of freedom and P value noted<br>*Give P values as exact values whenever suitable.*
☒	☐	For Bayesian analysis, information on the choice of priors and Markov chain Monte Carlo settings
☒	☐	For hierarchical and complex designs, identification of the appropriate level for tests and full reporting of outcomes
☒	☐	Estimates of effect sizes (e.g. Cohen's d, Pearson's r), indicating how they were calculated

Our web collection on statistics for biologists contains articles on many of the points above.

Software and code

Policy information about availability of computer code

Data collection	LabImage version 3.0 (BioRad), Real-Time System (BioRad),
Data analysis	Analyses were performed on SPSS 26.0, GraphPad Prism 8.4.3, Image J version 1.52, FlowJo V10.1, CFlow Plus1.02

For manuscripts utilizing custom algorithms or software that are central to the research but not yet described in published literature, software must be made available to editors and reviewers. We strongly encourage code deposition in a community repository (e.g. GitHub). See the Nature Research guidelines for submitting code & software for further information.

Data

Policy information about availability of data

All manuscripts must include a data availability statement. This statement should provide the following information, where applicable:

- Accession codes, unique identifiers, or web links for publicly available datasets
- A list of figures that have associated raw data
- A description of any restrictions on data availability

The authors declare that all data supporting the findings of this study are available within the paper and its Supplementary Information files, and from the authors on request.

Field-specific reporting

Life sciences study design

All studies must disclose on these points even when the disclosure is negative.

Sample size	Sample sizes were determined based on our previous publication (Hongming Miao, et. al. Cell Rep. 2014 ;7(1):223-35) and published data reported elsewhere associated with obesity-related diseases (Jialin Li, et. al. Nat Commun. 2017 ;8(1):1322), with assays on related projects and variability of the response deviating from the mean as presented in the graphs and figure legends. Sample sizes and statistical data are reported in figure legends.
Data exclusions	No data was excluded.
Replication	All experiments were repeated at least three times with reproducibility. The replication number is indicated in the legend of the corresponding figure
Randomization	Animals were randomly assigned to experimental groups. Cell culture experiments were handled the same way.
Blinding	H&E sections analyses were done in a blinded fashion by sending the pathological sections to a pathologist without associated information, which was mentioned in Methods. For the other experiments, the investigators were not blinded to the experimental conditions since animals or cells need to be treated by investigators and there are no subjective measures for the analyses.

Reporting for specific materials, systems and methods

We require information from authors about some types of materials, experimental systems and methods used in many studies. Here, indicate whether each material, system or method listed is relevant to your study. If you are not sure if a list item applies to your research, read the appropriate section before selecting a response.

Materials & experimental systems

n/a	Involved in the study	
☐	☒	Antibodies
☐	☒	Eukaryotic cell lines
☒	☐	Palaeontology and archaeology
☐	☒	Animals and other organisms
☒	☐	Human research participants
☒	☐	Clinical data
☒	☐	Dual use research of concern

Methods

n/a	Involved in the study	
☒	☐	ChIP-seq
☐	☒	Flow cytometry
☒	☐	MRI-based neuroimaging

Antibodies

Antibodies used	Anti-AKT (#4685, Cell Signaling), Anti-Phospho-AKT (Thr308) (#2965, Cell Signaling), Anti-NF-κb p65 (#8242, Cell Signaling), Anti-Phospho-p65 (#3033, Cell Signaling), Anti-SAPK/JNK (#9252, Cell Signaling), Anti-Phospho-SAPK/JNK (Thr183/Tyr185) (#4668, Cell Signaling), Anti-total OXPHOS cocktail (#ab110413, Abcam), Anti-ATP citrate lyase (#ab40793, Abcam), Anti-Phospho-ATP citrate lyase (Ser455) (#4331, Cell Signaling), Anti-AMPK (#5831, Cell Signaling), Anti-Phospho-AMPK (#2535, Cell Signaling), Anti-ACC (#3676, Cell Signaling), Anti-Phospho-ACC (#11818, Cell Signaling), Anti-UCP1 (#23673-1-AP, Proteintech), Anti-β-Actin (#AF0003, Beyotime), Anti-GADPH (#1E6D9, Proteintech), PerCP/Cyanine5.5 anti- mouse CD45 (#103131, Biolegend), Brilliant Violet 421 anti-mouse F4/80 (#123131, Biolegend), APC anti-mouse CD11C (#117310, Biolegend), rat anti-mouse F4/80 antibody (#123101, Biolegend), goat anti-rat Alexafluor 488 secondary antibody (#A-11006, Invitrogen)
Validation	Antibodies were chosen based on previous literature. Validation and quality control is available from the manufacturers using the catalog number of each antibody.<br>#4685, Cell Signaling is recommended by the manufacturer for detection of AKT in human, mouse and rat monkey samples. Validation according to the manufacturers website using western blot analysis of extracts from HeLa, C2C12, C6 and COS cells. Product citations include Mengtian Fan, et. al. Oncol Rep. 2020; 44:91-102; Katharina Timper, et. al. Cell Metab . 2020; 31:1189-1205.e13.<br>#2965, Cell Signaling is recommended by the manufacturer for detection of Akt only when phosphorylated at Thr308 in human, mouse, rat and monkey samples. Validation according to the manufacturers website using western blot analysis of extracts from NIH/3T3 and Jurkat cells, untreated, PDGF-treated or LY294002-treated as indicated, using Phospho-Akt (Thr308) (C31E5E) Rabbit mAb or Akt (pan) (C67E7) Rabbit mAb #4691. Product citations include Dejiang Pang, et. al. Aging (Albany NY). 2020; 12:8710-8727; Laura Mosca, et. al. Int J Oncol. 2020 ;56:1212-1224.<br>#8242, Cell Signaling is recommended by the manufacturer for detection of endogenous levels of total NF-κB p65/RelA protein in human, mouse, rat and monkey samples. Validation according to the manufacturers website using western blot analysis of extracts from various cell lines using NF-κB p65 (D14E12) XP® Rabbit mAb. Product citations include Guohua Zhu, et. al. Pharm Biol. 2020 ;58:25-34; Meiling Wang, et. al. Front Pharmacol. 2020 ;11:515.<br>#3033, Cell Signaling is recommended by the manufacturer for detection of NF-κB p65 only when phosphorylated at Ser536 in human, mouse, rat, pig and monkey samples. Validation according to the manufacturers website using Western blot analysis of extracts from HeLa and NIH/3T3 cells, untreated or TNF-α treated (#2169, 20 ng/ml for 5 minutes), using Phospho-NF-κB p65

(Ser536) (93H1) Rabbit mAb or NF-κB p65 Antibody #3034. Product citations include Ki-Back Chu, et. al. Cells. 2020;9:1314; Anna Paszek, et. al. Cell Commun Signal. 2020 ; 18:77.

#9252, Cell Signaling is recommended by the manufacturer for detection of endogenous levels of total JNK1, JNK2 or JNK3 protein in human, mouse, rat, Hamster and monkey samples. Validation according to the manufacturers website using Western blot analysis of extracts from 293 and SK-N-MC cells, untreated or UV-treated (40 J/m2), using Phospho-SAPK/JNK Antibody #9251 or SAPK/JNK Antibody . Product citations include Jae Hyuk Lee, et. al. Cell Death Dis. 2020 ;11:403; Yangfang Ding, et. al. Oxid Med Cell Longev . 2020 ;2020:3237150.

#4668, Cell Signaling is recommended by the manufacturer for detection of endogenous levels of p46 and p54 SAPK/JNK when phosphorylated at Thr183 and Tyr185 in human, mouse, rat, Hamster and S. cerevisiae. Validation according to the manufacturers website using Western blot analysis of extracts from 293 cells, untreated or UV-treated, NIH/3T3 cells, untreated or UV-treated and C6 cells, untreated or anisomycin-treated, using Phospho-SAPK/JNK (Thr183/Tyr185) (81E11) Rabbit mAb. Product citations include Yandong Li, et. al. Cell Death Dis. 2020 ;11:299; Guang-Jie Liu, et. al. Front Neurosci. 2020 ;14:311.

#ab110413, Abcam is recommended by the manufacturer for detection of endogenous levels of the 5 OXPHOS complexes in mitochondrial preparations from mouse, rat, human, or bovine sources. Validation according to the manufacturers website using Western blot analysis of extracts from Isolated mitochondria from mice brain (control and Alzheimer's disease (AD)) labeled with ab110413 at 1/1000 dilution in 5% BSA. Product citations include Feng J et al. J Exp Clin Cancer Res 39:24 (2020); Svensson K et al. Am J Physiol Endocrinol Metab 318:E145-E151 (2020).

#ab40793, Abcam is recommended by the manufacturer for detection of endogenous levels of ATP citrate lyase (ACL) in mouse, rat, human samples. Validation according to the manufacturers website using Western blot analysis of extracts from NIH/3T3 (Mouse embryonic fibroblast). Product citations include Jin F et al. Cancer Lett 440-441:211-222 (2019); Shi Z et al. Life Sci 217:70-80 (2019).

#4331, Cell Signaling is recommended by the manufacturer for detection of endogenous levels of ATP-citrate lyase only when phosphorylated at Ser455 in human and mouse samples. Validation according to the manufacturers website using Western blot analysis of extracts from NIH/3T3 cells, untreated or PDGF-treated for the indicated times, using Phospho-ATP-Citrate Lyase (Ser455) Antibody or ATP-Citrate Lyase Antibody #4332. Product citations include C Martínez Calejman, et. al. Nat Commun . 2020 ;11:575; Jian Wang, et. al. Clin Sci (Lond). 2019 ;133:2189-2202.

#5831, Cell Signaling is recommended by the manufacturer for detection of endogenous levels of AMPKα protein in human, rat, monkey and mouse samples. Validation according to the manufacturers website using Western blot analysis of extracts from HeLa, K-562, C6, and Neuro-2a cells using AMPKα (D5A2) Rabbit mAb. Product citations include Chunsheng Liu, et. al. Oncol Rep . 2020 ;43:1467-1478; Denise Peserico, et. al.Antioxidants (Basel). 2020 ;9:349.

#2535, Cell Signaling is recommended by the manufacturer for detection of endogenous levels of AMPKα only when phosphorylated at threonine 172 in human, rat, monkey, mouse and S. cerevisiae samples. Validation according to the manufacturers website using Western blot analysis of extracts from C2C12 cells, untreated or oligomycin-treated (0.5 μM), using Phospho-AMPKα (Thr172) (40H9) Rabbit mAb or AMPKα Antibody #2532. Product citations include Gang Ren, et. al. Front Pharmacol. 2020 ;11:647; Bo Yu, et. al. Nat Commun. 2020 ;11:2549.

#3676, Cell Signaling is recommended by the manufacturer for detection of endogenous levels of all isoforms of acetyl-CoA carboxylase protein in human, rat, mouse and hamster samples. Validation according to the manufacturers website using Western blot analysis of extracts from various cell lines, using Acetyl-CoA Carboxylase (C83B10) Rabbit mAb. Product citations include Kristell Oizel, et. al. Cell Death Dis. 2020 ;11:310; Kai Meng, et. al. Oxid Med Cell Longev. 2020; 2020:1645249.

#11818, Cell Signaling is recommended by the manufacturer for detection of endogenous levels of acetyl-CoA carboxylase protein only when phosphorylated at Ser79 in human, rat and mouse samples. Validation according to the manufacturers website using Western blot analysis of extracts from SH-SY5Y cells, untreated or treated with Oligomycin #9996 (0.5 μM, 30 min), using Phospho-Acetyl-CoA Carboxylase (Ser79) (D7D11) Rabbit mAb or Acetyl-CoA Carboxylase (C83B10) Rabbit mAb #3676. Product citations include Qunyan Yao, et. al. Ann Transl Med. 2020 ;8:231; Simon A Hawley, et. al; Cell Chem Biol. 2020 ;27(:214-222.e4.

#23673-1-AP, Proteintech is recommended by the manufacturer for detection of endogenous levels of ucp1 in Human, Mouse and Rat in samples. Validation according to the manufacturers website using Western blot analysis of extracts from mouse brown adipose tissue. Product citations include Michelle Chan, et, al. J Biol Chem. 2019 ;294:6751-6761; Yan Zhao, et, al. Theranostics . 2019 ;9:1510-1522.

#1E6D9, Proteintech is recommended by the manufacturer for detection of endogenous levels of Gadph in Human, Mouse, Rat, Yeast, Plant samples. Validation according to the manufacturers website using Western blot analysis of extracts from HeLa cells, soybean whole plant tissue, arabidopsis whole plant tissue, HepG2 cells, ROS1728 cells, pig brain tissue, zebrafish tissue, whole yeast cells, whole Nematode tissue, HEK-293 cells, HSC-T6 cells, PC-12 cells, NIH/3T3 cells, C2C12 cells, SP2/0 cells, rat brain tissue, mouse brain tissue. Product citations include Anni Tan, et, al. FASEB J. 2019;33:4893-4906; Alan Costello, et, al. Metab Eng. 2019 ;52:284-292.

#AF0003, Beyotime is recommended by the manufacturer for detection of endogenous levels of β-Actin in human, mouse and rat samples. Validation according to the manufacturers website using Western blot analysis of extracts from Hela, NIH/3T3 and PC12 cells. Product citations include Ding L , et, al. Mol Med Rep . 2016 ;13:2017-22; Wang Y , et, al. Oncol Lett . 2016;12:2429-2434

#103131, Biolegend is recommended by the manufacturer for flow cytometry in mouse. Validation according to the manufacturers website by detection of CD45 of splenocytes in C57BL/6 mouse. Product citations include Podd BS, et al. 2006. J. Immunol. 176:6532.; Liu Z, et al. 2015. Development. 142: 2452 - 2463.

#123131, Biolegend is recommended by the manufacturer for flow cytometry in mouse. Validation according to the manufacturers website by detection of F4/80 of thioglycolate-elicited Balb/c mouse peritoneal macrophages. Product citations include Watson NB, et al. 2015. J Immunol. 194:2796; Müller P, et al. 2015. Sci Transl Med. 7: 315ra188.

#117310, Biolegend is recommended by the manufacturer for flow cytometry in mouse. Validation according to the manufacturers website by detection of CD11C of C57BL/6 mouse splenocytes. Product citations include Riva A, et al. 2013. J Immunol. 190:5961; Madan-Lala R, et al. 2014. J Immunol. 192:4263.

#123101, Biolegend is recommended by the manufacturer for immunofluorescence in mouse. Product citations include Schaller E, et al. 2002. Mol. Cell. Biol. 22:8035; Stevceva L, et al. 2001. BMC Clin Pathol. 1:3.

#A-11006, Invitrogen is recommended by the manufacturer for immunofluorescence when using anti-rat primary antibody. Validation according to the manufacturers website by using A549 cells stained with alpha Tubulin (YL1/2) Rat Monoclonal Antibody.

Eukaryotic cell lines

Policy information about cell lines

Cell line source(s)	RAW264.7 cells (ATCC, USA)
Authentication	Authentication according to the manufacturer's website: RAW264.7 cell line was established from a tumor induced by Abelson murine leukemia virus. This cell line is easy to propagate, high efficiency for DNA transfection, sensitivity to RNA interference, and supports replication of murine noroviruses. This cell line is negative for surface immunoglobulin (sIg-), Ia (Ia-) and Thy-1.2 (Thy-1.2). When this line was established, it was described as not secreting detectable virus particles and negative using the XC plaque formation assay. Product related references include Ralph P, et, al. J. Immunol. 119: 950-954, 1977; Raschke WC, et, al. Cell 15: 261-267, 1978.
Mycoplasma contamination	Cell lines were not tested for mycoplasma contamination
Commonly misidentified lines (See ICLAC register)	No commonly misidentified cell lines were used in the study.

Animals and other organisms

Policy information about studies involving animals; ARRIVE guidelines recommended for reporting animal research

Laboratory animals	C57BL/6J mice (male, 6-8 weeks) were purchased from Laboratory Animal Center. The environmental conditions in the mouse facility were: 12h light and 12h dark cycle, temperature range of 21-23°C, humidity range of 40-50% and free access to food and water.
Wild animals	This study did not use wild animals.
Field-collected samples	This study did not involve collecting samples from the field
Ethics oversight	In vivo experiments were conducted in accordance with the Guidelines for the Care and Use of Laboratory Animals of Army Medical University (AMU), and all procedures were approved by the Animal Care and Use Committee of the AMU.

Note that full information on the approval of the study protocol must also be provided in the manuscript.

Flow Cytometry

Plots

Confirm that:

- [x] The axis labels state the marker and fluorochrome used (e.g. CD4-FITC).
- [x] The axis scales are clearly visible. Include numbers along axes only for bottom left plot of group (a 'group' is an analysis of identical markers).
- [x] All plots are contour plots with outliers or pseudocolor plots.
- [x] A numerical value for number of cells or percentage (with statistics) is provided.

Methodology

Sample preparation	Cultured cells and SVFs, splenocytes and liver cells were collected and single cell suspension were processed for staining.
Instrument	BD Accuri C6 and BD LSRFortessaTM cell analyzer were used for data collection.
Software	CFlow Plus or FlowJo 10 were used to collect and analyze the flow cytometry data, respectively.
Cell population abundance	For cultured cells, at least 10000 cultured cells were acquried per sample; for SVFs, splenocytes and liver cells, 100000-1000000 cells were acquired for further analysed.
Gating strategy	Experiments were gated first by morphology to exclude cell debrees, then in case of Fixable Viability Dye negative cells.

- [x] Tick this box to confirm that a figure exemplifying the gating strategy is provided in the Supplementary Information.
