# Supplementary material for: Improvement of obesity-associated disorders by a small-molecule drug targeting mitochondria of adipose tissue macrophages
Source: Nat Commun. 2021 Jan 4;12:102. doi: 10.1038/s41467-020-20315-9 (PMC7782823; doi:10.1038/s41467-020-20315-9)
Supplement: Supplementary file 2 — Peer Review File [file 41467_2020_20315_MOESM2_ESM.docx]

Reviewers' comments:

Reviewer #1 (Remarks to the Author):

This study shows that a newly developed infrared dye IR-61 has anti-inflammatory and anti-obesity effects in mice. This molecule has strong physiological effects, but the data shown is not sufficient to support the mechanism proposed via mitochondria ROS, Akt, and Acly.

Specific comments:
1. Mice treated with IR-61 have a very significant change in body mass compared to control animals without a change in food intake. The authors will need to investigate the mechanism for this effect. For example, indirect calorimetry to determine whether IR-61 alters energy expenditure and fecal nutrient content of lipids. Does the molecule induce beige fat or activate brown fat mitochondria?
2. Knockout of Acly is toxic and embryonic lethal in mice. The authors show that siRNA knockdown of Acly depletes mitochondrial ETC complexes and diminishes ATP-linked respiration even in the absence of IR-61 treatment. Therefore, the authors do not have sufficient evidence to conclude that Acly is required for IR-61-mediated changes in mitochondrial ETC/activity because the cellular system is significantly impaired by loss of Acly in the first instance. The small molecule BMS drug also wipes out mitochondrial ETC content.
3. Only one antioxidant (NAC) was tested at one concentration. NAC binds many small molecules. The authors should determine whether NAC directly interacts with IR-61 in a cell-free system. If so, then NAC is simply a sponge that decreases IR-61 availability and this result could markedly change interpretation of the data.
4. The authors do not mention how much NAC was used in the cell-based experiments.
5. Because NAC interacts with a large number of molecules, it is very important to define the minimum amount necessary to block IR-61 effects so that off-target actions can be minimized. For example, NAC treatment alone in Fig 4D blocks Akt and Acly phosphorylation in the absence of IR-61 – therefore, it is not appropriate for the authors to conclude that NAC blocks IR-61-induced phosphorylation of Akt and Acly.
6. Histology slides are shown, but there is no indication of whether they were evaluated by a histopathologist.
7. The absorption and emission spectra of IR-61 should be shown.
8. There is no indication of how mitochondrial membrane potential was assessed.
9. Can the authors show data to confirm that IR-61 is not interfering with fluorescent dyes DCFH-DA, MitoSOX Red, Mito-Tracker Green, and whatever dye was used to assess membrane potential (methods missing).
10. Mitochondria is misspelled in Fig 2G (as mytochondria).
11. Micromolar uses a capital M while micrometer uses a lowercase m.
12. The authors should justify why 10 micromolar dose of IR-61 was used. Better yet a dose response should be performed to experimentally show that mitoROS production correlates with the biological endpoints.
13. Regarding ATP levels in cells, can the authors confirm that IR-61 is not interfering in the assay? Do ADP and AMP also increase by a similar fold change? Whenever energy charge in a cell changes AMPK activity should be assessed.
14. Seahorse assay OCR rates are shown in pmol/min, but the correct way to express the data is normalized to cell number or protein content. In this instance the authors treat the cells while they are in the dish over a period of time when cell number and size can change, so the assays should be normalized to protein content.
15. The p-Acly site Ser455 is also a PKA substrate so the authors should test an Akt inhibitor and a PKA inhibitor in the presence of IR-61 to determine which kinase is involved in Ser455 phosphorylation.
16. Revise gene nomenclature appropriate to species, e.g. when referring to targeting mouse Acly gene with siRNA etc
17. Spelling error in 2.2 “IR-6 [sic] -treated cells”
18. The content and activity of mitochondrial ETC complexes are increased without a change in mitochondrial mass by MitoTracker staining or transcription factor expression changes. These are minimum supportive data but don’t tell the full story. To better understand the mechanisms linking IR-61 to mitochondrial ETC activity it would be good to show whether mtDNA changes, whether mitochondrial supercomplex formation is altered by using blue native PAGE, whether citrate synthase activity is altered.
19. Will any molecule with a free sulfhydryl group block IR-61 effects? Do other mitochondrial antioxidants (E.g. SS-31, MnTBAP, mitocatalase) block IR-61 effects?
20. If the effects of IR-61 are solely due to mitoROS signaling as proposed, then does a known control compound such as mito-paraquat have the same effects?
21. Is siRNA knockdown of Acly toxic – a measure of cell viability should be determined in the absence and presence of IR-61 (as was performed in Fig S6 in WT BMDM cells). A Ser455Ala point mutation would have been a better approach.
22. CT data is shown to measure fat mass in Figure 6. Is data available to show changes in fat-free mass.
23. Are the pharmacokinetics of BMS-303141 known? Some justification for dose selection should be described. Some indication that the molecule is having on-target activity at the dose used in vivo should be shown.
24. There was no description of the vehicle for many experiments.
25. How were IR-61 and BMS molecules formulated?
26. Standard clinical biochemistry parameters are missing – e.g. ALT, AST, creatinine, BUN.
27. It is not indicated at what time points the GTT and ITT tests were performed.
28. PPAR-r is mentioned in text and figures – do the authors mean PPAR-alpha?
29. T-tests should not be used for individual points on a GTT or ITT curve where repeated measures are performed. Calculation of integrated AUC is standard in the field.


Reviewer #2 (Remarks to the Author):

In the manuscript "Improvement of obesity-associated disorders by a small molecule drug targeting mitochondria of adipose tissue macrophage (ATM)s”, Yawei Wang & co. identified a novel anti-inflammatory small-molecule agent (IR-61) that targets the mitochondrial complexes of ATMs with some degree of specificity. Using in vitro studies in BMDMs and PMs, they demonstrated that IR-61 suppresses macrophage pro-inflammatory activation by potentiating oxidative phosphorylation over glycolysis. Data suggests a mechanistic pathway (ROS/AKT/ACL) by which IR-61 exerts its action.

The data is intriguing but my concerns largely relate to the in vivo phenotyping. They show that there is significant effects on body weight as well as improved inflammation and metabolism. The net summation of the studies suggests that the weight loss is the primary mechanism of the improvement in metabolism and inflammation. The mechanism of the weight loss is not established. This is deemed to be a significant weakness that must be addressed to mechanistically support its utility in obesity and metabolic inflammation.


1. Not sufficiently clear how IR-61 was selected among candidates compounds.
2. Not sufficiently clear how IR-61 enters ATMs to locate preferably in mitochondria.
3. The duration of treatment of obese mice with IR-61 is not noted so we can’t interpret the results. If weight loss is triggered what is the effect on body weight with the acute treatment of the HFD mice? the ND mice? How does this relate to the Figure 6C time course? This is important as it needs to be proven that the effects of IR-61 on deceased inflammation is independent or dependent on the weight loss.
4, It is not clearly apparent that adipocyte size is changed in Fig 5G. The authors should provide direct quantitation of adipocyte cross sectional area distributions to support this qualitative claim.
5. The authors overstate the claim that leukocyte infiltration was “markedly” decreased based on Fig 5G based on two images. Quantification of leukocytes by flow cytometry is the gold standard to make this clain. Or RT-PCR for macrophage or other leukocyte specific markers. Or this statement should be tempered a bit.
6. The mechanism of the weight loss is not provided but needs to be as this is a primary effect of IR-61 that cannot easily be explained by the effect on adipose tissue macrophages alone. While it does not effect food intake what about energy expenditure? Thermogenesis? The methods for measurement of food intake it not provided and the units provided for Fig s12A (food intake/g) does not make sense. This needs to be measured in individually housed mice. CLAMS analysis is required to advance our understanding of the mechanism.
7. The methodology for the GTT is not appropriate as 12h fasting induces significant stress on the animals. In addition, it is unclear if the cause of the improvement in GTT/ITT is due to the weight loss of the effect of IR-61 on inflammation. My suspicion is that this is more linked to the weight loss phenotype which is poorly characteried. The weight loss curve is also concerning. The animals appear to get sick between week 3-7 - why is this?
8.Similar concerns regarding the weight loss dependent or independent effects of IR-61 on steatosis are raised. Weight loss can have the identical effects described.


Minor comments
Apply the same style “pro-“ and “anti-inflammatory“ through the text. Example: Page 2 Line 2 Line 56; Page 4 Line 142; Page 5 Line 205; Page 5 Line 212
Change “treatmnet” in “treatment” Page 3 Line 69
Change “it is not clear that” in “it is not clear if” Page 3 Line 72
Change “our group have” in “our group has” Page 3 Line 89
Remove “Considering the distribution and metabolism of IR-61 in vivo” Page 3 Line 107
Remove “The” from “The results” Page 3 Line 108; Page 9 Line 372; Page 15 Line 604; Page 16 Line 661; Page 17 Line 677; Page 19 Line 698
Remove “the” from “the macrophages” Page 4 Line 157; Page 5 Line 205
Replace “these genes” in “the expression level of these genes“ Page 5 Line 170
Remove “the” from “the plasma of the obese mice…and the lean mice” Page 3 Line 108
Remove “the” from “the mice” Page 9 Line 349, 354
Remove “the” from “the epididymal adipose tissue” Page 9 Line 349, 355
Remove “the” from “the adipose tissue” Page 9 Line 356
Remove “the” from “the SVF” Page 9 Line 358
Remove “the” from “the cells” Page 9 Line 365
Remove “the” from “the basal” Page 9 Line 368
Remove “The” from “The data” Page 10 Line 381
Label axes in Figure 3B
Clarify why IR-61 was selected among other agents.
Clarify mechanism of entry of dye into cells.


Reviewer #3 (Remarks to the Author):

The authors have studied the effects of a compound that accumulatates in mitochondria of macrophages and appears to inhibit inflammation. The authors propse this effect is mediated through increases in ROS that lead to the activation of Akt and ACL. This in turn leads to increases in mitochondrial complex activity and reductions in macrophage inflammation in vitro. In vivo adiminstration of the compound reduces weight gain and lowers inflammation. The study is interesting but there are several important gaps that need to be addressed:
Major Comments:
1. The link between changes in ACL and mitoichondrial respiration/complex respiration is not clear. The atuhros complete studies with a chemical inhibitor of ACL (BMS) but many studies have shown that this compound has many off target effects. In general the mechanism by which ACL alters mitochondrial function is not clear.
2. The invivo studies show dramatic effects on weight loss. Modest weight loss will have a dramatic effect on reducing inflammation so a key question is whether the compound exerts anti-inflamamtory effects independently of this weight loss as proposed given the in vitro work. Therefore, acute experiments establishing anti-inflammatory effects (independently of changes in adiposity) need to be completed.
3. Related to this point above it is entirely unclear how or why the compound is eliciting weight loss. What is the mechanism for this? This needs to be explored. Toxicity or taste aversion could be major factors contributing to the weight loss and must be examined along with potential effects on energy expenditure before there are any changes in body weight.

**Responses to the Comments and Suggestions of Reviewer #1**

1. Mice treated with IR-61 have a very significant change in body mass compared to control animals without a change in food intake. The authors will need to investigate the mechanism for this effect. For example, indirect calorimetry to determine whether IR-61 alters energy expenditure and fecal nutrient content of lipids. Does the molecule induce beige fat or activate brown fat mitochondria?

**Reply:** We thank the reviewer for the comment. As suggested by the reviewer, we monitored whole-body energy expenditure in NCD-fed and HFD-fed mice treated with IR-61 or vehicle control using metabolic cages. Our results showed that IR-61-treated mice exhibited higher oxygen consumption (VO_2_) and increased energy expenditure as compared to treatment with vehicle control (A, Fig. 6, D and F in the revised manuscript). Exhaled carbon dioxide (VCO_2_) was also increased in the IR-61-treated mice (A, Fig. 6E in the revised manuscript). However, IR-61 had no effect on NCD-fed mice (B, fig. s15, A to C in the Supporting Information). In addition, no IR-61-depending difference in fecal lipids was detected in mice fed a HFD (C, fig. s14B in the Supporting Information). Moreover, we found that mRNA levels of the genes associated with WAT (white adipose tissue) browning, including Pgc-1α, Ucp1, Prdm16, Cd137, Tbx1 and Cd40, were induced in IR-61-treated obese mice compared with the vehicle-treated mice (D, fig. s15D in the Supporting Information), but the genes associated with activity of BAT (brown adipose tissue), including Pgc-1α, Ucp1, Cox7a1, Cox8b, Cidea, Tfam and Nrf1, were comparable between two group mice (E, fig. s15E in the Supporting Information). However, IR-61 has no effect on NCD-fed mice (D and E, fig. s15, D and E in the Supporting Information). In summary, IR-61 promoted the development of beige fat, increased energy expenditure and prevented weight gain of HFD-fed mice.

2. Knockout of Acly is toxic and embryonic lethal in mice. The authors show that siRNA knockdown of Acly depletes mitochondrial ETC complexes and diminishes ATP-linked respiration even in the absence of IR-61 treatment. Therefore, the authors do not have sufficient evidence to conclude that Acly is required for IR-61-mediated changes in mitochondrial ETC/activity because the cellular system is significantly impaired by loss of Acly in the first instance. The small molecule BMS drug also wipes out mitochondrial ETC content.

**Reply:** We thank the reviewer for the comment. To determine whether Acly knockdown impaired the viability of macrophages significantly, we conducted Annexin/PI assay using flow cytometry. The results showed that Acly knockdown did not induce apoptosis/necrosis in macrophages (A, fig. s9D in the Supporting Information), which was in consistence with previous studies involving Acly knockdown in normal cells (Cell Metab*.* 2015, 21, 868; Cell Rep. 2017, 21,3003). Therefore, we believe that the change of cell function caused by Acly knockdown was due to its regulatory effect rather than inducing apoptosis. As shown in Fig. 4E and 4F, IR-61 enhanced the expression and activity of mitochondrial complexes, and Acly knockdown not only reduced the expression and activity of mitochondrial complexes but also inhibited the effect of IR-61 on mitochondrial complexes, which indicated that the regulatory function of IR-61 in macrophages depending on Acly. Moreover, BMS-303141 treatment has no toxicity at different concentration in the presence or absence of IR-61 (B, fig. s9E in the Supporting Information).

3. Only one antioxidant (NAC) was tested at one concentration. NAC binds many small molecules. The authors should determine whether NAC directly interacts with IR-61 in a cell-free system. If so, then NAC is simply a sponge that decreases IR-61 availability and this result could markedly change interpretation of the data.

**Reply:** We thank the reviewer for the comment. The method of determining whether NAC interacted with IR-61 was referred to the published article (Pharmacol Rep. 2019, 71, 1067). In brief, 10 μM IR-61 dissolved in PBS alone or together with 20 μM NAC, shaken at 37 °C for 3 h and then LC-MS (Agilent Technologies) was used to detect whether there were new compounds formation. The results of LC-MS showed that no new compounds were detected in the mixture of IR-61 and NAC (A and B). It should be noted that the NAC alone was not detected by LC-MS in this study and previous published article (Pharmacol Rep. 2019, 71, 1067), and the reason may be that NAC could not be charged easily. The absorption spectrum was also applied, and the results showed that there was no difference in the displacement and the strength of absorption peak between pure IR-61 and the mixture of IR-61 and NAC (C). In summary, our results indicated that IR-61 did not interact with NAC in a cell-free system.

4. The authors do not mention how much NAC was used in the cell-based experiments.

**Reply:** We thank the reviewer for the comment. We treated the BMDMs with NAC at 2.5 mM, 5 mM and 10 mM (Fig. 4D in the revised manuscript).

5. Because NAC interacts with a large number of molecules, it is very important to define the minimum amount necessary to block IR-61 effects so that off-target actions can be minimized. For example, NAC treatment alone in Fig 4D blocks Akt and Acly phosphorylation in the absence of IR-61 –

**Reply:** We thank the reviewer for the comment. As suggested, BMDMs were treated with the vehicle control, NAC (2.5 mM, 5 mM and 10 mM), or IR-61 (10 μM) with or without NAC for 6 h and then subjected to immunoblotting. We found that 2.5 mM NAC had a minor effect on phosphorylation of Akt but still blocked the function of IR-61 in macrophages (Fig. 4D in the revised manuscript). In summary, IR-61 promoted Akt and Acly phosphorylation of macrophages via ROS.

6. Histology slides are shown, but there is no indication of whether they were evaluated by a histopathologist.

**Reply:** We thank the reviewer for the comment. As suggested, we invited a histopathologist to evaluate the H&E sections carefully without knowing the sample grouping. The results showed that no abnormal histopathology was observed in NCD-fed mice treated with vehicle or IR-61. HFD could lead to a series of pathological abnormalities including myocardial rupture, liver steatosis and glomerular dilatation. In addition, histology revealed that IR-61 could alleviate liver steatosis in mice fed a HFD (fig. s12 in the Supporting Information). We have added this information in the Supporting Information.

7. The absorption and emission spectra of IR-61 should be shown.

**Reply:** We thank the reviewer for the comment. As suggested, the absorption and emission spectra of IR-61 were shown below.

8. There is no indication of how mitochondrial membrane potential was assessed.

**Reply:** We thank the reviewer for the comment and apologize for the incomplete experiment method display. Mitochondrial membrane potential was determined as reported elsewhere (Cell Metab. 2016, 23, 63). In brief, BMDMs were treated with IR-61 (10 μM) or vehicle control for 24 h, then incubated with 100 nM TMRM (Tetramethylrhodamine, methyl ester) (#T668, Invitrogen) at 37 °C for 30 min and analyzed using flow cytometry. Mitochondrial membrane potential was estimated by measuring the fluorescence intensity of TMRM. We have added the detailed method in the Supporting Information.

9. Can the authors show data to confirm that IR-61 is not interfering with fluorescent dyes DCFH-DA, MitoSOX Red, Mito-Tracker Green, and whatever dye was used to assess membrane potential (methods missing).

**Reply:** We thank the reviewer for the comment. In flow cytometry, the fluorescence channel of IR-61 (Ex 782 nm and Em 805 nm) is APC-CY7, Mito-sox Red (Ex 510 nm and Em 580 nm) and TMRM (Ex 548 nm and Em 574 nm) are PE, and Mito-tracker Green (Ex 488 nm and Em 525 nm) and DCFH-DA (Ex 488 nm and Em 525 nm) are FITC. As shown in Fig. A, the spectrum of APC-CY7 does not coincide with PE and FITC. Moreover, we co-stained the BMDMs with IR-61 and TMRM, Mito-Tracker Green, Mitosox Red or DCFH-DA, respectively, and then analyzed with a FACS system (FACSAria, BD Bioscience). The results showed that the fluorescence channel of IR-61 has no compensation with the channels of TMRM, Mito-Tracker Green, Mitosox Red and DCFH-DA, which indicated that IR-61 had no interference on these fluorescent dyes (B to E, fig. s4, B to E in the Supporting Information).

10. Mitochondria is misspelled in Fig 2G (as mytochondria).

**Reply:** We thank the reviewer for the comment and apologize for our mistake. As suggested, we have corrected the misspelled word.

11. Micromolar uses a capital M while micrometer uses a lowercase m.

**Reply:** We thank the reviewer for the comment and apologize for our mistake. As suggested, we have corrected the misused abbreviation.

12. The authors should justify why 10 micromolar dose of IR-61 was used. Better yet a dose response should be performed to experimentally show that mitoROS production correlates with the biological endpoints.

**Reply:** We thank the reviewer for the comment. In previous experiment we have explored the effect of different concentration of IR-61 on production of mitoROS in macrophages. In brief, BMDMs were treated with various concentration of IR-61 (0, 2.5 μM, 5 μM, 10 μM, 20 μM) for 0.5 h and mitoROS production was detected using flow cytometry. The results showed that the production of mitoROS increased gradually in macrophages with the increase of IR-61 concentration and 10 μM IR-61 make the mitoROS production reached its peak value in BMDMs (fig. s8A, in the Supporting Information).

13. Regarding ATP levels in cells, can the authors confirm that IR-61 is not interfering in the assay? Do ADP and AMP also increase by a similar fold change? Whenever energy charge in a cell changes AMPK activity should be assessed.

**Reply:** We thank the reviewer for the comment. In previous experiments, we found that IR-61 had no effect on ATP levels in BMDMs when treated for a short time (1 h, 3 h and 6 h), indicating that IR-61 had no interference in ATP assay (A, fig. s4A, in the Supporting Information). ADP/ATP ratio was measured using ADP/ATP Ratio Assay Kit (#ab65313, Abcam) and the results showed that the content of ADP and ATP increased in the same fold (B, Fig. 2I in the revised manuscript). Then Ampk activity was determined by assessed phosphorylation level of Ampk and Acc by immunoblotting. The results demonstrated that IR-61 had no effect on Ampk activity (C, fig. s6A in the Supporting Information).

14. Seahorse assay OCR rates are shown in pmol/min, but the correct way to express the data is normalized to cell number or protein content. In this instance the authors treat the cells while they are in the dish over a period of time when cell number and size can change, so the assays should be normalized to protein content.

**Reply:** We thank the reviewer for the comment. As suggested, once the XF experiment was completed, cell proteins were extracted to determine the protein concentration for normalization and the OCR was displayed as pmol/min/μg protein (A and B, Fig. 2, F and G in the revised manuscript; C and D, Fig. 4, G and H in the revised manuscript).

15. The p-Acly site Ser455 is also a PKA substrate so the authors should test an Akt inhibitor and a PKA inhibitor in the presence of IR-61 to determine which kinase is involved in Ser455 phosphorylation

## Reply: We thank the reviewer for the comment. As suggested, BMDMs were treated with LY294002 (AKT inhibitor) or H-89 dihydrochloride (PKA inhibitor) in the presence or absence of IR-61 and then subjected to immunoblotting. The result showed that IR-61 promoted phosphorylation of p-Acly site Ser455 through Akt (A and B, fig. s7, A and B in the Supporting Information).

## 16. Revise gene nomenclature appropriate to species, e.g. when referring to targeting mouse Acly gene with siRNA etc

## Reply: We thank the reviewer for the comment and apologize for our mistakes. As suggested, we have corrected gene nomenclature appropriate to mouse.

17. Spelling error in 2.2 “IR-6 [sic] -treated cells

**Reply:** We thank the reviewer for the comment and apologize for our mistake. As suggested, we have corrected the misspelled word.

18. The content and activity of mitochondrial ETC complexes are increased without a change in mitochondrial mass by MitoTracker staining or transcription factor expression changes. These are minimum supportive data but don’t tell the full story. To better understand the mechanisms linking IR-61 to mitochondrial ETC activity it would be good to show whether mtDNA changes, whether mitochondrial supercomplex formation is altered by using blue native PAGE, whether citrate synthase activity is altered.

**Reply:** We thank the reviewer for the comment. As suggested, we measured macrophage contents of DNAs for mitochondrial gene cytochrome b and for nuclear gene Ppia by qPCR and found no significant difference in mitochondrial DNA after normalizing to nuclear DNA (A, Fig. 3C in the revised manuscript). Moreover, citrate synthase activity of macrophages was not affected by IR-61 (B, Fig. 3D in the revised manuscript). Mitochondrial supercomplex levels in BMDMs were assessed by blue native polyacrylamide gel electrophoresis (BN-PAGE). The results showed that overall contents of supercomplex were increased in BMDMs after IR-61 treatment (C, Fig. 3F in the revised manuscript).

19. Will any molecule with a free sulfhydryl group block IR-61 effects? Do other mitochondrial antioxidants (E.g. SS-31, MnTBAP, mitocatalase) block IR-61 effects?

**Reply:** We thank the reviewer for the comment. According to the above results about IR-61 reacting with NAC, we think that IR-61 could not react with any molecule with a free sulfhydryl group. As suggested, SS-31 and MnTBAP were used to pretreated macrophages. Both in vivo and in vitro experiments showed that the promotion of IR-61 on Akt and Acly phosphorylation was inhibited by these two mitochondrial antioxidants (A to D, fig. s8, B, C, F and G in the Supporting Information).

20. If the effects of IR-61 are solely due to mitoROS signaling as proposed, then does a known control compound such as mito-paraquat have the same effects?

**Reply:** We thank the reviewer for the comment. We agree with the concerns of the reviewer and make a detailed analysis of this issue. The production of mitoROS and its involvement in physiological processes are complicated (Nat Rev Mol Cell Biol. 2020, s41580; Mol Cell. 2012, 48, 158; J Biol Chem. 2017, 292, 6804). For example, different compounds promoting mitoROS generation could trigger different signaling pathways and physiological responses even in same cell models (Autophagy. 2012, 8,559; Chem Biol Interact. 2019, 300, 138). Thus, we speculated that IR-61 and paraquat may have different physiological effects, although they both promote mitoROS production. More importantly, previous study indicated that low dose mito-paraquat augmented mitoROS production without impairing the viability in macrophages. However, paraquat-treated macrophages had increased expression of pro-inflammatory factors (Exp Ther Med. 2019, 17, 1783), which was in contrary to the effect of IR-61 on macrophages.

21. Is siRNA knockdown of Acly toxic – a measure of cell viability should be determined in the absence and presence of IR-61 (as was performed in Fig S6 in WT BMDM cells). A Ser455Ala point mutation would have been a better approach.

**Reply:** We thank the reviewer for the comment. As suggested, we conducted Annexin/PI assay to determine the effect of Acly knockdown on macrophages viability in the presence or absence of IR-61. The results showed that Acly knockdown did not induce apoptosis/necrosis in macrophages (fig. s9D in the Supporting Information), which was in consistence with previous study involving Acly knockdown in normal cells (Cell Metab*.* 2015, 21, 868; Cell Rep. 2017, 21, 3003). Therefore, we think that Acly knockdown by siRNA is an appropriate method to explore Acly function without significantly impairing viability of macrophages.

22. CT data is shown to measure fat mass in Figure 6. Is data available to show changes in fat-free mass

**Reply:** We thank the reviewer for the comment. Analytical microCT (μCT) imaging

can accurately show and compare the fat mass throughout the bodies but not fat-free mass of mice. To clarify the effect of IR-61 on the weight of fat-free mass, mice were anesthetized, executed and then weighed vital organs, including heart, liver, spleen, lung and kidney. The results showed that IR-61 did not lead to the weight change of fat-free organs of NCD-fed mice and IR-61 had no effect on the weight of fat-free organs except for reducing liver weight in obese mice (A to C, fig. s11, A to C in the Supporting Information).

23. Are the pharmacokinetics of BMS-303141 known? Some justification for dose selection should be described. Some indication that the molecule is having on-target activity at the dose used in vivo should be shown.

**Reply:** We thank the reviewer for the comment. Previous study has reported the pharmacokinetics of BMS-3030141 in mice after oral administration (Bioorg Med Chem Lett. 2007, 17, 3208). According to the published research and our experimental purposes, we have explored the appropriated concentration of BMS-303141 in our previous study. We found that the dose (2 mg/kg) we used did not produce toxicity to mice (data not shown) and achieved the expected experimental results. Moreover, adipose tissue macrophages (ATMs) were isolated at different time points after intraperitoneal injection of BMS-303141 and then subjected to immunoblotting. The results showed that BMS-303141 could effectively suppress Acly phosphorylation of ATMs, indicating its on-target activity (fig. s10, in the Supporting Information).

24. There was no description of the vehicle for many experiments.

**Reply:** We thank the reviewer for the comment. The vehicle we used for all experiments was PBS of the same volume as IR-61.

25. How were IR-61 and BMS molecules formulated?

**Reply:** We thank the reviewer for the comment and felt very sorry for the incomplete description in the manuscript. BMS-303141 was purchased from Sigma-Aldrich (#SML0784, Sigma). The synthesis method of IR-61 referred to the article previously reported (Adv. Funct. Mater. 2016, 26, 2826). The method for chemical synthesis of IR-61 was also described in the Supporting Scheme I.

26. Standard clinical biochemistry parameters are missing – e.g. ALT, AST, creatinine, BUN.

**Reply:** We thank the reviewer for the comment. As suggested, serum biochemical parameters for liver/kidney functions were measured. The results showed that obesity increased ALT, AST, BUN, CR and UA levels and IR-61 treatment attenuated the levels of ALT and AST (fig. s13, B and C in the Supporting Information). In addition, IR-61 has no effect on NCD-fed mice (fig. s13, B and C in the Supporting Information).

27. It is not indicated at what time points the GTT and ITT tests were performed.

**Reply:** We thank the reviewer for the comment and apologize for incomplete information display. In Fig. 6G and Fig. s16, mice on a NCD or HFD were treated with IR-61 or vehicle control concurrently for 15 weeks and then GTT and ITT tests were performed. In Fig. 6H, GTT and ITT tests were performed after 6 weeks of IR-61 treatment. In Fig. 6J, obese mice were treated with vehicle control, IR-61, BMS-303141 or BMS-303141 + IR-61 for 2 weeks and then GTT and ITT tests were performed. In Fig. S17 E and F, mice on a NCD or HFD were treated with IR-61 or vehicle control for 2 weeks and then GTT and ITT tests were performed. We have added the information in the revised manuscript and the Supporting Information.

28. PPAR-r is mentioned in text and figures – do the authors mean PPAR-alpha?

**Reply:** We thank the reviewer for the comment and apologize for our wrong writing. PPAR-r meant PPAR-gama and we have corrected it.

29. T-tests should not be used for individual points on a GTT or ITT curve where repeated measures are performed. Calculation of integrated AUC is standard in the field.

**Reply:** We thank the reviewer for the comment and apologize for our mistake. As suggested, the statistical significance of the difference in GTT and ITT between various treatment was determined by two-way analysis of variance (ANOVA) with the Bonferroni correction according to previously published article (Nat Med. 2015, 21, 239). The AUC for each individual animal for GTT and ITT was also analyzed (A, fig. s16 in the Supporting Information; B, Fig. 6G in the revised manuscript; C, Fig. 6H in the revised manuscript; D, fig. s17E in the Supporting Information; E, fig. s17F in the Supporting Information; F, Fig. 6J in the revised manuscript).

**Responses to the Comments and Suggestions of Reviewer #2**

1. Not sufficiently clear how IR-61 was selected among candidate compounds.

**Reply:** We thank the reviewer for the comment. In our recent work, we have identified a class of near-infrared (NIR) heptamethine dyes with preferential accumulation in the mitochondria (Adv Mater. 2017, 29, 1704196; Adv Mater. 2018, e1800475; Gut. 2018, 67,307; Adv. Funct. Mater. 2016, 26, 2826). As the critical role of mitochondrial metabolism in macrophage inflammation (Immunity. 2015, 42, 406

; Eur J Immunol. 2016, 46,13; J Exp Med. 2016, 213, 15; Cell Res. 2015, 25, 771), we desire to develop novel anti-inflammatory drugs basing on the established library of NIR ﬂuorescent dyes. Thus, we focus on macrophage to screen small molecules that can inhibit its pro-inflammatory phenotype. Of these, IR-61 was discovered to be capable of effectively suppressing the expression of pro-inflammatory genes in macrophages and exhibited good biocompatibility. Therefore, we selected IR-61 as a candidate of novel anti-inflammatory agents to obesity treatment and carried out subsequent experiments centering on the regulatory effect of IR-61 on macrophages.

2. Not sufficiently clear how IR-61 enters ATMs to locate preferably in mitochondria.

**Reply:** We thank the reviewer for the comment. IR-61 is a lipophilic cationic NIR heptamethine cyanine dye with the structure-inherent characteristic of mitochondria targeting. Previous studies have indicated that the heptamethine core with lipophilic cationic property is essential for mitochondrial targeting (J Am Chem Soc. 2014, 136, 7018; Adv. Therap. 2018, 1800069; Bioorg Med Chem. 2009, 17, 6631). To determine the specific pathway involved in IR-61 internalization, we pretreated macrophages with amiloride (micropinocytosis inhibitor), chlorpromazine (clathrin-mediated endocytosis inhibitor) and methyl-β-cyclodextrin (MβCD, caveolae-mediated endocytosis inhibitor) and then compared IR-61 uptake in macrophages. The results indicated that IR-61 was internalized via macropinocytosis and clathrin- or caveolae-dependent pathways (A to C, Fig.1 H to J in the revised manuscript). Moreover, high phagocytic capability of macrophages and physicochemical properties of IR-61, including particle size, charge, hydrophilic/lipophilic character and shape, all promote ATMs uptake.

3. The duration of treatment of obese mice with IR-61 is not noted so we can’t interpret the results. If weight loss is triggered what is the effect on body weight with the acute treatment of the HFD mice? the ND mice? How does this relate to the Figure 6C time course? This is important as it needs to be proven that the effects of IR-61 on deceased inflammation is independent or dependent on the weight loss.

**Reply:** We thank the reviewer for the comment. In Figure 6A, the mice were treated with IR-61 or vehicle control at the beginning of HFD or NCD feeding. IR-61 was administered by intraperitoneal injection at 2 mg per kg-1 body weight weekly for 22 weeks. In Figure 6C (Fig. 6B in the revised manuscript), mice were fed a HFD for 12 weeks to induce obesity and then the established obese mice were injected with IR-61 or vehicle control weekly for 10 weeks. As suggested, mice were fed a HFD for 12 weeks to induce obesity, and then we measured expression of pro-inflammatory factors in VAT and liver as well as the levels of pro-inflammatory cytokines in plasma in NCD-fed or HFD-fed mice treated with IR-61 or vehicle control for 2 weeks. The results showed that short term treatment of IR-61 significantly suppressed local and systematic inflammation in obese mice but had no effect on body weight (A to D, fig. s17, A to D in the Supporting Information). Altogether, we demonstrated that the effect of IR-61 on inflammation was independent of weight loss.

4. It is not clearly apparent that adipocyte size is changed in Fig 5G. The authors should provide direct quantitation of adipocyte cross sectional area distributions to support this qualitative claim.

**Reply:** We thank the reviewer for the comment. Adipocyte size was measured according to published protocol (Methods Enzymol. 2014, 537, 93). On HFD, epiWAT of IR-61-treated mice had a greater frequency of small adipocytes and a lower frequency of middle to large adipocytes, resulting in a 30% reduction in adipocytes area. However, IR-61 had no effect on NCD-fed mice (A to C, Fig. 5, H and I in the revised manuscript).

5. The authors overstate the claim that leukocyte infiltration was “markedly” decreased based on Fig 5G based on two images. Quantification of leukocytes by flow cytometry is the gold standard to make this clain. Or RT-PCR for macrophage or other leukocyte specific markers. Or this statement should be tempered a bit.

**Reply:** We thank the reviewer for the comment and apologize for our mistake. As macrophages play the central role in pro-inflammatory environment of VAT (J Clin Invest. 2003, 112, 1796), we measured the expression levels of F4/80, the specific maker of macrophage in VAT using qPCR. The results showed that IR-61 significantly suppressed the expreesion levels of F4/80 in HFD-fed mice but have no effect on NCD-fed mice (Fig.5J in the revised manuscript). Consistent with the decreased number of CLSs containing almost 90% macrophages in VAT, our results indicated that IR-61 could markedly reduce macrophage infiltration of VAT in HFD-fed mice.

6. The mechanism of the weight loss is not provided but needs to be as this is a primary effect of IR-61 that cannot easily be explained by the effect on adipose tissue macrophages alone. While it does not affect food intake what about energy expenditure? Thermogenesis? The methods for measurement of food intake was not provided and the units provided for Fig s12A (food intake/g) does not make sense. This needs to be measured in individually housed mice. CLAMS analysis is required to advance our understanding of the mechanism

**Reply:** We thank the reviewer for the comment. We weighed the food intake of each mouse once a week for 6 consecutive weeks (n=4). As suggested, we performed a series of experiments to make clear the mechanism of IR-61 reducing body weight in obese mice. IR-61 had no effect on food intake and intestinal lipid absorption (A and B, fig. S14, A and B in the Supporting Information). We monitored whole-body energy expenditure in mice using metabolic cages. The results showed that IR-61-treated obese mice exhibited higher oxygen consumption (VO_2_) and increased energy expenditure as compared to treatment with vehicle control (C, Fig. 6, D and F in the revised manuscript). IR-61 treatment also increased exhaled carbon dioxide (VCO_2_) in HFD-fed mice (C, Fig. 6E in the revised manuscript). However, IR-61 had no effect on NCD-fed mice (D, fig. s15, A to C in the Supporting Information). Since infiltration of pro-inflammatory M1 macrophages inhibits beige adipogenesis in obesity (J Intern Med. 2018, 284, 492; Am J Physiol Endocrinol Metab. 2016, 310, E676; Nat Immunol. 2017, 18, 654), we asked the effect of IR-61 on browning/beiging of WAT or BAT activity. The results showed that mRNA levels of the genes associated with WAT browning were induced in IR-61-treated obese mice compared with the vehicle-treated mice (E, fig. s15D in the Supporting Information), but the genes associated with activity of BAT were comparable between two group mice (F, fig. s15E in the Supporting Information). Altogether, we demonstrated that IR-61 reversed the inhibition of inflammation on beige adipogenesis, increased energy expenditure and prevented weight gain of HFD-fed mice

7. The methodology for the GTT is not appropriate as 12h fasting induces significant stress on the animals. In addition, it is unclear if the cause of the improvement in GTT/ITT is due to the weight loss of the effect of IR-61 on inflammation. My suspicion is that this is more linked to the weight loss phenotype which is poorly characteried. The weight loss curve is also concerning. The animals appear to get sick between week 3-7 - why is this?

**Reply:** We thank the reviewer for the comment. In this study, GTT was performed according to the previous published article (Cell Rep. 2014, 7, 223) and we did not find obvious stress in this condition. Moreover, in many published articles involving GTT, either 4-6 h fasting or overnight fasting was employed (Nat Commun. 2017, 8, 1322; Cell. 2014, 158, 25; J Clin Invest. 2017,127, 3152). Chronic tissue inflammation is directly associated with insulin resistance (J Clin Invest. 2017, 127, 1). To illuminate whether the improved insulin sensitivity observed in IR-61-treated obese mice was linked to the direct anti-inflammatory effect of IR-61, or the reduction of inflammation caused by weight loss, we conducted GTT/ITT prior to body weight change. The results showed that IR-61 improved insulin sensitivity as well as alleviated local and systemic inflammation prior to weight change (A, fig. s17A in the Supporting Information; B and C, fig. s17, E and F in the Supporting Information; D to F, fig. s17, B to D in the Supporting Information). Thus, we believe that the benefit effect of IR-61 on insulin sensitivity was directly mediated by its anti-inflammatory effect rather than depending on weight loss. The body weight of mice is very sensitive to the external environment, including light, temperature and circadian rhythm, etc. In the process of mice feeding, some accidental environmental changes will affect the weight of mice and similar weight curve is often seen in other experiments involving HFD-induced obese mice (Nat Med. 2013, 19, 313; EMBO Mol Med. 2018, 10: e8791; Nat Med. 2016, 22, 1023). Moreover, in the whole process of mice feeding, the body weight of mice fed a NCD or HFD is gradually increasing.

8. Similar concerns regarding the weight loss dependent or independent effects of IR-61 on steatosis are raised. Weight loss can have the identical effects described.

**Reply:** We thank the reviewer for the comment. In this study, we have demonstrated that the effect of IR-61 on inflammation was independent of weight change (fig. s17 in the Supporting Information) and IR-61 prevented weight gain by reversing the inhibition of inflammation on beige adipogenesis, increased energy expenditure in HFD-fed mice (fig. s15D in the Supporting Information; Fig. 6, D to F in the revised manuscript). Adipose tissues link tightly with hepatic steatosis (Gut. 2018, 67, 1317; J Hepatol. 2019, 71, 1012; J Hepatol. 2009, 51, 354). In insulin-resistant state, VAT is the primary source of serum free fatty acids (FFA) used for hepatic fat synthesis and ATMs directly promote hepatic inflammation. Considering that IR-61 could reduce VAT and liver inflammation as well as improve insulin sensitivity prior to weight change (fig. s17 in the Supporting Information), we conclude that IR-61 treatment was the primary cause of steatosis improvement in obese mice.

**Responses to the Comments and Suggestions of Reviewer #3**

1. The link between changes in ACL and mitoichondrial respiration/complex respiration is not clear. The authors complete studies with a chemical inhibitor of ACL (BMS) but many studies have shown that this compound has many off target effects. In general, the mechanism by which ACL alters mitochondrial function is not clear.

**Reply:** We thank the reviewer for the comment. It has been proven that Acly increased content and activity of mitochondrial complex by increasing cardiolipin content (Cell Metab. 2015, 21, 868). Cardiolipin is critical in the mitochondrial complex stabilization and mitochondrial supercomplex organization (Cell Metab. 2010, 12, 154; Biochim Biophys Acta. 2014, 408; Prog Lipid Res. 2014, 55, 1). In order to clarify the relationship between Acly and mitochondrial complex in BMDMs, we measured mitochondrial cardiolipin content of IR-61-treated macrophages after transfected with Acly siRNA. The results demonstrated that IR-61 increased cardiolipin content depending on Acly in macrophages (A, fig. s9F in the Supporting Information). BMS-303141 is a Acly inhibitor that widely used in Acly-related experiments (Nat Commun. 2019 ,10, 2042; Circulation. 2019, 139, 119; Nat Commun. 2016, 7, 11320). To exclude off target effects of BMS-303141, we detected Acly activity of macrophages after BMS-303141 treatment in vivo. The results indicated that BMS-303141 could suppress Acly phosphorylation effectively (B, fig. s10 in the Supporting Information).

2. The invivo studies show dramatic effects on weight loss. Modest weight loss will have a dramatic effect on reducing inflammation so a key question is whether the compound exerts anti-inflamamtory effects independently of this weight loss as proposed given the in vitro work. Therefore, acute experiments establishing anti-inflammatory effects (independently of changes in adiposity) need to be completed.

**Reply:** We thank the reviewer for the comment. As suggested, mice were fed a HFD for 12 weeks to induce obesity, and then we measured expression of pro-inflammatory factors in VAT, liver as well as the levels of pro-inflammatory cytokines in plasma in NCD-fed or HFD-fed mice treated with IR-61 or vehicle control for 2 weeks. The results showed that short term treatment of IR-61 significantly suppressed local and systematic inflammation in obese mice but had no effect on body weight (A, fig. s17A in the Supporting Information; B to D, fig. s17, B to D in the Supporting Information). Thus, we believe that the effect of IR-61 on inflammation was independent of weight change.

3. Related to this point above it is entirely unclear how or why the compound is eliciting weight loss. What is the mechanism for this? This needs to be explored. Toxicity or taste aversion could be major factors contributing to the weight loss and must be examined along with potential effects on energy expenditure before there are any changes in body weight.

**Reply:** We thank the reviewer for the comment. As suggested, we performed a series of experiments to make clear the mechanism of IR-61 reducing body weight in obese mice. IR-61 had no effect on food intake and intestinal lipid absorption (Fig1A and 1B, fig. S14, A and B in the Supporting Information). We monitored whole-body energy expenditure in mice using metabolic cages. We found that IR-61-treated obese mice exhibited higher oxygen consumption (VO_2_) and increased energy expenditure as compared to treatment with vehicle control (Fig1C, Fig. 6, D and F in the revised manuscript). IR-61 treatment also increased exhaled carbon dioxide (VCO_2_) in HFD-fed mice (Fig1C, Fig. 6E in the revised manuscript). However, IR-61 had no effect on NCD-fed mice (Fig1D, fig. s15, A to C in the Supporting Information). Since infiltration of pro-inflammatory M1 macrophages inhibits beige adipogenesis in obesity (J Intern Med. 2018, 284, 492; Am J Physiol Endocrinol Metab. 2016, 310, E676; Nat Immunol. 2017, 18, 654), we asked the effect of IR-61 on browning/beiging of WAT or BAT activity. The results showed that mRNA levels of the genes associated with WAT browning were induced in IR-61-treated obese mice compared with the vehicle-treated mice (Fig1E, fig. s15D in the Supporting Information), but the genes associated with activity of BAT were comparable between two group mice (Fig1F, fig. s15E in the Supporting Information). H&E staining (Fig2, fig. s12 in the Supporting Information), blood routine (Fig3A, fig. s13A in the Supporting Information) and biochemical measures (Fig3B and 3C, fig. s13, B and C in the Supporting Information) revealed that IR-61 not only had no toxicity to mice, but also reduced the liver and kidney damage induced by obesity . In summary, IR-61 prevented weight gain of HFD-fed mice through increasing energy expenditure rather than toxicity or food intake.

Reviewer #1 (Remarks to the Author):

The authors have certainly performed a number of additional studies that have improved the manuscript. The mechanism linking IR-61 to its anti-obesity effects is emerging, but still not completely clear. It would help to better understand the acute vs chronic effects of IR-61 to determine whether it has anti-obesity effects directly or indirectly (e.g. via beige fat). There are several other points that require clarification as follows:

1. Mice treated with IR-61 have a very significant change in body mass compared to control animals without a change in food intake. The authors will need to investigate the mechanism for this effect. For example, indirect calorimetry to determine whether IR-61 alters energy expenditure and fecal nutrient content of lipids. Does the molecule induce beige fat or activate brown fat mitochondria?
Reply: We thank the reviewer for the comment. As suggested by the reviewer, we monitored whole-body energy expenditure in NCD-fed and HFD-fed mice treated with IR-61 or vehicle control using metabolic cages. Our results showed that IR-61-treated mice exhibited higher oxygen consumption (VO2) and increased energy expenditure as compared to treatment with vehicle control (A, Fig. 6, D and F in the revised manuscript). Exhaled carbon dioxide (VCO2) was also increased in the IR-61-treated mice (A, Fig. 6E in the revised manuscript). However, IR-61 had no effect on NCD-fed mice (B, fig. s15, A to C in the Supporting Information). In addition, no IR-61-depending difference in fecal lipids was detected in mice fed a HFD (C, fig. s14B in the Supporting Information). Moreover, we found that mRNA levels of the genes associated with WAT (white adipose tissue) browning, including Pgc-1α, Ucp1, Prdm16, Cd137, Tbx1 and Cd40, were induced in IR-61-treated obese mice compared with the vehicle-treated mice (D, fig. s15D in the Supporting Information), but the genes associated with activity of BAT (brown adipose tissue), including Pgc-1α, Ucp1, Cox7a1, Cox8b, Cidea, Tfam and Nrf1, were comparable between two group mice (E, fig. s15E in the Supporting Information). However, IR-61 has no effect on NCD-fed mice (D and E, fig. s15, D and E in the Supporting Information). In summary, IR-61 promoted the development of beige fat, increased energy expenditure and prevented weight gain of HFD-fed mice.

Reviewer reply: It is good to see additional data that addresses the major concern; however, the authors still need to address the following:
1a. It is not possible to interpret the indirect calorimetry data shown. The methods are incomplete because the normalization method is not described. I am forced to *assume* that the data are normalized to body mass; however, body mass is not appropriate. For example, two sets of mice with the same VO2 consumption would show that the fat mouse has less VO2 simply because you are dividing VO2 by a larger number, while the real oxygen consumption rate isn’t altered between mice. When comparing sets of mice with different adiposity, such as the case here, the acceptable method of normalization involves ANCOVA (see doi.org/10.3389/fphys.2013.00034).
1b. Locomotor activity data is missing. It is important to know if hyperactivity is related to changes in oxygen consumption.
1c. Fig 6d-f legend needs to say how long mice had been treated with IR-61.
1d. the word treated is misspelled
1e. flow rates of air and cage sizes need to be included in methods.
1f. Testing how IR-61 affects indirect calorimetry on day 1 of treatment is important for understanding whether the compound has direct effects on energy expenditure or indirect effects potentially through beige fat. By treating mice of identical body composition, you can also avoid some of the complications described above in 1a. The experiment should be done in obese mice considering that the molecule doesn’t work in lean animals.
1g. Protein expression of UCP1 in subcutaneous white fat should be assessed as beige fat is emerging as a potential anti-obesity mechanism.


2. Knockout of Acly is toxic and embryonic lethal in mice. The authors show that siRNA knockdown of Acly depletes mitochondrial ETC complexes and diminishes ATP-linked respiration even in the absence of IR-61 treatment. Therefore, the authors do not have sufficient evidence to conclude that Acly is required for IR-61-mediated changes in mitochondrial ETC/activity because the cellular system is significantly impaired by loss of Acly in the first instance. The small molecule BMS drug also wipes out mitochondrial ETC content.
Reply: As shown in Fig. 4E and 4F, IR-61 enhanced the expression and activity of mitochondrial complexes, and Acly knockdown not only reduced the expression and activity of mitochondrial complexes but also inhibited the effect of IR-61 on mitochondrial complexes, which indicated that the regulatory function of IR-61 in macrophages depending on Acly.

Reviewer reply: I disagree that the data proves Acly is directly required for the function of IR-61. Acly has a major deleterious effect on mito complexes to begin with - the fact that IR-61 doesn’t restore mito complexes when Acly is inhibited does not mean that Acly is required for the IR-61 mechanism. Consider the hypothetical scenario that Acyl inhibition irreversibly blocks expression of a transcription factor required for expression of mito complexes – if IR-61 works via that transcription factor to increase mito complexes then IR-61 would not be able to increase mito complexes. This hypothetical scenario links IR-61 directly to the transcription factor in a mechanism that is normally 100% independent of Acly. Acly inhibition could just indirectly block IR-61’s mechanism without being involved in the mechanism of action directly per se, in a similar scenario that Tfam inhibition could, or Pgc1a inhibition could, or Polrmt could etc etc.

5. Because NAC interacts with a large number of molecules, it is very important to define the minimum amount necessary to block IR-61 effects so that off-target actions can be minimized. For example, NAC treatment alone in Fig 4D blocks Akt and Acly phosphorylation in the absence of IR-61 –
Reply: We thank the reviewer for the comment. As suggested, BMDMs were treated with the vehicle control, NAC (2.5 mM, 5 mM and 10 mM), or IR-61 (10 μM) with or without NAC for 6 h and then subjected to immunoblotting. We found that 2.5 mM NAC had a minor effect on phosphorylation of Akt but still blocked the function of IR-61 in macrophages (Fig. 4D in the revised manuscript). In summary, IR-61 promoted Akt and Acly phosphorylation of macrophages via ROS.

Reviewer reply: The lowest NAC dose still blocks Acly and Akt phosphorylation on its own, so it complicates interpretation of the data that IR-61 requires ROS to increase Akt and Acly phosphorylation. However, the new data in comment 19 add more scientific rigor to support this mechanism.

22. CT data is shown to measure fat mass in Figure 6. Is data available to show changes in fat-free mass
Reply: We thank the reviewer for the comment. Analytical microCT (μCT) imaging
can accurately show and compare the fat mass throughout the bodies but not fat-free mass of mice. To clarify the effect of IR-61 on the weight of fat-free mass, mice were anesthetized, executed and then weighed vital organs, including heart, liver, spleen, lung and kidney. The results showed that IR-61 did not lead to the weight change of fat-free organs of NCD-fed mice and IR-61 had no effect on the weight of fat-free organs except for reducing liver weight in obese mice (A to C, fig. s11, A to C in the Supporting Information).
Reviewer Response: I should have been more specific, but loss of lean mass as skeletal muscle was the point of my question. No need to kill more mice if the data in muscle are not available.

25. How were IR-61 and BMS molecules formulated?
Reply: We thank the reviewer for the comment and felt very are sorry for our theunclear incomplete description in administration approachthe manuscript. BMS-303141 was purchased from Sigma-Aldrich (#SML0784, Sigma). The synthesis method of IR-61 referred to the article previously reported (Adv. Funct. Mater. 2016, 26, 2826). The method for chemical synthesis of IR-61 was also described in the Supporting Scheme I.

Reviewer Response: Please confirm if they were delivered in saline.


Reviewer #2 (Remarks to the Author):

Thank you for providing additional data. My concerns have been addressed


Reviewer #3 (Remarks to the Author):

The authors have attempted to address my 3 major comments but this has led to further questions on 2 of the points described below:
1. With regard to target engagement and off target effects the authors have shown reductions in 454 phosphorylation and changes cardiolipin. However, it is not clear to me why the BMS compound would inhibit 454 phosphorylation unless it was inhibiting Akt and many possible pathways could contribute to the reduction in cardiolipin. I would suggest a simple experiment where lipogenesis is measured in control and ACLY siRNA macrophages +/- BMS/IR would be most beneficial to support the conclusions.
2. The authors have done a good job showing that the anti-inflammatory effects of IR are independent of obesity. I would suggest these data be included in the main manuscript rather than the supplement as they are central to the conclusions of the paper.
3. The data in Figure 6 are not sufficient to show the cause of the weight loss since the mice being tested are already much lighter and hence when correcting for body mass they appear to have increase EE. The much lower body weight would also be predicted to cause greater browning. Therefore, it is imperative that the authors measure metabolic rate and adipose beiging in animals before the difference in body weight occurs. An ideal experiment would be after 2 weeks as completed in point 2 above when there were anti-inflammatory effects but no weight significant weight loss.

**Responses to the Comments and Suggestions of Reviewer #1**

1. It is good to see additional data that addresses the major concern; however, the authors still need to address the following:
1a. It is not possible to interpret the indirect calorimetry data shown. The methods are incomplete because the normalization method is not described. I am forced to *assume* that the data are normalized to body mass; however, body mass is not appropriate. For example, two sets of mice with the same VO2 consumption would show that the fat mouse has less VO2 simply because you are dividing VO2 by a larger number, while the real oxygen consumption rate isn’t altered between mice. When comparing sets of mice with different adiposity, such as the case here, the acceptable method of normalization involves ANCOVA (see doi.org/10.3389/fphys.2013.00034).

**Reply:** We thank the reviewer for the comment and apologize for incomplete information display. In previous study, we normalized the metabolic rate by dividing VO_2_, VCO_2_ and heat production by total body mass and expressed as ml/kg/h or kcal/kg/h, which was referred to previously published articles (Nat Commun. 2019, 10, 3536; Nat Med. 2015, 21, 159). As suggested, ANCOVA was used to analyze the data in Fig. 6 D to F with body mass as a covariate (Front Physiol. 2013, 4, 34). ANCOVA must satisfy the assumptions of parallel regression lines and linear relationships between covariates and dependent variables (Int J Obes (Lond). 2006, 30, 1322). We found the significant difference in VO_2_ and VCO_2_ during daytime between IR-61 and control groups in the condition that these data were suitable for ANCOVA (p<0.05, Fig. B and D), which was consistent with the results with normalization of body mass. However, the data of VO_2_ and VCO_2_ during night did not satisfy the assumption that the regression lines of each group were parallel (p<0.05, Fig. A and C). Moreover, the relationships between body mass and heat production were non-linear, even when log_10_-transformation was used (p>0.05, Fig. E to H). The reason for the failure of ANCOVA to work effectively may be that for mice individuals, many factors contributed to the variation in metabolic rates, leading to some metabolic values were outliers (J Clin Invest. 1986, 78, 1568). Moreover, like many mice experiments, low test power caused by limited sample size also led to the non-linear relationship (Diabetes. 2010, 59, 323). Although normalization of body mass exists defects, it still has the ability to find differences (Int J Obes (Lond). 2006, 30, 1322) and is widely applied in numerous similar studies (Nat Commun. 2019, 10, 3536; Nat Med. 2015, 21, 159; Nat Metab. 2019, 1, 340; Nat Metab. 2019, 1, 58; Cell Metab. 2016, 24, 510). Altogether, we agree with that ANCOVA is a powerful method for analyzing these types of data, but the method normalized by diving body weight seems to be more practical in our study.

1b. Locomotor activity data is missing. It is important to know if hyperactivity is related to changes in oxygen consumption.
**Reply:** We thank the reviewer for the comment and apologize for incomplete information display. As suggested, we counted the total activity of mice (fig. s15, D and E in the Supporting Information) and found no significant difference in total activity between IR-61-treated and vehicle-treated mice.

1c. Fig 6d-f legend needs to say how long mice had been treated with IR-61.

**Reply:** We thank the reviewer for the comment and apologize for incomplete information display. Mice were treated with IR-61 or vehicle for 2 weeks, and then whole-body energy expenditure was measured before body weight change. We have added the information in the legend of Fig. 6D-F in the revised manuscript.

1d. the word treated is misspelled

**Reply:** We thank the reviewer for the comment and apologize for our mistake. As suggested, we have corrected the misspelled word.

1e. flow rates of air and cage sizes need to be included in methods.
**Reply:** We thank the reviewer for the comment and apologize for incomplete information display. Flow rates of air is 0.5 L/min and cage size is 320*215*170 mm. We have added the information in methods in the revised manuscript.

1f. Testing how IR-61 affects indirect calorimetry on day 1 of treatment is important for understanding whether the compound has direct effects on energy expenditure or indirect effects potentially through beige fat. By treating mice of identical body composition, you can also avoid some of the complications described above in 1a. The experiment should be done in obese mice considering that the molecule doesn’t work in lean animals.
**Reply:** We thank the reviewer for the comment. As suggested, obesity mice with comparable weight were treated with IR-61 or vehicle control for 1 day and then whole-body energy expenditure and activity were monitored. We found that IR-61 had no effect on VO_2_, VCO_2_, heat production and activity in HFD-fed mice (fig. s16, A to D in the Supporting Information), which indicated that IR-61 could not increase energy expenditure directly.

1g. Protein expression of UCP1 in subcutaneous white fat should be assessed as beige fat is emerging as a potential anti-obesity mechanism.
**Reply:** We thank the reviewer for the comment. As suggested, proteins extracted from inguinal white adipose tissue (iWAT) of NCD-fed or HFD-fed mice treated with IR-61 or vehicle control for 2 weeks were subjected to western blot. We found that IR-61 promoted Ucp1 expression of iWAT in obese mice but had no effect on lean mice (fig. s16E in the Supporting Information).

2. I disagree that the data proves Acly is directly required for the function of IR-61. Acly has a major deleterious effect on mito complexes to begin with - the fact that IR-61 doesn’t restore mito complexes when Acly is inhibited does not mean that Acly is required for the IR-61 mechanism. Consider the hypothetical scenario that Acyl inhibition irreversibly blocks expression of a transcription factor required for expression of mito complexes – if IR-61 works via that transcription factor to increase mito complexes then IR-61 would not be able to increase mito complexes. This hypothetical scenario links IR-61 directly to the transcription factor in a mechanism that is normally 100% independent of Acly. Acly inhibition could just indirectly block IR-61’s mechanism without being involved in the mechanism of action directly per se, in a similar scenario that Tfam inhibition could, or Pgc1a inhibition could, or Polrmt could etc.

**Reply:** We thank the reviewer for the comment. We agree with the concept of the reviewer and carry on thorough thinking about this issue. As pointed out by the reviewer, the current data could not fully demonstrate that IR-61 regulated mitochondrial function directly through Acly. As shown in Fig. 4, A and D, fig. s7 and fig. s8, IR-61 promoted phosphorylation of Acly through ROS-Akt pathway. Moreover, Acly linked tightly with the content and activity of mitochondrial complexes as shown in this study and previous reported study (Cell Metab*.* 2015, 21, 868). Therefore, although we did not have enough evidence to prove that Acly was the direct requirement for the IR-61 function, maybe it was more reasonable to regard Acly as an indirect component of IR-61 in regulating mitochondrial function. Moreover, in order to avoid misunderstanding, we have made a more accurate explanation about the relationship between IR-61 and Acly in the current manuscript.

3. The lowest NAC dose still blocks Acly and Akt phosphorylation on its own, so it complicates interpretation of the data that IR-61 requires ROS to increase Akt and Acly phosphorylation. However, the new data in comment 19 add more scientific rigor to support this mechanism.

**Reply:** We thank the reviewer for the comment. We detected mitoROS levels in BMDMs treated with 2.5 μM NAC or vehicle control for 2 h using flow cytometry. The results showed that 2.5 μM NAC could suppress mitoROS effectively, indicating the critical role of ROS in IR-61 promoting Akt and Acly phosphorylation.

4. I should have been more specific, but loss of lean mass as skeletal muscle was the point of my question. No need to kill more mice if the data in muscle are not available.

**Reply:** We thank the reviewer for the comment and apologize for not giving full consideration to this issue. When weighing the fat-free organs, we did not collect the data about skeletal muscle weight.

5. Please confirm if they were delivered in saline.

**Reply:** We thank the reviewer for the comment. BMS-303141 and IR-61 were first dissolved into stock solution with DMSO, and then diluted to the requirement concentration in PBS.

**Reviewer #2 (Remarks to the Author):**

Thank you for providing additional data. My concerns have been addressed.

**Responses to the Comments and Suggestions of Reviewer #3**

1. With regard to target engagement and off target effects the authors have shown reductions in 454 phosphorylation and changes cardiolipin. However, it is not clear to me why the BMS compound would inhibit 454 phosphorylation unless it was inhibiting Akt and many possible pathways could contribute to the reduction in cardiolipin. I would suggest a simple experiment where lipogenesis is measured in control and ACLY siRNA macrophages +/- BMS/IR would be most beneficial to support the conclusions.

**Reply:** We thank the reviewer for the comment. The activity of Acly was highly dependent on its phosphorylation at S^454^ and its phosphorylation level was positively correlated with enzyme activity (Cell Metab*.* 2015, 21, 868; Biochemistry 2000, 39, 1169; J Biol Chem. 2002, 277, 33895). Other commercial Acly inhibitors, like HCA and SB-204990, also suppressed S^454^ phosphorylation of Acly, reflecting the inhibition of its enzyme activity (Clin Cancer Res. 2020, 26, 2725). BMS-303141 is a commercial Acly inhibitor purchased from Sigma-Aldrich (#SML0784, Sigma) and is widely used in Acly-inhibiting experiments (Nat Commun. 2019 ,10, 2042; Circulation. 2019, 119; Nat Commun. 2016, 7, 11320), but its mechanism is still unclear. As suggested, we measured mitochondrial cardiolipin content of control and Acly knockdown BMDMs treated with or without BMS-303141/IR-61. The results demonstrated that BMS-303141 reduced cardiolipin content depending on Acly in macrophages (fig. s9F in the Supporting Information).

2. The authors have done a good job showing that the anti-inflammatory effects of IR are independent of obesity. I would suggest these data be included in the main manuscript rather than the supplement as they are central to the conclusions of the paper.

**Reply:** We thank the reviewer for the comment. As suggested, we have added these data in the revised main manuscript.

3. The data in Figure 6 are not sufficient to show the cause of the weight loss since the mice being tested are already much lighter and hence when correcting for body mass they appear to have increase EE. The much lower body weight would also be predicted to cause greater browning. Therefore, it is imperative that the authors measure metabolic rate and adipose beiging in animals before the difference in body weight occurs. An ideal experiment would be after 2 weeks as completed in point 2 above when there were anti-inflammatory effects but no weight significant weight loss.

**Reply:** We thank the reviewer for the comment and apologize for incomplete information display. As with the protocol used to explore inflammation, we measured the metabolic rate and adipose beiging of mice with comparable weight after 2 weeks of IR-61 or vehicle control treatment. However, due to our carelessness, we did not add this important information in the response to the reviewer’s comments and only displayed it in the previous manuscript (line 273-274 in the previous manuscript, line 274-275 in the current manuscript). We have added this information in the legend of Fig. 6D-F in the current revised manuscript and fig. s15A-E and fig. s16E-G in the Supporting Information

Reviewer #1 (Remarks to the Author):

The authors have made substantial contributions to improve the manuscript. I have only 1 remaining concern to the first point (my comment is in the final paragraph).

1. It is good to see additional data that addresses the major concern; however, the authors still need to address the following:
1a. It is not possible to interpret the indirect calorimetry data shown. The methods are incomplete because the normalization method is not described. I am forced to *assume* that the data are normalized to body mass; however, body mass is not appropriate. For example, two sets of mice with the same VO2 consumption would show that the fat mouse has less VO2 simply because you are dividing VO2 by a larger number, while the real oxygen consumption rate isn’t altered between mice. When comparing sets of mice with different adiposity, such as the case here, the acceptable method of normalization involves ANCOVA (see doi.org/10.3389/fphys.2013.00034).
Reply: We thank the reviewer for the comment and apologize for incomplete information display. In previous study, we normalized the metabolic rate by dividing VO2, VCO2 and heat production by total body mass and expressed as ml/kg/h or kcal/kg/h, which was referred to previously published articles (Nat Commun. 2019, 10, 3536; Nat Med. 2015, 21, 159). As suggested, ANCOVA was used to analyze the data in Fig. 6 D to F with body mass as a covariate (Front Physiol. 2013, 4, 34). ANCOVA must satisfy the assumptions of parallel regression lines and linear relationships between covariates and dependent variables (Int J Obes (Lond). 2006, 30, 1322). We found the significant difference in VO2 and VCO2 during daytime between IR-61 and control groups in the condition that these data were suitable for ANCOVA (p<0.05, Fig. B and D), which was consistent with the results with normalization of body mass. However, the data of VO2 and VCO2 during night did not satisfy the assumption that the regression lines of each group were parallel (p<0.05, Fig. A and C). Moreover, the relationships between body mass and heat production were non-linear, even when log10-transformation was used (p>0.05, Fig. E to H). The reason for the failure of ANCOVA to work effectively may be that for mice individuals, many factors contributed to the variation in metabolic rates, leading to some metabolic values were outliers (J Clin Invest. 1986, 78, 1568). Moreover, like many mice experiments, low test power caused by limited sample size also led to the non-linear relationship (Diabetes. 2010, 59, 323). Although normalization of body mass exists defects, it still has the ability to find differences (Int J Obes (Lond). 2006, 30, 1322) and is widely applied in numerous similar studies (Nat Commun. 2019, 10, 3536; Nat Med. 2015, 21, 159; Nat Metab. 2019, 1, 340; Nat Metab. 2019, 1, 58; Cell Metab. 2016, 24, 510). Altogether, we agree with that ANCOVA is a powerful method for analyzing these types of data, but the method normalized by diving body weight seems to be more practical in our study.

Reviewer Response: It is misleading to claim that the drug-treated animals have increased energy expenditure if the major factor influencing the VO2 reading is body fat difference between groups of mice (Nat Methods. 2011 Dec 28; 9(1): 57–63). It is also not an appropriate rebuttal to cite that other people normalize to body weight so you should be able to as well. If ANCOVA is not possible because of too few n’s, then the next best approach is to normalize by lean mass + 0.2% fat mass. If that is not possible because the authors do not have lean mass data, then the next best approach is to show mL/hr/mouse and assume that the mice have the same lean body mass. If there is still increased VO2 in the drug-treated group, then the increase in energy expenditure is real. You might consider expressing the data both ways, mL/hr/mouse and mL/hr/kg body weight, so that readers have all of the data to make their own informed decision. It also remains possible that there might be an increase in VO2 that is too hard to detect due to the noise in this assay. A more rigorous discussion of this data is required.


Reviewer #3 (Remarks to the Author):

No further comments.

**Responses to the Comments and Suggestions of Reviewer #1**

Reviewer Response: It is misleading to claim that the drug-treated animals have increased energy expenditure if the major factor influencing the VO2 reading is body fat difference between groups of mice (Nat Methods. 2011 Dec 28; 9(1): 57–63). It is also not an appropriate rebuttal to cite that other people normalize to body weight so you should be able to as well. If ANCOVA is not possible because of too few n’s, then the next best approach is to normalize by lean mass + 0.2% fat mass. If that is not possible because the authors do not have lean mass data, then the next best approach is to show mL/hr/mouse and assume that the mice have the same lean body mass. If there is still increased VO2 in the drug-treated group, then the increase in energy expenditure is real. You might consider expressing the data both ways, mL/hr/mouse and mL/hr/kg body weight, so that readers have all of the data to make their own informed decision. It also remains possible that there might be an increase in VO2 that is too hard to detect due to the noise in this assay. A more rigorous discussion of this data is required.

**Reply:** We thank the reviewer for the comment. To clarify the effect of IR-61 on energy expenditure in HFD-fed mice clearly, we redid this experiment with a double sample size (n=11). We found that our data satisfied the assumptions of parallel regression lines and linear relationships between covariates and dependent variables when using ANCOVA (Fig A to C). Moreover, IR-61 increased VO_2_, VCO_2_ and heat production in HFD-fed mice after normalization of body mass or adjustment for the covariate body mass using regression-based analysis of covariance (ANCOVA) (Fig. 6, D to F in the revised manuscript). The analysis method was referred to previously published articles (Front Physiol. 2013, 4, 34; Cell Res. 2017, 27, 1309; Mol Metab. 2019, 22, 62) and we have added it in the revised manuscript.

**Reviewer #3 (Remarks to the Author):**

No further comments.

Reviewer #1 (Remarks to the Author):

The respirometry data in Fig 6D is stated to be from mice fed HFD and treated with IR-61 for 2 weeks. However, it still remains unclear in the text whether the 2 week treatment respirometry data is from the protocol in Fig 6A (administered at the start of HFD) or Fig 6B (administered in previously obese mice). The difference is important because after 2 weeks of treatment the mice in 6A have no change in body composition, while in 6B the treated mice are about 10-15% lighter.

My concern with the data in question is that if there is a difference in adiposity then normalizing oxygen consumption (or heat) to total body mass is potentially misleading because adipose tissue has a fraction of the metabolic rate of other lean mass tissues. This is why I requested to see the data shown without normalization.

The authors have repeated the experiment in question, but unfortunately did not collect lean and fat mass data for normalization. It would be better to see the data without normalization (i.e. VO2 per mouse), than normalized to body weight. The authors have the data so there's no more experiments to perform. Simply create a new supplemental figure of the respirometry data without dividing by body mass. Even if the data shows that the energy expenditure phenotype disappears that is ok, the data is what it is, it leaves the door open to future investigations. The metabolic physiologist readers can form their own conclusions only if they can see the data without normalization to body mass.

Here is a very recent example of indirect calorimetry data from an anti-obesity study with respirometry data normalized per mouse: Bernier et al., 2020, Cell Metabolism 32, 203–214. The mechanism of weight loss in this paper isn't necessarily clear, which is ok, but at least the reader can see the data for what it is.

**Responses to the Comments and Suggestions of Reviewer #1**

The respirometry data in Fig 6D is stated to be from mice fed HFD and treated with IR-61 for 2 weeks. However, it still remains unclear in the text whether the 2 week treatment respirometry data is from the protocol in Fig 6A (administered at the start of HFD) or Fig 6B (administered in previously obese mice). The difference is important because after 2 weeks of treatment the mice in 6A have no change in body composition, while in 6B the treated mice are about 10-15% lighter.
My concern with the data in question is that if there is a difference in adiposity then normalizing oxygen consumption (or heat) to total body mass is potentially misleading because adipose tissue has a fraction of the metabolic rate of other lean mass tissues. This is why I requested to see the data shown without normalization.
The authors have repeated the experiment in question, but unfortunately did not collect lean and fat mass data for normalization. It would be better to see the data without normalization (i.e. VO2 per mouse), than normalized to body weight. The authors have the data so there's no more experiments to perform. Simply create a new supplemental figure of the respirometry data without dividing by body mass. Even if the data shows that the energy expenditure phenotype disappears that is ok, the data is what it is, it leaves the door open to future investigations. The metabolic physiologist readers can form their own conclusions only if they can see the data without normalization to body mass.
Here is a very recent example of indirect calorimetry data from an anti-obesity study with respirometry data normalized per mouse: Bernier et al., 2020, Cell Metabolism 32, 203–214. The mechanism of weight loss in this paper isn't necessarily clear, which is ok, but at least the reader can see the data for what it is.

**Reply:** We sincerely appreciated the reviewer for the comment and providing the very recent published paper for reference. As instructed by the reviewer, we have expressed the data in both ways. We supplemented metabolic data of each mouse without normalization to body weight in the revised manuscript (Fig.6, D to F in the revised manuscript) and displayed the data with normalization to body weight in the supporting information (fig. s15, A to C in the supporting information), which will facilitate the reader to comprehensively consider the effect of IR-61 on energy expenditure. We also have discussed this issue and cited the recommended article (Cell Metab. 2020, 32, 203) according to the comment by the reviewer in the revised manuscript (line 281-287 in the revised manuscript). Regarding the protocol of indirect calorimetry, mice were fed a HFD for 12 weeks to induce obesity and then treated with IR-61 or vehicle control for 2 weeks before carrying out metabolic analysis and we have added this information in the revised manuscript.

Reviewer #1 (Remarks to the Author):

The indirect calorimetry data is one of the more important pieces of data in the manuscript, and it is very nice to see it presented clearly. Nice job.
